# Progress in ICP-MS Analysis of Minerals and Heavy Metals in Traditional Medicine

**DOI:** 10.3389/fphar.2022.891273

**Published:** 2022-06-28

**Authors:** Wanyue Chen, Yichu Yang, Ke Fu, Dewei Zhang, Zhang Wang

**Affiliations:** ^1^ College of Pharmacy, Chengdu University of Traditional Chinese Medicine, Chengdu, China; ^2^ State Key Laboratory of Southwestern Chinese Medicine Resources, Chengdu University of Traditional Chinese Medicine, Chengdu, China; ^3^ Chongqing Wanzhou Institute for Food and Drug Control, Chongqing, China; ^4^ College of Ethnomedicine, Chengdu University of Traditional Chinese Medicine, Chengdu, China

**Keywords:** ICP-MS, ICP-MS combined technology, mineral, heavy metal elements, medicinal materials, Chinese patent medicine, traditional medicine

## Abstract

**Aim:** This study systematically reviewed the application of ICP-MS and its combined technology in the determination of mineral and heavy metal elements in medicinal materials derived from plants, animals, minerals and their preparations (Chinese patent medicine), and biological products. It provides a reference for improving the quality standard of traditional medicine and exploring the effective components, toxic components, and action mechanism of traditional medicine.

**Materials and Methods:** A total of 234 articles related to the determination of mineral and heavy metal elements in medicinal materials derived from plants, animals, and minerals and their preparations (Chinese patent medicine) were collected from PubMed, CNKI, Web of Science, VIP, and other databases. They were classified and sorted by the inductively coupled plasma-mass-spectrometry (ICP-MS) method.

**Results:** Of the 234 articles, 154 were about medicinal materials derived from plants, 15 about medicinal materials derived from animals, 9 about medicinal materials derived from minerals, 46 about Chinese patent medicine, 10 about combined technology application, and 3 about drugs being tested after entering the body. From the 154 articles on medicinal materials derived from plants, 76 elements, including Cu, Cd, Pb, As, Cr, Mn, and Hg, were determined, of which the determination of Cu was the most, with 129 articles. Medicinal materials derived from the roots, stems, leaves, flowers, and fruits and seeds of plants accounted for 25.97%, 18.18%, 7.14%, 7.79%, and 14.94%, respectively. Moreover, medicinal materials derived from the whole plants accounted for 14.94%, and other medicinal materials derived from plants and soil accounted for 11.04%. A total of 137 of the tested medicinal materials were from traditional Chinese medicine, accounting for 88.96%, 12 were from Arabic medicine (including Unani), accounting for 7.79%, 2 were from Tibetan medicine of China, and 1 was from Mongolian medicine of China, 1 was from Miao medicine of China, and 1 was from Zhuang medicine of China. In the 15 articles on medicinal materials derived from animals, 49 elements such as Cu, As, Cd, Hg, Se, Pb, and Mn were determined, of which Cu was the most. All the tested medicinal materials belong to traditional Chinese medicine. From the nine articles on medicinal materials derived from minerals, 70 elements such as Fe, Cu, Zn, Al, As, Se, and Na were determined, of which Fe, Cu, and Zn were the most. The tested medicinal materials all belong to traditional Chinese medicine. From the 46 articles on Chinese patent medicine, 62 elements such as Cu, As, Pb, Cd, Hg, Ni, and Cr were determined, of which Cu was the most. Regarding the tested Chinese patent medicine, 38 articles belong to traditional Chinese medicine, 6 to Tibetan medicine, and 2 to Mongolian medicine of China. Three articles determine the content of metal elements in biological samples such as animal hepatic venous blood, abdominal aortic blood, brain, liver, kidney, urine, and feces, and one article determines the content of metal elements in human lung and serum. From the 10 articles combined with liquid chromatography and gas chromatography, 16 elements such as MMA, DMA, AsIII, AsV, AsB, AsC, and AsI_3_ were determined, of which MMA and DMA were the most. It can realize elemental morphology and isotope analysis. The tested medicinal materials and Chinese patent medicine belong to traditional Chinese medicine.

**Conclusion:** ICP-MS was applied the most in traditional Chinese medicine, followed by Arabic medicine. ICP-MS was used to determine more medicinal materials derived from plants, and Cu was determined the most. The characteristic inorganic element spectrum of medicinal materials can also be established. ICP-MS and its combined technology are widely used in Chinese patent medicine, but the test of biological samples is the least. The information provided in this article can provide a reference for improving the quality standard of traditional medicines and exploring the active ingredients and toxic ingredients and their mechanism of action.

## Introduction

Traditional medicine in China includes traditional Chinese medicine, ethnic medicine (e.g., Tibetan, Mongolian, Uygur, Dai, Miao, and Zhuang medicines), and religious medicine (e.g., Buddhist and Taoist medicines). Traditional Chinese medicine is an important part of Traditional medicine in China. China is the world’s largest producer of medicinal materials. Arabic medicine is derived from Hippocratic–Galenic medicine in ancient western philosophy, formed in the Arab Empire between the 8th and 12th centuries. It is generally believed that Unani, which is popular in south Asian countries, belongs to Arabic medicine. Traditional drugs have three basic ingredients—medicinal materials derived from plants, animals, and minerals—among which medicinal materials derived from plants account for the highest proportion. Medicinal materials derived from plants have six organs (roots, stems, leaves, flowers, fruits, and seeds), which makes the medicinal parts of medicinal materials derived from plants diverse. Root and rhizome traditional drugs account for the largest proportion and are the most common in the whole medicinal materials derived from plants, so the inductively coupled plasma-mass-spectrometry (ICP-MS) method is also the most widely used in this kind of traditional drugs.

Heavy metal is not only a toxic component of common concern in today’s society but also an effective component of traditional drugs. Traditional medicinal materials derived from plants, animals, minerals, and their preparations (Chinese patent medicine) often contain several minerals and heavy metals. In addition, there are cases of heavy metals exceeding the standard in the drugs. The limitation of heavy metals and the improvement of quality control standards are major dilemmas affecting the internationalization process. People favor traditional drugs more because of their reliable curative effect, less toxic and side effects, relatively safe use, and other characteristics. However, in the process of planting, production, and processing of traditional drugs, due to their enrichment and absorption of heavy metals or the high content of metal elements in the cultivated soil, the application of chemical fertilizer and pesticide contamination and other situations may introduce metal elements, resulting in an abnormal increase in metal element residues, which also makes traditional drugs have varying degrees of heavy metal pollution (Chi et al., 2016). There are many kinds of inorganic trace elements in traditional drugs. The inorganic elements are closely related to their efficacy and therapeutic use. They are one of the effective components of traditional Chinese medicine. Studying the content and distribution of inorganic elements in traditional drugs to elucidate traditional pharmacology and toxicology and further develop medicinal resources is of great value.

ICP-MS mostly uses a quadrupole mass spectrometer, which can quickly and continuously measure the mass of different elements. Currently, it can be used to analyze more than 70 elements. The detection limit of ICP-MS for more than 70 elements in the solution is one trillion or less, and the linear dynamic range can reach nine orders of magnitude (Olesik, 2014). ICP-MS is an inorganic multi-element analysis technology with inductively coupled plasma as an ion source and mass spectrometry in the field of analytical chemistry in the early 1980s. In 1980, Robert et al. (1980) published the first article on the feasibility of ICP-MS, and the first commercial instrument came out 3 years later. So far, there are about 20 types of ICP-MS instruments commercialized worldwide. In the field of drug element analysis and safety monitoring, ICP-MS and its combined technology are also increasingly widely used, which seems to have become a common and mature analysis and detection means. There are many common methods for the determination of trace elements in traditional Chinese drugs, mainly including atomic fluorescence spectrophotometry (AFS) (Zhang et al., 2016a), atomic absorption spectrophotometry (AAS) (He et al., 2019), ultraviolet-visible spectrophotometry (UV-VIS) (Wei et al., 2019), and inductively coupled plasma atomic emission spectrometry (ICP-OES) (Ma et al., 2020). Most of these methods can determine single elements, but they can not determine some elements with low content. Metal determination can also be used for QC analysis and spectral and voltammetric configurations (Locatelli et al., 2014). Dora Melucci (Melucci et al., 2013) determined the contents of heavy metals and total mercury in *Camellia sinensis* by micro voltammetry. ICP-MS is a new element analysis technology, with the advantages of less interference, high precision, wide linear range, and fast analysis speed.

In view of the wide application of ICP-MS in traditional drugs and preparations (Chinese patent medicine), 234 relevant articles on the determination of minerals and heavy metals in plants, animals, mineral medicinal materials, and preparations by ICP-MS and its combined technology were collected and classified. The purpose is to provide a reference for improving the quality standard of traditional drugs and exploring the material basis and mechanism of efficacy/toxicity.

## Types, Sources, Toxicology, and Pharmacological Effects of Minerals and Metal Elements in Traditional Drugs

### Types of Minerals and Heavy Metals

Mineral elements are one of the nutrients needed by the animal body. At present, more than 20 mineral elements such as Ca, P, K, and S have been found in the human body. Although the content in the human body is very low, they participate in and affect the physiological metabolism of the human body and are indispensable elements for maintaining human health.

Heavy metal elements refer to metals with a density greater than 4.5 g/cm^3^, including Au, Hg, Pb, and Cr. According to international standards, heavy metal elements mainly include Cu, Cd, Pb, Hg, and As. Heavy metals cannot be biodegraded, but they can aggregate thousands of times under the biomagnification of the food chain and finally enter the human body to interact strongly with proteins and enzymes, making them inactive. It may also accumulate in some organs and tissues of the human body, and various poisoning symptoms will appear. In addition, Cu is one of the essential trace elements of the human body, and it participates in important physiological processes of the human body. The lack of Cu will cause a decrease in brain cytochrome oxidase, which can lead to thinking disorder, slow response, and dyskinesia.

### Sources and Pathways of Heavy Metals

In recent years, with the development of the mining industry, mining dust and ore washing water pollute farmland, resulting in the enrichment of heavy metals in agricultural products. The sources of heavy metals in traditional Chinese medicine and Tibetan medicine should be closely related to their planting conditions and growth environment, such as the application of soil, atmosphere, water, chemical fertilizer, and pesticide. The “three industrial wastes”, including waste gas, waste water and waste residue, directly or indirectly pollute traditional Chinese medicine and Tibetan medicine. Moreover, the genetic characteristics of plants, such as active absorption function and enrichment ability of heavy metals, are related to the production and content of heavy metals. Most bases for medicinal materials from plants are formed by the transformation of farmland, which will cause the slow accumulation of heavy metals due to long-term garbage accumulation (e.g., batteries and metal waste), pesticide application, proximity to mines, and many other factors. However, medicinal materials derived from the roots of plants are medicinal materials with roots or roots as the main part and some rhizomes. They are closely related to the growth environment and conditions, and different parts of medicinal materials have different abilities to absorb and enrich heavy metals. In addition, during the processing of traditional Chinese medicine and Tibetan medicine, the use of metal processing tools and the addition of medicinal accessories may lead to the introduction of heavy metal elements, especially the processing of precious drug preparations in Tibetan medicine preparations, which will introduce a large number of heavy metal elements. It can be seen that heavy metals are dangerous to be introduced in the growth, collection, transportation, processing, and preparation of medicinal materials. Some studies have shown that the contents of heavy metals and harmful elements in the same medicinal material from different producing areas can differ by nearly 10 times at most. Such residual impurities are highly toxic. After entering the body, they can form complexes or metal chelates with traditional Chinese medicine components in the body, such as protein and nucleic acid, which have very strong toxicity (Xu et al., 2017). Much evidence shows that all kinds of pesticides and heavy metals are carcinogenic. If they do not act directly, there is also evidence that these preparations can participate in carcinogenesis in a passive or allowable way to promote the formation of tumors induced by other preparations. Through chemical interactions with the environment and each other, metal pesticide mixtures may produce unpredictable toxicity, such as organochlorine pesticides that are fat-soluble and easy to accumulate in the fat body, resulting in nerve, liver, and kidney damage. Heavy metals cause protein denaturation in the body and impair the function of tissue cells (Wang et al., 2020a).

### Toxicological and Pharmacological Effects of Heavy Metals

The strong binding of -SH and -S-S- bonds on human enzyme proteins with heavy metal elements is the main molecular mechanism of human poisoning (Zhao, 1991). When the minerals and heavy metals in the human body are within the acceptable limit of the human body, they will not cause harm to the human body. However, when they accumulate to a certain extent, they will inevitably cause irreparable harm to the human body. As a toxic heavy metal element, Cd is a non-essential trace element in the human body. It is mainly absorbed by plants in water-soluble and exchangeable states and then enriched into the human body through the food chain or directly into the human body through the respiratory system and digestive system, which accumulates in the body for a long time and damages organ functions. Specific manifestations are lung injury, kidney failure, gastrointestinal stimulation, cardiovascular and cerebrovascular diseases, muscle pain, bone pain, and bone atrophy, as well as carcinogenic, teratogenic, and mutagenic effects; long half-life in the body; and irreversible damage (Guan et al., 2021). Tl and its compounds have high toxicity and strong accumulation. They are strong neurotoxicants and can cause liver and kidney damage. They also have mutagenic, teratogenic, neurotoxic, and reproductive toxic effects. Long-term exposure to arsenic can lead to chronic poisoning, manifested as skin pigmentation, hyperkeratosis, or verrucous hyperplasia, as well as leukopenia or anemia. Ag poisoning seriously affects the human central nervous system and paralyzes the limbs. In severe cases, it can lead to heart failure and death as exposure can lead to different degrees of liver injury, fibrosis, liver cirrhosis, and even liver cancer (Huo et al., 2016). The half-life of mercury can reach 240 days in brain tissue and 70 days in other organs, so its toxic effect is toxic dose-response. The acute toxicity caused by mercury is mainly liver damage. In contrast, the chronic toxicity caused by long-term medication is more likely to cause damage to the kidney, liver, and brain. Al can cause neurofibrillary tangles and amyloid senile plaques in brain tissue, which is related to Alzheimer’s disease (AD). Long-term excessive aluminum intake can lead to an imbalance in the human body and a decline in cognitive ability, memory ability, and logical reasoning ability (Chen et al., 2011a). Pb can induce brain cell apoptosis and inhibit the activity of brain cell enzymes, thus interfering with the metabolism of neurotransmitters, protein kinase activity, and calcium metabolism. Pb exposure may lead to postural coordination disorders. Cu is a trace element required by the human body, which plays a role in promoting the generation and maturation of red blood cells, but excessive intake may cause poisoning, which will cause hypotension, jaundice, acute copper poisoning, hepatolenticular degeneration, intrahepatic cholestasis in children, and other diseases (Shen et al., 2017). Th is related to the occurrence and development of various malignant tumors, such as esophageal cancer, gastric cancer, nasopharyngeal carcinoma, and cervical cancer. U element and its compounds can damage DNA, induce ultrastructural changes in the cell membrane, and lead to tumors.

Heavy metals also have various pharmacological effects. For example, Tibetan medicine Zuotai is made from mercury, but it has antidepressant and anxiolytic effects (Zhao et al., 2016a; Zhao et al., 2016b). In addition, Cu is one of the heavy metal elements and the component of copper-containing protein in the human body. It can catalyze the synthesis of hemoglobin. However, if it exists in the form of Cu^2+^, it will become an excellent catalyst for redox reaction *in vivo* (Gao et al., 1993). Cu is absorbed from the small intestine and combined with plasma protein to form ceruloplasmin, which is mainly synthesized in the liver, then discharged with bile, and stored in the liver, bone, and muscle of the human body, with iron oxidase and antioxidant effects (Shao and Zhang, 2017). It can be seen that heavy metal elements are also toxic and medicinal. Many medicinal preparations containing heavy metals may be the material basis of efficacy, which are relative. For example, in the view of traditional Chinese medicine, drugs are toxic, they are partial, and there must be drugs if there is great toxicity. Therefore, excessive accumulation of heavy metals may be quite harmful to the body, but appropriate use or different valence forms, or the effect of this heavy metal is just needed by the disease so that it can become a good medicine.

Fe element has a hematopoietic function in the human body; participates in synthesizing hemoglobin, myoglobin, cytochrome, and various enzymes; promotes growth and development; and transports oxygen and nutrients in the blood (Guo et al., 2015). It is related to the pathogenesis of Alzheimer’s disease, Parkinson’s syndrome, and osteoporosis (Zheng et al., 2016). Hexavalent chromium is carcinogenic for humans. Indeed, extensive literature demonstrates the carcinogenic effects of chromium (VI) (Locatelli et al., 2014). Zn is a component of many enzymes in the human body and participates in synthesizing DNA and RNA polymerase. It is an important element in maintaining the integrity of skin and mucosa and promoting wound healing. At the same time, it can increase lymphocyte function and remove oxygen-free radicals in the body. It can prevent bacterial and virus invasion and anti-cancer. When the body lacks Zn, it will cause aging of tissue cells and a decline in immunity, and epithelial cells are vulnerable to carcinogens, resulting in carcinogenesis (Wang and Jin, 2018). However, Zn cannot be synthesized in the body and must be supplemented through dietary regulation. When the supply is insufficient or the proportion is unbalanced, it can directly affect the normal growth and development of children. Cr is involved in the metabolism of sugar and fat in the human body. It is also an essential element of normal cholesterol metabolism and will cause liver lipid metabolism disorder. Low chromium is a risk factor for obesity and disorder of glucose and lipid metabolism (Yang et al., 2015). As the main component of cinnabar, HgS can inhibit bacteria, reduce inflammatory reactions, and promote wound healing. It is mainly used to treat sores and swelling. Ca is a major element in animals. It can help blood clot and activate some enzymes in the body, maintain nerve conduction performance, and play an essential role in maintaining the physiological function of the liver. K can maintain water balance, osmotic balance, and acid-base balance in the human body; strengthen the excitability of muscles; maintain the rhythm of the heartbeat; and participate in the metabolism of protein, carbohydrates, and heat energy. Mg can activate various enzymes in the body, maintain the stability of the nucleic acid structure, inhibit nerve excitability, and participate in protein synthesis, muscle contraction, and body temperature regulation. As an essential trace element in the human body, Mn plays a regulatory role in human life activities by forming binding proteins, enzymes, hormones, and vitamins. Arsenic trioxide can treat leukemia by inducing apoptosis of leukemia cells; inhibit proliferation and induce apoptosis of lung cancer cells; induce apoptosis of gastric cancer cells, colon cancer cells, and cervical cancer cells; and significantly inhibit the growth and apoptosis induction of oral squamous cell carcinoma cells. It can also enhance the inhibitory effect of promyelocytic leukemia on cell proliferation, invasion, and migration and may be used as a new therapeutic target for breast cancer. It has potential therapeutic value for triple-negative breast cancer. As a metal auxiliary group, Mn is necessary for the activity of superoxide dismutase and an important member of the liver antioxidant system. Mn is also closely related to reproduction, which can promote cholesterol synthesis, promote human growth and development, and enhance reproductive function (Yu et al., 2015). Ni participates in the metabolism of the body and the composition of the cell membrane. It can activate histidine enzyme, arginase, acid phosphatase, and other important enzymes in the body, maintain the stability of biological macromolecular structure and normal metabolism of the whole body, and participate in the composition of various enzymes and proteins in the liver. The change of Ni content has a certain relationship with the occurrence and development of bronchial asthma. Se is one of the important components of glutathione peroxidase, a non-specific antioxidant of red blood cells. Its main function is to remove peroxides and free radicals from the human body and play a positive role in the treatment of cancer, cardiovascular diseases, diabetes, and other diseases. Se deficiency can easily lead to liver disease and liver injury (Zhao et al., 2017a).

**FIGURE 1:**
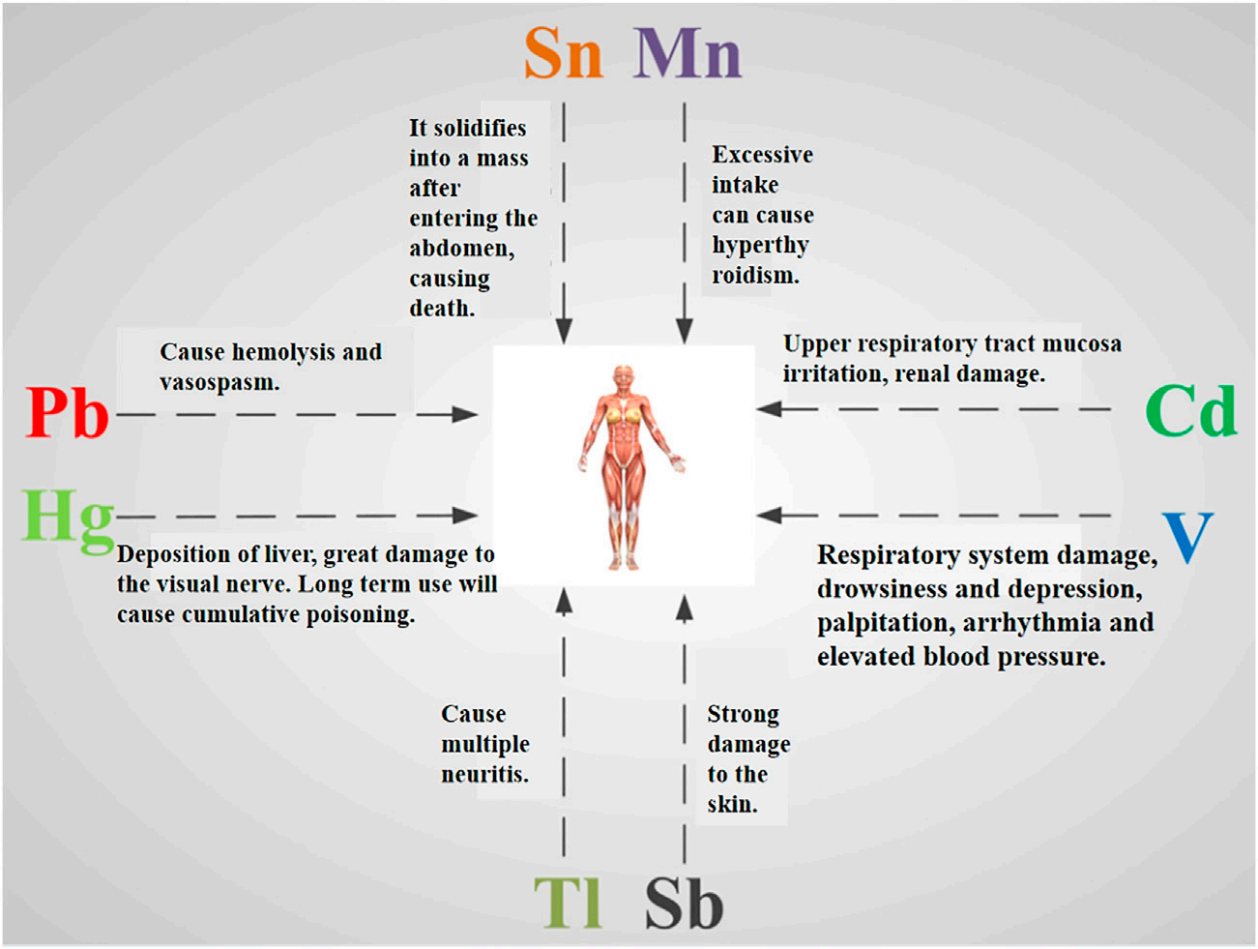
Symptoms of heavy metal poisoning.

Heavy metals are not only the toxic ingredients of today’s society but also the effective ingredients of traditional drugs. The key is to master the “dose, the length of drug use, and the pathological state of the human body” to balance toxicity and effectiveness. One of the major challenges facing traditional drugs containing minerals is differences in mineral processing/preparation that distinguish them from environmental metals. Additionally, many preparations are polyherbal-metallic preparations, which increases their complexity because minerals are not used alone. Symptoms of heavy metal poisoning are shown in Figure 1. Furthermore, toxicity and therapy go hand-in-hand with these preparations, and there exists a need to maintain a subtle balance of benefit and risk on an individual basis. Therefore, ICP-MS provides a powerful means of detection to achieve this goal.

## Analysis and Application of ICP-MS in Medicinal Materials Derived From Plants

Excessive heavy metal elements in traditional medicine as the biggest restriction factor of its application (Fan et al., 2018; He et al., 2011), essential trace elements affecting the effect of traditional Chinese medicine, and heavy metals as generally toxic elements should be within the prescribed limit standards. Therefore, the analysis of trace elements and heavy metals in traditional medicines can provide more references for the efficacy, property, and safety of traditional Chinese medicine. ICP-MS has many advantages, such as less interference, wide linear range, high precision, low detection limit, and simultaneous determination of multiple elements. In addition, ICP-MS technology integrates with various different separation technologies to scientifically analyze the forms and valence states of trace elements and heavy metals (Pietilä et al., 2015; Schmidt et al., 2017). It can provide accurate isotopic information and determine the isotopic ratio, which is unavailable in other element analysis methods. The processing of traditional drugs is the characteristic of traditional medicine, and its main purpose is to increase efficiency and reduce toxicity. Traditional medicine inorganic elements can be used as indicators to help explain the changes in material basis before and after processing. Tracking the content changes of inorganic elements, especially the content changes of harmful elements, is helpful to further clarify the processing mechanism of traditional Chinese medicine and the influence of its nature, taste, and Meridian on clinical effect. Luo et al. (2015a) used the ICP-MS technology to compare and analyze the inorganic element content of raw *Reynoutria multiflora* (Thunb.) Moldenke and its processed products. It was found that after processing, compared with non-processed products, the content of most inorganic elements increased, whereas the content of some harmful metal elements decreased.

**FIGURE 2:**
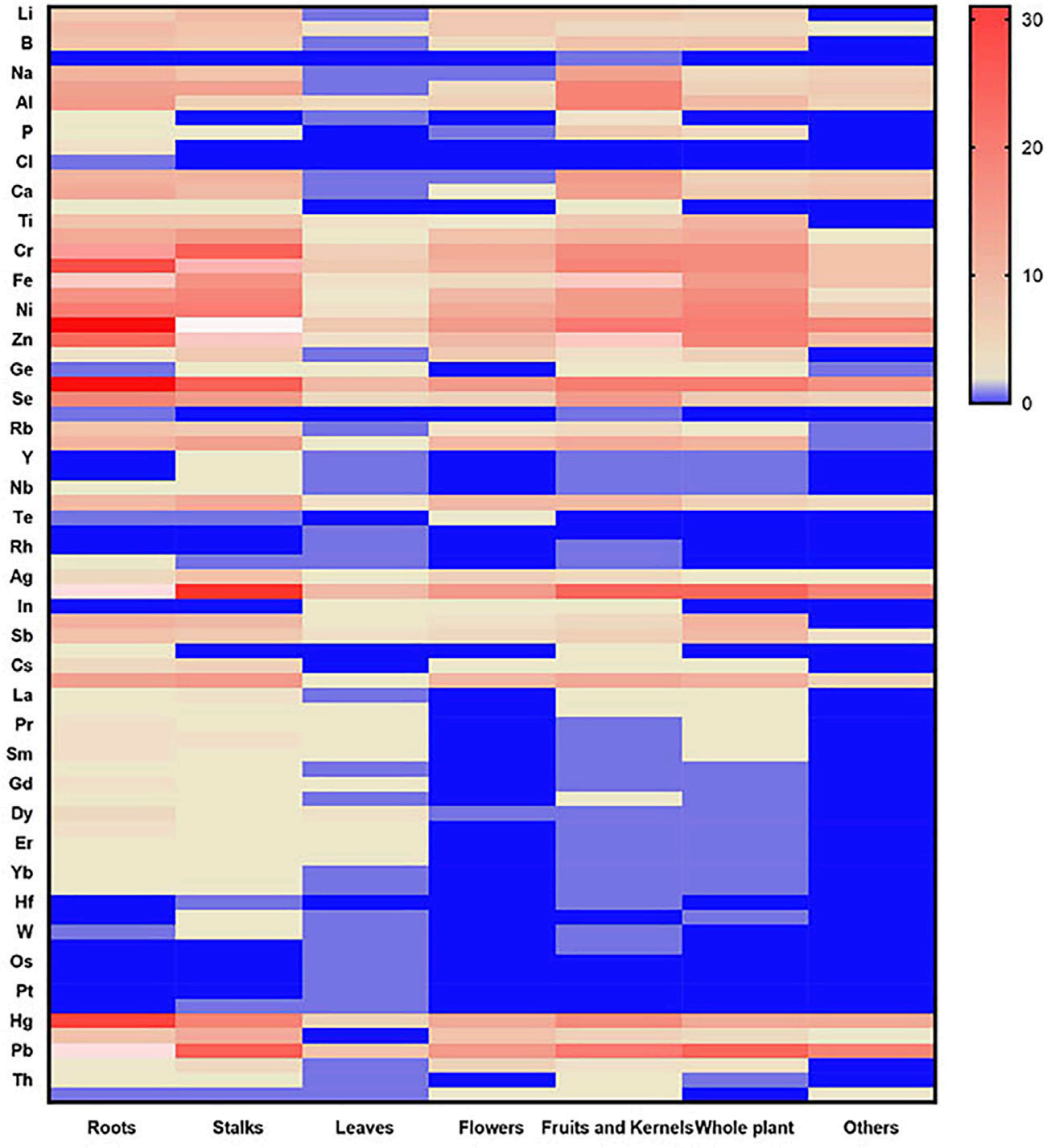
Heat map of heavy metal elements measured by ICP-MS of medicinal materials derived from plants in different medicinal parts.

Medicinal materials from plants include roots and rhizomes, stems, leaves, flowers, fruits and seeds, whole plants, and other medicinal parts. With the development and progress of science and technology and the in-depth application and research of pharmaceutical analysts, the ICP-MS technology will continue to play a more important role in the field of traditional drugs with its own unique advantages. Liu et al. (2018a) determined the contents of eight heavy metals and harmful elements in 10 kinds of medicinal materials from different sources, including *Dioscorea nipponica* Makino, *Sanguisorba officinalis* L., *Curcuma phaeocaulis* Valeton, *Conioselinum anthriscoides* (H. Boissieu) Pimenov & Kljuykov, *Ephedra equisetina* Bunge, *Euchresta japonica* Hook.f. ex Regel, *Clematis chinensis* Osbeck, *Inula helenium* L., *Allium macrostemon* Bunge, and *Aster tataricus* L.f. The contents of heavy metals and harmful elements in medicinal materials are affected by many factors, such as origin, environment, and soil. It is necessary to strengthen supervision, control, and strict norms to ensure the quality and drug safety of traditional Chinese medicine and provide a reference basis for the formulation of safety standards of traditional Chinese medicine. Other applications of ICP-MS in medicinal materials derived from plants are shown in Tables 1–7. From the 154 articles on medicinal materials from plants, 76 elements including Cu, Cd, Pb, As, Cr, Mn, and Hg were determined, of which 129 were Cu, accounting for 83.77%. A heat map of heavy metal elements measured by ICP-MS of medicinal materials derived from plants in different medicinal parts is shown in Figure 2. From the 154 articles on the application of ICP-MS in medicinal materials from plants, root, stem, leaf, flower, fruit and seed, and whole grass medicinal materials derived from plants accounted for 25.97%, 18.18%, 7.14%, 7.79%, 14.94%, and 14.94%, respectively, and other medicinal materials derived from plants and soil accounted for 11.04%. Of the 154 articles of botanical medicine, 137 belong to traditional Chinese medicine, accounting for 88.96%; 12 belong to the medical system of Saudi Arabia, accounting for 7.79%; 2 belong to the medical system of Tibetan medicine; 1 belongs to the medical system of Mongolian medicine; 1 belongs to Miao medicine; and 1 belongs to Zhuang Medicine in China. At present, ICP-MS has been widely used to analyze the contents of trace and ultra-trace inorganic elements in traditional Chinese medicine. Using the advantages of simultaneous determination of multiple elements by ICP-MS, we can quickly implement the quality control of various inorganic elements in traditional Chinese medicine, further ensuring the safety of traditional Chinese medicine.

**TABLE 1:** Application of ICP-MS in the determination of heavy metal elements in medicinal materials derived from the roots of plants.

Latin name	Medical system	Therapeutic use	Main pharmacological effects	Origin of medicinal materials^a^	Test results
*Achyranthes bidentata* Blume	Traditional Chinese medicine	Amenorrhea, dysmenorrhea, headache, toothache, hematemesis, bleeding	Immune regulation, anti-fertility, anti-inflammatory, antibacterial, analgesic, anti-osteoporosis, and anti-aging	—	The contents of rare earth elements La, Tb, Ce, Dy, Pr, Ho, Nd, Er, Sm, Tm, Eu, Yb, Gd, and Lu in *A. bidentata* Blume were determined. The content of Ce was the most and Lu was the least. The rare earth elements with a content of more than 100 ng/g (DW) are La, Ce, and Nd. La is 158.270 ng/g (DW), Ce is 448.698 ng/g (DW) and 129.608 ng/g (DW). Rare earth elements Pr, Sm, and Gd > 20 ng/g (DW). The contents of Eu, Tb, Dy, Ho, Er, and Yb are 2.536–14.522 ng/g (DW). The content of Tm and Lu is lower than 1 ng/g (DW), Tm is 0.918 ng/g (DW), and Lu is 0.848 ng/g (DW) (Xu et al., 2008)
*Aconitum kusnezoffii* Rchb.	Traditional Chinese medicine	Arthralgia, cardialgia	Analgesic, anti-inflammatory, cardiotonic, anti-tumor, immune regulation	Sichuan, Xinjiang, Jilin, Shandong, Shaanxi, Anhui, Henan, Hunan, Guizhou, Inner Mongolia, and Hebei provinces or autonomous regions of China	The contents of Pb, Cd, Cr, Cu, Hg, and As, six heavy metals in *A. kusnezoffii* Rchb., prepared from different producing areas such as Sichuan, Shaanxi, Hunan, and Inner Mongolia were determined. The isotopes 208 Pb and 202 Hg were selected with 209 Bi as internal standard, 114 Cd with 115 In as internal standard, 63 Cu and 52 Cr with 47 Sc as internal standard, and 75 As with Ge as internal standard. Cd, As, and Hg in *A. kusnezoffii* Rchb. prepared from some producing areas exceeded the standard. The linear correlation coefficients of the six metal elements were greater than 0.9985, the average recovery was 83.99%–97.96%, and the RSD was 0.94%–2.66% (Hu et al., 2020)
*Actaea cimicifuga* L.	Traditional Chinese medicine	Headache, pharyngalgia, measles	Anti-virus, anti-tumor, regulating neuroendocrine function, anti-osteoporosis, and anti-inflammatory	—	The contents of Be, Al, Cr, Mn, Ni, Cu, As, Se, Mo, Ag, Cd, Sn, Sb, Ba, Dy, Hg, Tl, and Pb in *A. cimicifuga* L. were determined. The calibration curves of 18 elements have a good linear relationship, and the recovery meets the requirements of trace analysis (Xiao et al., 2013)
*Anethum graveolens* L.	Traditional Chinese medicine	Spasm	Analgesic, anti-inflammatory, antibacterial, antioxidant, anti-tumor, anti-diabetes	Bangladesh	The contents of Ca, Mg, Ni, Se, Li, Na, and Fe in *A. graveolens* L. were determined. Ca (23,600 mg/kg), Mg (7620.33 mg/kg), and K (1286.15 mg/kg) were the most important elements, followed by Ni (1187.30 mg/kg), Se (913.79 mg/kg), Li (317.84 mg/kg), Na (288.72 mg/kg), and Fe (206.88 mg/kg). Toxic elements are within the allowable limits (Saleh-E-In et al., 2017)
*Angelica dahurica* (Hoffm.) Benth. & Hook.f. ex Franch. & Sav.	Traditional Chinese medicine	Common cold due to wind-cold, headache, toothache, nasosinusitis	Antipyretic, analgesic, anti-inflammatory, and antiviral microorganisms	Anhui province of China	The contents of Pb, Cu, As, Cd, and Hg in *A. dahurica* (Hoffm.) Benth. & Hook.f. ex Franch. & Sav. were determined. The linear relationship was good. The RSD value of the precision test was 1.99%–4.89%, the RSD value of the repeatability test was 1.31%–5.09%, and the average spiked recovery was 83.95%–103.13%. The contents of Pb, Cd, Hg, As, and Cu in 12 samples did not exceed the standard (Wang et al., 2015a)
*Angelica sinensis* (Oliv.) Diels	Traditional Chinese medicine	Irregular menstruation, amenorrhea, dysmenorrhea, constipation, falling, and fluttering injury	Analgesic, anti-inflammatory, antibacterial, antioxidant, anti-senile dementia	—	1) The contents of Mn, Cr, Sr, Zn, Rb, B, Ni, V, Sn, Mo, Se, Co, Cu, As, Pb, Cd, and Hg in three different batches of *A. sinensis* (Oliv.) Diels were determined. The linear relationship of 17 trace elements was good in the range of 0.05–150.00 g/L, the correlation coefficient R was greater than 0.999, the detection limit of each element was 0.0018–0.1203 g/g; the RSD of precision, repeatability, and stability tests were not greater than 3.95%, 2.93%, and 3.42%, and the recovery was 96.67%–100.48%. *A. sinensis* (Oliv.) Diels is rich in Mn, B, Zn, Sr, Cr, Rb, and other essential trace elements for the human body, and the content of five heavy metal elements does not exceed the standard (Wang et al., 2019a). 2) The contents of Pb, Cd, Hg, and As in *A. sinensis* (Oliv.) Diels were determined. The recovery was 99.2%–102.4%, the RSD range was 1.01%–1.21%, and the detection limit was 0.005–0.018 μg·L^−1^ (Rong and Zhang, 2017)
*Asarum heterotropoides* F. Schmidt	Traditional Chinese medicine	Headache, stuffy nose, and runny nose caused by a cold	Antiviral, antibacterial, anti-inflammatory, antioxidant, and anti-allergic	Liaoning, Gansu, Shaanxi, Sichuan, Jilin, and Heilongjiang provinces, China	Six kinds of heavy metals and harmful elements Pb, Cd, Cu, Hg, As, and Cr in *A. heterotropoides* F. Schmidt from different producing areas such as Sichuan, Shaanxi, and Gansu were determined. The contents of six kinds of heavy metals and harmful elements in Asarum from 12 different producing areas were quite different. The linear correlation coefficients of six kinds of heavy metals and harmful elements were greater than 0.998, the average recovery was in the range of 87.8%–96.0%, and the RSD value was less than 3.0% (Yi et al., 2020)
*Astragalus mongholicus* Bunge	Traditional Chinese medicine	Edematous, descensus uteri, diabetes	Anti-inflammation, immune regulation, anti-tumor, anti-stress, and liver protection	—	The contents of 27 inorganic elements of Li, Be, Na, Mg, K, Mn, Co, Se, Br, Rb, Y, Nb, Mo, Pd, Te, I, Cs, Pr, Nd, Sm, Gd, Dy, Ho, Yd, W, Tl, and Th in *A. mongholicus* Bunge were determined. The main discriminant elements are determined as K, Mg, and Na (Wang et al., 2012a)
*Chuanminshen violaceum* M. L. Sheh & R. H. Shan	Traditional Chinese medicine	Gastrosia, intoxication	Anti-fatigue and anti-oxidation	Sichuan province of China	The contents of heavy metals in 33 batches of *C. violaceum* M. L. Sheh & R. H. Shan from eight different producing areas in Sichuan, China, were determined. The amount of heavy metals in each sample of *Chuanmingshen* is Pb ≤ 0.256, Cd ≤ 0.235, Hg ≤ 0.123, Cu ≤ 3.963, and Cr ≤ 2.145 μg/g; As is not detected. Both are lower than the standards for raw materials and decoction pieces of medicinal plants in China’s green standard for the import and export industry of medicinal plants and preparations (Yue et al., 2016)
*Codonopsis pilosula* (Franch.) Nannf.	Traditional Chinese medicine	Coronary heart disease, hyperlipidemia	Anti-tumor, anti-oxidation, anti-inflammatory, anti-stress, and liver protection	Shanxi province of China	The contents of As, Hg, Pb, and Cd in *C. pilosula* (Franch.) Nannf. were determined. The correlation coefficient *r* of the measured elements and the standard curve were greater than 0.9992, the recovery was 96.5%–105.2%, and the RSD was less than 10.5% (Wang and Zhong, 2008)
*Cynanchum bungei* Decne.	Arabic medicine	Anti-tumor, antisenescence, hypolipidemia	Anti-inflammatory, anti-tumor, antidepressant, and enhancing immune function	—	1) The contents of Al, Pb, Cd, and As in *C. bungei* Decne. were determined. The recovery of all measured elements was 86.1%–90.6%. The content of Al element is the highest, and the concentration range of Al is 156–1609 mg/kg. The content of Cd is the lowest, and the concentration of Cd is in the range of 0.01–0.10 mg/kg. The washing process reduces toxic elements in all plants. The average recoveries were Al (47.32%), As (59.1%), Cd (62.03%), and Pb (32.40%) (Brima, 2017)
					2) The contents of Fe, Mn, Zn, Cu, and Se in *C. bungei* Decne. were determined. Fe level is the highest, and Se level is the lowest among all plants. The TE series levels of all elements in all plants are as follows: Fe 193.4–1757.9, Mn 23.6–143.7, Zn 15.4–32.7, Se 0.13–0.92, and Cu 11.3–21.8 μg/g. Calculated air intake of essential elements from medical plants: Fe 4.6–13.4, Mn 6.7–123.2, Zn 7.0–42.7, Se 0.14–1.5, and Cu 1.5–5.0 μg/dose (Brima, 2018)
*Glycyrrhiza uralensis* Fisch. ex DC.	Traditional Chinese medicine	Spleen deficiency, fatigue, palpitation, cough	Antibacterial, anti-inflammatory, antiviral, immune regulation, anti-asthma	Gansu, Inner Mongolia, and Xinjiang provinces or municipalities of China	The contents of 25 metal elements K, Ca, Na, Mg, Al, Fe, Se, Zn, Mn, V, Be, Ni, Ga, Rb, Sr, Cs, Ba, Tl, U, Cu, As, Cd, Pb, Hg, and Cr in *G. uralensis* Fisch. ex DC. from different producing areas in Gansu, Inner Mongolia, and Xinjiang were determined. The average recovery of each element is 85.93%–109.82%, and the RSD is less than 5% (Zhao et al., 2017b)
*Millettia pulchra* (Voigt) Kurz	Zhuang medicine in China	Fracture, deficiency of blood, dizziness, insomnia	Regulate body immunity, reduce blood pressure and resist hypoxia stress injury	Guangxi Zhuang Autonomous Region of China	Determination of five heavy metals in *M. pulchra* (Voigt) Kurz, the regression equations of Pb, Cd, As, Hg, and Cu were *Y* = 0.0130*X* + 0.0062 (*r* = 0.9997), *Y* = 0.0031*X* + 2.7982 − 4 (*r* = 0.9999), *Y* = 0.0089*X* + 4.5141 − 4 (*r* = 0.9999), *Y* = 0.0031*X* + 5.0726 − 4 (*r* = 1.0000), *Y* = 0.0985*X* + 0.0727 (*r* = 0.9999), and the recovery was 86.8%–96.3% (Bai and Hong, 2015)
*Ophiopogon japonicus* (Thunb.) Ker Gawl.	Traditional Chinese medicine	Insomnia, tumor, hyperglycemia	Protect cardiovascular system, anti-inflammatory, anti-tumor, anti-oxidation, anti-aging, and immune regulation	Sichuan province of China	The contents of Pb, Cd, Hg, As, Cr, and Cu in *O. japonicus* (Thunb.) Ker Gawl. in Fucheng, Sichuan Province, China, were determined. The linear relationship of each element is good, the correlation coefficient (*r*) is greater than 0.9990, the relative standard deviation of the precision test is less than 5%, the recovery is 91.8%–103.0%, and the detection limit is less than 0.01 mg/kg (Liu et al., 2017)
*Paeonia emodi* Royle	Unani	Antisenescence	Anti-aging	—	18 elements of Si, Cl, P, S, Sc, K, Ca, B, Ti, Na, Ni, Fe, Zn, Mg, I, Mn, Be, and Li in *P. emodi* Royle were determined. The concentration of Si was the highest (85.3 μg/g), whereas the concentration of Li was the smallest (3.0 ng/g) (Raish et al., 2016)
*Panax ginseng* C. A. Mey.	Traditional Chinese medicine	Tumor, cardiovascular disease	Anti-tumor, anti-oxidation, anti-aging, anti-diabetes, anti-hepatorenal toxicity	Jilin province of China	1) The content of metal elements in *P. ginseng* C. A. Mey. was determined, and the linearity of As, Pb, Cr, and Cd was 0–50.0 μg/L, Hg linear at 0–2.00 μg/L, Cu linear between 0 and 500 μg/L, the linearity of Al is 0–5 mg/L, the correlation coefficient is 0.9984–0.9995, the detection limit is 0.001–0.5 mg/kg, the recovery is 89.2%–104.6%, and the relative standard deviation (RSD) is 1.37%–5.61% (Wang et al., 2018c)
					2) The contents of Cr, Cu, As, Cd, Pd, and Hg in 53 samples collected from 10 major *P. ginseng* C. A. Mey. planting counties and regions in Jilin Province, China, were determined. The contents of heavy metals in ginseng samples were Cr ≤ 2.069, Cu ≤ 19.0619, As ≤ 0.0818, Cd ≤ 0.2160, and Pb ≤ 0.8501 μg/g; Hg is not detected (Chi et al., 2018)
*Panax notoginseng* (Burkill) F.H.Chen	Traditional Chinese medicine	Hypertension, traumatic injury	Anti-tumor, hypolipidemic, anti-anxiety, anti-oxidation, anti-aging	—	The contents of Cu, As, Cd, Hg, and Pb in *P. notoginseng* (Burkill) F.H.Chen were determined. The linear coefficient of the five elements in their respective linear range is *r* > 0.9995, the recovery is 92.5%–96.6%, and the RSD of the six repeatability tests is ≤ 4.38% (Xu, 2012)
*Panax quinquefolius* L.	Traditional Chinese medicine	Cardiovascular diseases	Antioxidant, anti-tumor, anti-aging, and immune regulation	—	The contents of Mn, Cr, Sr, Zn, Rb, B, Ni, Cu, V, Sn, As, Mo, Se, and Co in *P. quinquefolius* L. and garden cultivated American ginseng were determined. The linear ranges of element detection mass concentrations were 0.1–500 (*r* = 0.9998), 0.5–500 (*r* = 0.9999), 0.1–500 (*r* = 0.9999), 0.5–500 (*r* = 0.9999), 0.5–500 (*r* = 0.9999), \ 0.5–500 (*r* = 0.9999), 0.1–200 (*r* = 0.9999), 0.5–500 (*r* = 0.9999), 1–500 (*r* = 0.9999), 0.1–500 (*r* = 0.9998), 0.1–200 (*r* = 0.9998, 0.5–500 (*r* = 0.9999), 0.1–200 (*r* = 0.9998), 0.5–500 (*r* = 0.9999), and 0.1–500 μg/L (*r* = 0.9999): the quantitation limits are less than 0.60 μg/L and the detection limits are less than 0.20 μg/L. RSD was less than 3%. The recovery was 97.08%–106.65% (RSD was 0.64%–2.33%, *n* = 9) (Lin et al., 2018)
*Pseudostellaria heterophylla* (Miq.) Pax	Traditional Chinese medicine	Inappetence, cough	Anti-inflammation, anti-tumor, anti-oxidation, hypoglycemic, immune regulation	Fujian province of China	The contents of Mg, Ca, Mn, Fe, Cu, Zn, and Se in *P. heterophylla* (Miq.) Pax from Zherong County, Fujian province, China, were determined. The recoveries of each element were 92.11%–107.51%, and the RSD was less than 5.0% (Tao, 2019)
*Pueraria montana* var. *thomsonii* (Benth.) M. R. Almeida	Traditional Chinese medicine	Heartache, stroke hemiplegia	Antipyretic, analgesic, and anti-inflammatory	—	The contents of 17 trace elements As, Be, Cd, Co, Cu, Mn, Mo, Ni, Pb, Sb, Se, Ti, Tl, V, Cr, and Zn in stewed *P. montana* var. *thomsonii* (Benth.) M. R. Almeida were determined. The linear relationship of trace elements is good, *r* = 0.9990–0.9999, the detection limit is 0.000493–0.369619 ng/ml, and the recovery is 96.6%–101.9% (Chen et al., 2019a)
*Pueraria edulis* Pamp.	Traditional Chinese medicine	Measles	Anti-inflammatory, anti-oxidation, anti-tumor, anti-diabetes	Anhui, Guangxi, Hunan, Henan, Hebei, Zhejiang, Sichuan, Guizhou, Jiangxi, and Hubei provinces or autonomous regions of China	The contents of Cr, Ni, As, Cd, Pb, and Hg in 20 batches of *P. edulis* Pamp. were determined. The average content of heavy metals in *P. edulis* Pamp. is Cr (3.28 mg/kg), Ni (1.83 mg/kg), As (0.31 mg/kg), Cd (0.23 mg/kg), Pb (1.85 mg/kg), and Hg (0.07 mg/kg). The contents of Cr, Pb, and Ni in *P. edulis* Pamp. from different producing areas and different batches are quite different, whereas the contents of As, Hg, and Cd are not different. The average content of Cr is the highest, and the average content of Hg is the lowest. The total amount of Pb, As, and heavy metals in each sample meets the current standards (Zhong et al., 2014)
*Rehmannia glutinosa* (Gaertn.) DC.	Traditional Chinese medicine	Hematemesis, swelling, and pain in the throat	Antibacterial, anti-tumor, anti-gastric ulcer, anti-aging	—	The contents of Ca, Cr, Fe, Al, Zn, Ni, Ti, Cu, Mn, Rb, Pb, Sn, Co, and Mo in raw and cooked *R. glutinosa* (Gaertn.) DC. were determined. The order of metal element content in *R. glutinosa* (Gaertn.) DC. from high to low is Ca > Cr > Fe > Al > Zn > Ni > Ti > Cu > Mn > Rb > Pb > Sn > Co > Mo. The recoveries of Pb, Cu, Mn, Ni, Ti, and Zn were 87.2%, 106.8%, 96.5%, 89%, 80.4%, and 108.6%, respectively. The contents of Ca, Fe, Al, Zn, and Cu in cooked *R. glutinosa* (Gaertn.) DC. are high (Wang et al., 2009)
*Reynoutria japonica* Houtt.	Traditional Chinese medicine	Amenorrhea, traumatic injury, cough, constipation	Antibacterial, anti-inflammatory, antiviral, anti-tumor, improving Alzheimer’s disease	Guangxi Zhuang Autonomous Region of China	The contents of 28 elements Li, B, Na, Mg, Al, K, Ca, Ti, V, Cr, Mn, Fe, Co, Ni, Cu, Zn, As, Se, Sr, Mo, Ag, Cd, Sn, Sb, Ba, Tl, Pb, and Bi in *R. japonica* Houtt. were determined. The detection limit of each element is 0.01–0.8 μg/L, the standard deviation is 0.11%–2.57%, and the recovery is 94.00%–110.00% (Duan et al., 2012a)
*Reynoutria multiflora* (Thunb.) Moldenke	Traditional Chinese medicine	Dizziness, constipation	Anti-aging, anti-tumor, hypolipidemic, anti-atherosclerotic	Henan, Shaanxi, Gansu, Anhui, Sichuan, Hubei, Yunnan, Guizhou, Guangxi, and Guangdong provinces or autonomous regions of China	1) The contents of Mg, K, Ca, Na, Al, Cr, Mn, Fe, Ni, Zn, Rb, Sr, Ba, Li, Be, Sc, V, Co, Ga, Ge, Se, Y, Nb, Cs, La, Ce, Pr, Nd, Sm, Eu, Gd, Tb, Dy, Ho, Er, Tm, Yb, Lu, Tl, and Th in *R. multiflora* (Thunb.) Moldenke were determined. The linear correlation coefficients of the standard curve were greater than 0.95, and the average recovery was 88.9%–119.2%, RSD < 12%. The contents of K, Ca, Mg, Al, and Fe in 49 batches of *R. multiflora* (Thunb.) Moldenke samples were the highest, all of which were more than 100 mg/kg. The average contents of Be, Sc, Ge, Eu, Tb, Ho, Tm, Yb, and Lu are lower, all less than 0.02 mg/kg (Yang et al., 2019)
					2) The contents of 24 elements K, Fe, Al, Mg, Ca, P, Sn, As, Zs, Sb, Pb, Co, Cd, Ni, Ba, B, Si, Hg, Mn, Cr, V, Cu, Be, Na, and Sr in 33 samples of *R. multiflora* (Thunb.) Moldenke from different producing areas such as Sichuan, Guizhou, Yunnan, Jiangsu, and Hubei were determined. The amount of Fe, Mg, Ca, and P is high (Luo et al., 2015b)
*Rhodiola rosea* L.	Traditional Chinese medicine	Heartache, stroke hemiplegia	Anti-inflammation, anti-oxidation, anti-fatigue, and anti-cancer	—	The contents of 13 inorganic elements Na, Mg, Al, K, Ca, Cr, Mn, Fe, Ni, Cu, Zn, As, and Pb in *R. rosea* L. were determined. The elements with higher mass fractions in *R. rosea* L. are Ca, K, and Mg, whereas the mass fractions of As and Pb are lower than the industry standard (Wang and Zhang, 2013)
*Rosa laevigata* Michx.	Traditional Chinese medicine	Spermatorrhea, enuresis, diarrhea, uterine prolapse	Anti-inflammatory, antibacterial, anti-tumor, and immune regulation	Guangxi Zhuang Autonomous Region of China	The contents of 22 metal elements Al, Fe, Cu, Pb, As, Cd, Cr, Ni, V, Sb, Sn, Tl, Ag, B, Ba, Co, Mn, Mo, Se, Sr, Ti, and Zn in 45 batches of *R. laevigata* Michx. roots in Guangxi were determined. The content of Al element is high, Pb ≤ 5.0, Cd ≤ 0.3, Hg ≤ 0.2, Cu ≤ 20.0, and As ≤ 2.0 mg·kg^−1^, all within the standard limit (Wei et al., 2021)
*Rubia cordifolia* L.	Traditional Chinese medicine	Inflammation, tumor	Anti-inflammation, anti-tumor, anti-infection, neuroprotection	Shanxi province of China	The contents of Cu, Mn, Cr, Zn, Cd, Fe, Pb, Hg, and As in wild *R. cordifolia* L. from different habitats in Shaanxi, China, were determined. There are differences in the contents of heavy metals in wild *R. cordifolia* L. from different producing areas. The contents of Cu, Cd, Pb, Hg, and As are lower than the limit standard of heavy metals (Peng et al., 2018)
*Salvia miltiorrhiza* Bunge	Traditional Chinese medicine	Irregular menstruation, insomnia, stenocardia	Anti-inflammatory, antidepressant, anti-tumor, anti-fibrosis, anti-hepatitis B virus	Anhui province of China	1) The contents of Pb, Cd, As, Hg, and Cu in *S. miltiorrhiza* Bunge were determined. The correlation coefficients *r* of the standard curves of five heavy metals were 0.9992, 0.9995, 0.9997, 0.9994, and 0.9991, respectively, and the recoveries were 91.1%–102.5%(Li et al., 2016a)
					2) The detection limit of Pb, Cd, As, Cu, and Hg in *S. miltiorrhiza* Bunge in Anhui ancient city of China was 0.006–0.072 μg/L, and the linear relationship was *r* > 0.954. The recovery was 91.53%–100.59% (RSD < 4.97%) (Jin et al., 2018)
*Sargentodoxa cuneata* (Oliv.) Rehder & E. H. Wilson	Traditional Chinese medicine	Amenorrhea, dysmenorrhea	Antibacterial, antiviral, anti-inflammatory, anti-tumor, immunosuppressive	Hunan, Anhui, and Zhejiang provinces of China	Determination of 23 elements Na, Mg, Al, K, Ca, V, Cr, Mn, Fe, Co, Ni, Cu, Zn, As, Se, Rb, Ag, Cd, CS, Ba, Hg, and Pb in *S. cuneata* (Oliv.) Rehder & E. H. Wilson in Hunan, Anhui, and Zhejiang provinces of China. For the measured elements, the correlation coefficient of the standard curve is *r* > 0.9987, and the relative standard deviation of the method accuracy experiment is RSD < 9.1% (Liu et al., 2013)
*Scutellaria baicalensis* Georgi	Traditional Chinese medicine	Jaundice, cough	Antibacterial, anti-inflammatory, anti-tumor, cardiovascular and cerebrovascular protection, anti-organ fibrosis	Beijing and Jiangsu, Heilongjiang, Gansu, Shandong, Shanxi, Hebei, and Shaanxi provinces or municipalities of China	The contents of 23 inorganic elements Al, As, B, Ba, Be, Cd, Co, Cr, Cu, Fe, Ga, Hg, Li, Mn, Ni, Pb, Sb, Sn, Sr, Ti, Tl, V, and Zn in 35 batches of *S. baicalensis* Georgi stems and leaves from eight producing areas were determined. The contents of Al and Fe are the highest, followed by B, Ti, Mn, Sr, and Ba (Yan et al., 2018)
*Sophora tonkinensis* Gagnep.	Traditional Chinese medicine	Toothache, cough, jaundice, constipation, hemorrhoids	Anti-inflammation, anti-tumor, liver protection, and immunity enhancement	Guangxi Zhuang Autonomous Region of China	The contents of heavy metals Zn, Ba, Cu, Cr, Ni, Pb, As, Sb, Co, Cd, Sn, Ti, Bi, and Hg in the roots of *S. tonkinensis* Gagnep. in Guangxi, China, were determined. The detection limit is 0.0014–0.4241 Lg·L^−1^, the recovery is 91.3%–106.5%, and the RSD value is 0.43%–3.07%. The contents of heavy metals in the roots of kidney beans from different producing areas were basically consistent: Zn > Ba > Cu > Cr > Ni > Pb > As > Sb > Co > Cd > Sn > Tl > Bi > Hg (Gu et al., 2013)
*Stellera chamaejasme* L.	Traditional Chinese medicine	Tumor, lymphonodus, psoriasis	Anti-cancer, anti-leukemia, antibacterial and antiviral	—	The contents of 12 trace elements A1, Mn, Fe, Ni, Cu, Zn, As, Se, Cd, Ba, Hg, and Pb in *S. chamaejasme* were determined. There was no significant difference in the contents of 12 elements, but the contents of Mn, Zn, Ba, and Pb in *E. chamaejasme* were significantly higher than those in *E. lunata* (Yan et al., 2012)
*Talinum paniculatum* (Jacq.) Gaertn.	Traditional Chinese medicine	Metrorrhagia, hemorrhage	Antioxidant	—	The contents of Fe, Mn, Cu, Zn, Ba, Co, Ni, and Cd in *T. paniculatum* (Jacq.) Gaertn. were determined. The linearity of Mn, Cu, Zn, Ba, Co, and Fe is 0–500 μg/L, Ni and Cd are linear at 0–100 μg/L, the linear correlation coefficient is 0.9847–0.9999, and the detection limit is 0.0007–0.02 mg/kg. The RSD of reference materials is between 4.45% and 9.13% (Guo et al., 2013)
*Tetrastigma hemsleyanum* Diels & Gilg	Traditional Chinese medicine	Bronchitis, pneumonia, pharyngitis, hepatitis	Antibacterial, anti-inflammatory, anti-tumor, and anti-liver injury	Zhejiang, Guangxi, Guizhou, Fujian, and Yunnan provinces or autonomous regions of China	The contents of Pb, Cd, As, Hg, and Cu in *T. hemsleyanum* Diels & Gilg from different producing areas in Zhejiang, Guangxi, and Guizhou provinces were determined. Pb ≤ 4.1677, Cd ≤ 0.1946, As ≤ 0.4550, Hg ≤ 0.0424, and Cu ≤ 7.8925 mg/kg (Wang et al., 2020a)
*Trichosanthes kirilowii* Maxim.	Traditional Chinese medicine	Cough	Anti-inflammatory, antibacterial, antiviral, and hypoglycemic	Anhui province of China	The contents of 12 trace elements Pb, Cd, As, Hg, Cu, Mg, Cr, Mn, K, Se, Sr, and Fe in *T. kirilowii* Maxim. from different producing areas in the Anhui province of China were determined. The contents of Pb, Cd, As, Hg, Cu and other five heavy metals in trichosanthin from Bozhou and Anguo in Anhui province are lower than the values specified in the Chinese Pharmacopoeia. There were significant differences in the contents of K, Mn, Mg, Sr, and other elements in *T. kirilowii* Maxim. between the two producing areas (Gong et al., 2018)

a Note: the origin of medicinal materials recorded in the table is collected from the articles.

If the origin of medicinal materials is not recorded in the articles, it is indicated by “—.”

### Analysis and Application of ICP-MS in Medicinal Materials Derived From the Roots of Plants

Medicinal materials derived from the roots and rhizomes of plants account for the largest proportion and are the most common in the medicinal materials derived from plants, so ICP-MS is also the most widely used in this kind of traditional Chinese medicine. From the 40 articles on medicinal materials derived from the roots of plants, 64 elements such as Cu, As, Cd, Pb, Hg, Cr, and Mn were determined, of which 31 were the most, accounting for 77.50%. Of the 40 articles on medicinal materials derived from the roots of plants, 36 belong to traditional Chinese medicine, accounting for 90%. Three articles belong to Arabic medicine (including one article on Unani), accounting for 7.50%. One article belongs to Tibetan medicine, accounting for 3%. The application of ICP-MS in medicinal materials derived from the roots of plants is shown in Table 1.

### Analysis and Application of ICP-MS in Medicinal Materials Derived From the Stem of Plants

Medicinal materials derived from the stem of plants refer to the above-ground stem or part of the medicinal materials derived from the stem of plants. Most of them are the stems of woody plants, and a few are the stems and vines of herbaceous plants. In 28 articles on medicinal materials derived from the stem of plants, 61 elements such as Cu, Cd, As, Pb, Cr, Mn, and Ni were determined. Among them, the determination of Cu is the most, with 27 articles accounting for 96.43%. Of the 28 articles on the determination of medicinal materials derived from the stem of plants, 27 belong to traditional Chinese medicine, accounting for 96%. One article belongs to Arabic medicine, accounting for 4%. The application of ICP-MS in medicinal materials derived from the stem of plants is shown in Table 2.

**TABLE 2:** Application of ICP-MS in the determination of heavy metals in medicinal materials derived from the stem of plants.

Latin name	Medical system	Therapeutic use	Main pharmacological effects	Origin of medicinal materials^a^	Test results
*Acorus calamus* L.	Traditional Chinese medicine	Forgetfulness, tinnitus, deafness, traumatic injury	Antidepressant, anti-anxiety, antihypertensive, antibacterial, anti-tumor	Sichuan province of China	The contents of Mg, P, Ca, Cr, Mn, Fe, Ni, Cu, Zn, As, Cd, Hg, and Pb in *A. calamus* L. were determined. They are Fe-676, Mn-1275, Cu-13.3, and Zn-36.7 mg/kg, respectively. As is 1.1 mg/kg, the contents of Cr, Ni, and Pb are less than 0.5 mg/kg, and the contents of Cd and Hg are less than 0.05 mg/kg. The contents of major elements Ca, P, and Mg are relatively high, which are Ca-6101, p-3706, and Mg-130 mg/kg, respectively (Li, 2017)
*Allium victorialis* L.	Traditional Chinese medicine	Cardiopathy, hypertension, arteriosclerosis	Anti-inflammation, bacteriostasis, anti-neuroinflammation, prevention, and treatment of cardiovascular diseases	Dongfeng County, Jilin province, China	The contents of Li, Be, B, Sc, V, Cr, Mn, Fe, Co, Ni, Cu, Zn, Ga, Ge, As, Se, Rb, Sr, Zr, Nb, Mo, Ag, Cd, Sn, Sb, Te, Cs, Ba, Hf, Ta, W, Au, Tl, Pb, Bi, Th, and U in the stems and leaves of *A. victorialis* L. were determined. The mass concentration range of the measured elements is 0.008243–886.2 μg·L^−1^, *r* ≥ 0.9996, the detection limit is 0.0002–0.1240 μg·L^−1^, the recovery rate is 97.11%–100.30%, and RSD ≤ 5.0%. The stems and leaves of *A. victorialis* L. are rich in trace elements, among which the content of Fe is the highest, followed by Mn, Sr, Ba, Zn, and B. The contents of heavy metal elements Cu, As, Pb, and Cd are lower than the national standard limit (Si et al., 2020)
*Anemarrhena asphodeloides* Bunge	Traditional Chinese medicine	Cough, fever, constipation	Anti-tumor, hypoglycemic, improving Alzheimer’s disease	Hebei, Anhui, Inner Mongolia, Shanxi, Sichuan, and Jilin provinces or autonomous regions of China	The contents of Cu, As, Cd, Pb, and Hg in 20 batches of *A. asphodeloides* Bunge from six producing areas such as Hebei and Anhui were determined. One sample of heavy metal exceeding the standard is Cd, and the exceeding rate is 5.0% (Xie, 2018)
*Atractylodes macrocephala* Koidz.	Traditional Chinese medicine	Ulcers, constipation, insomnia, diabetes, hypertension, cancer	Anti-inflammation, bacteriostasis, improving gastrointestinal function and nerve function	Longhui, Pingjiang, and Longshan counties, Hunan province, China	The contents of 48 trace elements Li, Be, B, Sc, Ti, V, Cr, Mn, Co, Ni, Cu, Zn, Ga, Ge, As, Se, Rb, Sr, Y, Zr, Nb, Mo, Ag, Cd, Sn, Sb, Cs, Ba, La, Ce, Pr, Nd, Sm, Eu, Gd, Tb, Dy, Ho, Er, Tm, Yb, Lu, Ta, W, Hg, Tl, Pb, and Bi in three batches of rhizomes of *A. macrocephala* were determined. The linear relationship of each element is good, and the correlation coefficient of each standard curve is *r* ≥ 0.9996. The detection limit is 0.0001–0.1600 g/L. RSD of precision, stability, and repeatability were less than 4.7%, 4.9%, and 4.4%, respectively. The recovery was 96.9%–101.7% and RSD was 0.3%–3.5% (Yang et al., 2018)
*Bletilla striata* (Thunb.) Rchb.f.	Traditional Chinese medicine	Hemoptysis, hematemesis, traumatic bleeding, sore swelling, poison	Anti-inflammatory, anti-tumor, antiviral microorganism, mucosal protection	Chongqing and Hubei provinces or municipalities of China	The residues of Pb, Cd, As, Hg, and Cu in *B. striata* (Thunb.) Rchb.f. from different producing areas such as Hubei, Yunnan, Guizhou, and Sichuan provinces of China were determined. The linear relationship of the five elements is good (*r* > 0.9992), and the recovery is 99%–114% (Li et al., 2021)
*Conioselinum anthriscoides* (H. Boissieu) Pimenov & Kljuykov	Traditional Chinese medicine	Heartache, irregular menstruation, amenorrhea, dysmenorrhea, headache	Anti-tumor, anticoagulant, anti-cerebral ischemia, and anti-depression	Sichuan province of China	The contents of Cr, Mn, Co, Ni, Cu, As, Cd, Ba, Tl, and Pb in *C. anthriscoides* “Chuanxiong” were determined. When the sample weight is 0.2 g, the determination results are Cr (1.7262 ± 0.180) mg/kg, Mn (55.5089 ± 2.640) mg/kg, Co (0.2652 ± 0.012) mg/kg, Ni (1.1429 ± 0.065) mg/kg, Cu (9.8778 ± 0.400) mg/kg,. As (0.1297 ± 0.010) mg/kg, Cd (0.4061 ± 0.030) mg/kg, Ba (12.2330 ± 1.520) mg/kg, Tl (0.0236 ± 0.003) mg/kg, Pb (0.7091 ± 0.051) mg/kg (Zhang et al., 2017a)
*Coptis chinensis* Franch.	Traditional Chinese medicine	Hyperglycemia, hypertension	Anti-inflammation, anti-arrhythmia, lowering blood pressure and anti-tumor	—	The contents of 39 inorganic elements Mg, K, Cu, Mn, Fe, Ni, Zn, Rb, Sr, Ba, Ag, Pb, As, Cd, Cr, Li, be, V, Co, Ga, Se, Y, Cs, La, Ce, Pr, Nd, Sm, Eu, Gd, Tb, Dy, Ho, Er, Tm, Yb, Lu, Tl, and Th in 67 batches of *C. chinensis* Franch. were determined. The average contents of K, Mg, Mn, Zn, and Fe are the highest, and the content of rare earth elements is generally low (Zhou et al., 2021)
*Corydalis yanhusuo* (Y. H. Chou & Chun C. Hsu) W. T. Wang ex Z. Y. Su & C. Y. Wu	Traditional Chinese medicine	Chest, flank, epigastric pain, heartache, dysmenorrhea	Analgesia, anti-anxiety, anti-myocardial ischemia, anti-gastrointestinal ulcer	—	1) The contents of nine heavy metals and harmful elements such as Cu, Ni, Cr, Pb, Cd, Sn, Co, Hg, and As in *C. yanhusuo* (Y. H. Chou & Chun C. Hsu) W. T. Wang ex Z. Y. Su & C. Y. Wu were determined. The linear relationship between nine heavy metals and harmful elements is good (*r* ≥ 0.9998), the detection limit is 0.0016–0.0227 μg/g, and the recovery rate is 96.0%–100.4%, RSD ≤ 3.25%. The contents of Cu, As, Pb, Hg, and Cd in *C. yanhusuo* (Y. H. Chou & Chun C. Hsu) W. T. Wang ex Z. Y. Su & C. Y. Wu are lower than the limit values of the national standard green industry standard for the import and export of medicinal plants and preparations and the 2015 edition of Chinese Pharmacopoeia (Li et al., 2019a)
					2) The contents of Pb, Cr, Cd, Cu, and Mn in *C. yanhusuo* (Y. H. Chou & Chun C. Hsu) W. T. Wang ex Z. Y. Su & C. Y. Wu were determined. The calibration curves of the five elements have a good linear relationship, and the recovery of each element is between 98.9% and 101.3% (Su, 2012)
*Curcuma phaeocaulis* Valeton	Traditional Chinese medicine	Tumor, inflammation, thrombus	Anti-inflammatory, hypoglycemic, and antioxidant	Hainan, Guangdong, Yunnan, and Guangxi provinces or autonomous regions of China	The elements of Li, Hg, Be, Mg, Pb, K, V, Ca, Cu, Ti, As, Cr, Mn, Ba, Fe, Tl, Ni, Zn, Ga, Sb, Na, Sr, Cd, and Al in *C. phaeocaulis* Valeton from different habitats were determined. The contents of trace elements in *C. phaeocaulis* Valeton from different producing areas are different, and the contents of K, Ca, Mg, Mn, Fe, Al, Zn, and other elements are higher (Zhang et al., 2017b)
*Cymbopogon schoenanthus* (L.) Spreng.	Arabic medicine	Malaria, colds, fever, asthma,	Antibacterial and antioxidant	Saudi Arabia	The contents of Fe, Mn, Zn, Cu, and Se in *C. schoenanthus* (L.) Spreng. were determined. Fe level is the highest, and Se level is the lowest among all plants. The TE series levels of all elements in all plants are as follows: Fe 193.4–1757.9, Mn 23.6–143.7, Zn 15.4–32.7, Se 0.13–0.92, and Cu 11.3–21.8 μg/g (Brima, 2018)
*Dendrobium officinale* Kimura & Migo	Traditional Chinese medicine	Hyperglycemia, hypertension, liver injury, tumor	Enhance immunity, protect the liver, and improve gastrointestinal function	Anhui, Guizhou, Guangdong, Guangxi, Zhejiang, and Yunnan provinces or autonomous regions of China	1) The contents of Fe, Mn, Zn, Cr, Ni and V in *Dendrobium officinale* Kimura & Migo were determined. There was a positive correlation between Ni and Zn (*r* = 0.986, *p* < 0.01). Ti was positively correlated with V (*r* = 0.669, *p* < 0.05), and negatively correlated with Cr (*r* = −0.710, *p* < 0.05). There was a positive correlation between Mn and BA (*r* = 0.749, *p* < 0.05). Iron content was positively correlated with Ni (*r* = 0.664, *p* < 0.05), Zn (*r* = 0.742, *p* < 0.05), and rare earth (*r* = 0.847, *p* < 0.01) (Zhu et al., 2016)
					2) The contents of 24 inorganic elements of Li, Na, Mg, K, Ca, V, Cr, Mn, Fe, Co, Ni, Cu, Zn, Ga, As, Rb, Sr, Cd, Cs, Ba, La, Nd, Tl, and Pb in *D. officinale* Kimura & Migo from different origins in Anhui, Guangdong, Guangxi, Guizhou, and Yunnan were determined. The contents of K, Ca, Mg, and Na in Dendrobium candidum are high (Zhao et al., 2020a)
					3) The contents of Pb, Cd, Cu, As, and Hg in *D. officinale* Kimura & Migo were determined. The linear relationship of the five heavy metal elements is good, the correlation coefficient is 0.9982–0.9999, and the detection limit is 0.008–0.750 μg/L. The recovery was 90.4%–96.9%, and the relative standard deviation was 1.6%–8.2% (Jia et al., 2011)
*Dioscorea oppositifolia* L.	Traditional Chinese medicine	Hyperglycemia, hyperlipidemia	Hypoglycemic, hypolipidemic, antioxidant, and anti-tumor	Anhui, Henan, and Guizhou provinces of China	The contents of 17 elements of Na, Mg, K, Ca, Cr, Mn, Fe, Ni, Zn, Se, Sr, Mo, Pb, Cd, Hg, Cu, and As in *D. oppositifolia* L. were determined. The detection limit of each inorganic element is 0.00004–0.016 mg/kg, the linear relationship is good, the correlation coefficient is in the range of 0.9993–0.9999, the recovery rate of standard addition is 95%–105%, and the determination result is accurate (Long, 2018)
*Gastrodia elata* Blume	Traditional Chinese medicine	Infantile convulsion, headache, epilepsy, tetanus	Anticonvulsant, antidepressant, neuroprotective	Anhui, Yunnan, Shaanxi, Guizhou, Henan, Hubei, Sichuan, Jilin, Zhejiang, Heilongjiang, and Chongqing provinces or municipalities of China	The contents of As, Ba, Be, Cd, Co, Cr, Cu, Ga, Hg, Mg, Mn, Mo, Ni, Pb, Se, Sn, Sr, Ti, Tl, and V in 30 batches of *G. elata* Blume from different habitats were determined. The linear relationship of 20 elements to be tested is good (*R* ≥ 0.9982), the RSD of the repeatability test is ≤6.34%, the RSD of the stability test is ≤6.00%, the RSD of the precision test is ≤5.78%, the recovery of the sample is 91.62%–108.10%, and the RSD is ≤5.21% (Yu et al., 2017a).
*Iris domestica* (L.) Goldblatt & Mabb.	Traditional Chinese medicine	Cough, inflammation, tonsillitis, lumbago	Anti-inflammatory, antibacterial, and antiviral	Henan, Hebei, Hubei, Anhui, Hunan, Sichuan, and Guizhou provinces of China	The contents of Pb, Cd, As, Hg, and Cu in 13 batches of *I. domestica* (L.) Goldblatt & Mabb. harmful metals from seven different producing areas such as Henan, Hunan, and Sichuan provinces of China were determined. Among 13 batches of samples from seven producing areas, only four batches of samples have all elements within the limit, and other samples have different elements exceeding the standard, of which the exceeding standard rate of Cd element is As high as 62% (Guan et al., 2021)
*Melicope pteleifolia* (Champ. ex Benth.) T.G.Hartley	Traditional Chinese medicine	Pharyngalgia, malaria, icteric hepatitis, cold, fever, tonsillitis	Anti-inflammation and analgesia	—	The contents of Cu, Pb, Hg, As, and Cd in *M. pteleifolia* (Champ. ex Benth.) T. G. Hartley were determined. The correlation coefficient of the standard curve of the measured elements is in the range of 0.9996–0.9999, and the linear relationship is good. The average recovery of the method is 93.4%–96.6%, and the RSD is 4.6%–9.4% (Long et al., 2013).
*Pleione bulbocodioides* (Franch.) Rolfe	Traditional Chinese medicine	Snakebite	Anti-tumor	Guizhou province of China	The contents of Be, Ti, V, Cr, Mn, Co, Ni, Cu, Zn, As, Ag, Cd, Sn, Sb, and Ba in Guizhou *P. bulbocodioides* (Franch.) Rolfe were determined. The linear relationship of each element is good, and the correlation coefficient is greater than 0.995. The relative standard deviation (RSD) of repeatability is less than 7.5%. Relative standard deviation of precision (RSD) < 4%. The recovery was 81.37%–114.61% (Chen et al., 2020c)
*Polygonatum odoratum* (Mill.) Druce	Traditional Chinese medicine	Hyperglycemia, inflammation	Antioxidant, hypoglycemic, anti-tumor, antibacterial	—	11 elements such as Li, B, Cr, Co, Ni, Cu, Mo, Pb, Be, Cd, and Tl in *P. odoratum* (Mill.) Druce were determined. The content of K is the highest, reaching 8652 μg/g, Ca 2158 μg/g, Mg 696 μg/g, Al 444 μg/g, Fe 191 μg/g, Pb 0.71 μg/g, Cd 0.43 μg/g, Tl 0.063 μg/g. Be content is the least, only 0.028 μg/g (Liu et al., 2016)
*Sargentodoxa cuneata* (Oliv.) Rehder & E.H.Wilson	Traditional Chinese medicine	Amenorrhea, dysmenorrhea	Antibacterial, antiviral, anti-inflammatory, anti-tumor, immunosuppressive	Hunan, Anhui, Zhejiang, and Jiangxi provinces of China	Determination of 23 elements Na, Mg, Al, K, CA, V, Cr, Mn, Fe, Co, Ni, Cu, Zn, As, Se, Rb, Ag, Cd, Cs, Ba, Hg, and Pb in *S. cuneata* (Oliv.) Rehder & E. H. Wilson in Hunan, Anhui, and Zhejiang, China. For the measured elements, the correlation coefficient of the standard curve is *r* > 0.9987, and the relative standard deviation of the method accuracy experiment is RSD < 9.1% (Liu and Shu, 2013)
*Sauromatum giganteum* (Engl.) Cusimano & Hett.	Traditional Chinese medicine	Tetanus, epilepsy, snakebite	Anti-infection, anti-convulsion, anti-tumor	Henan, Jilin, Shaanxi, Hubei, and Sichuan provinces of China	The contents of Ni, Zn, As, Cd, Pb, Cr, Cu, Mn, Ba, Co, Sr, Se, Ti, Fe, Al, K, Mg, P, and Hg in *S. giganteum* (Engl.) Cusimano & Hett. were determined. The contents of K, P, Mg, Fe, Zn, Al, and Mn are high (Liu et al., 2018b).
*Sinomenium acutum* (Thunb.) Rehder & E. H. Wilson	Traditional Chinese medicine	Rheumatoid arthritis pain, chronic glomerulonephritis, arrhythmia	Anti-inflammatory, analgesic, and anti-tumor	Guangxi Zhuang autonomous region of China	The matrix effect was compensated with Be, Y, Sc, In, and Re as internal standards. The contents of 28 elements of Li, B, Na, Mg, Al, K, Ca, Ti, V, Cr, Mn, Fe, Co, Ni, Cu, Zn, As, Se, Sr, Mo, Ag, Cd, Sn, Sb, Ba, Tl, Pb, and Bi in *S. acutum* (Thunb.) Rehder & E. H. Wilson were determined. The detection limit of each element is 0.01–0.8 μg·L^−1^, the standard deviation is 0.11%–2.57%, and the recovery is 94.00%–110.80%. The content of 16 elements in *Sinomenium wilfordii* is 2 mg·kg^−1^, among which K, Mg, CA, Na, and other elements have the highest content (Duan et al., 2012b).
*Sparganium stoloniferum* (Buch.-Ham. ex Graebn.) Buch.-Ham. ex Juz.	Traditional Chinese medicine	Dysmenorrhea, amenorrhea, heartache	Anti-inflammation, analgesia, anti-tumor, anti-fibrosis, and inhibition of ovarian cyst	—	The contents of Mn, Cr, Sr, Zn, Rb, B, Ni, Cu, Mo, and Se in *S. stoloniferum* (Buch.-Ham. ex Graebn.) Buch.-Ham. ex Juz. were determined. The correlation coefficients of the regression equation of each element *r* ≥ 0.9998, the detection limit was 0.0019–0.1644 g/L, and the recovery was 96.33%–113.33%. The content of Mn, Zn, Ni, B, Cu, and other elements in traditional Chinese medicine Sanleng is high, and the content of Mn is the highest (681.5 g/g) (Wang and Jin, 2018d)
*Spatholobus suberectus* Dunn	Traditional Chinese medicine	Tumor, viral cold	Anti-inflammatory, analgesic, anti-tumor, antiviral, anti-depressive	Guangxi Zhuang autonomous region of China	The contents of 28 elements of Li, B, Na, Mg, Al, K, Ca, Ti, V, Cr, Mn, Fe, Co, Ni, Cu, Zn, As, Se, Sr, Mo, Ag, Cd, Sn, Sb, Ba, Tl, Pb, and Bi in *S. suberectus* Dunn were determined. The detection limit of each element is 0.01–0.8 μg/L, the standard deviation is 0.11%–2.57%, and the recovery rate is 94.00%–110.00%. There are 13 elements with a content of more than 2 mg/kg, among which the contents of Ca, K, Mg, and other elements are the highest (Su et al., 2013)
*Taxillus chinensis* (DC.) Danser	Traditional Chinese medicine	Rheumatism, metrorrhagia	Anti-tumor, lowering blood pressure, lowering blood sugar, lowering blood lipid	Guangxi Zhuang autonomous region of China	The contents of Pb, Cr, Hg, As, Cu, and Cd in *T. chinensis* (DC.) Danser were determined. The average contents of the six elements accord with Cu > As > Pb > Hg > Cr > Cd (Shen et al., 2017)
*Uncaria rhynchophylla* (Miq.) Miq.	Traditional Chinese medicine	Hypertension, arrhythmia, Alzheimer’s disease	Anti-inflammatory, analgesic, and anticancer	Guizhou province of China	The contents of K, Ca, Mg, Na, Fe, Mn, Cu, Zn, Mo, Co, Pb, Cr, Hg, As, and Cd in the mixed samples of hook and stem of *U. rhynchophylla* (Miq.) Miq. in Jianhe County, Guizhou province, China, were determined. The contents of trace elements Na, Mg, K, Ca, Mn, Co, and Zn are greater than those in the hook. The highest content of trace elements in hook and stem was K, followed by Ca. The content of heavy metal elements Cr, Cd, and Pb in the stem is slightly higher than that in the hook, the content of Hg in the hook is higher than that in the stem, some exceed the standard, and the content of Cu and As in the hook and stem is close (Zhang et al., 2016b)
*Zingiber officinale* Roscoe	Traditional Chinese medicine	Inflammation, arrhythmia, myocardial ischemia, tumor	Anti-inflammatory, analgesic, and bacteriostatic	—	The contents of Cu, Zn, Co, Mn, Cr, Se, Ni, Mo, V, and Sn in dried *Z. officinale* Roscoe were determined. The contents of Mn, Cu, and Zn are 328.2049, 57.4596, and 20.2833 g/g, respectively, and the contents of Sn, Ni, Cr, Se, V, Co, and Mo are 9.0956, 4.5861, 3.2654, 1.6022, 1.0364, 0.4992, and 0.1175 g/g, respectively. The regression equation coefficient of each element *r* ≥ 0.9998, the linear relationship of 10 elements is good in the range of 0–500 g/L, the detection limit of each element is less than 0.1460 g/L, the quantitative limit is less than 0.4867 g/L, and the recovery rate is 99.63%–101.46%. The RSD of precision, stability, and repeatability were less than 3% (Wu et al., 2019a)

a Note: the origin of medicinal materials recorded in the table is collected from the articles.

If the origin of medicinal materials is not recorded in the articles, it is indicated by “—.”

### Analysis and Application of ICP-MS in Medicinal Materials Derived From the Leaf of Plants

Medicinal materials derived from the leaf of plants are a kind of medicinal parts, mostly complete and grown dry leaves, less tender leaves, or leaves with some tender branches. In 11 articles on medicinal materials derived from the leaf of plants, 64 elements such as As, Cd, Pb, Mn, Cu, Hg, and Cr were determined. Among them, the determination of As and Cd elements was the most, with eight articles accounting for 72.73%. Of the 11 articles on the determination of medicinal materials derived from the leaf of plants, seven belong to traditional Chinese medicine, accounting for 64%. Four articles belong to Arabic medicine, accounting for 36%. The application of ICP-MS in medicinal materials derived from the leaf of plants is shown in Table 3.

**TABLE 3:** Application of ICP-MS in the determination of heavy metal elements in medicinal materials derived from the leaf of plants.

Latin name	Medical system	Therapeutic use	Main pharmacological effects	Origin of medicinal materials^a^	Test results
*Ambrosia artemisiifolia* L.	Arabic medicine	Respiratory and reproductive system related diseases	Anti-inflammatory, analgesic, antibacterial, antiviral, and anticoagulant	Saudi Arabia	The contents of Al, Pb, Cd, and As in *A. artemisiifolia* L. were determined. The recoveries of all measured elements were 86.1%–90.6%. The content of Al element is the highest, and the concentration range of Al is 156–1609 mg/kg. The content of Cd is the lowest, and the concentration of Cd is in the range of 0.01–0.10 mg/kg. The washing process reduces toxic elements in all plants. The average recoveries were Al (47.32%), As (59.1%), Cd (62.03%), and Pb (32.40%) (Brima, 2017)
*Artemisia vulgaris* L.	Arabic medicine	Respiratory and reproductive diseases	Anti-inflammatory and antibacterial	Saudi Arabia	The contents of Fe, Mn, Zn, Cu, and Se in *A. vulgaris* L. were determined. Fe level is the highest, and Se is the lowest. Te series level: Fe 193.4–1757.9 μg/g, Mn 23.6–143.7 μg/g, Zn 15.4–32.7 μg/g, Se 0.13–0.92 μg/g, and Cu 11.3–21.8 μg/g (Brima, 2018)
*Callicarpa nudiflora* Hook. & Arn.	Traditional Chinese medicine	Acute infectious hepatitis, traumatic bleeding, respiratory bleeding, gastrointestinal bleeding	Antibacterial, anti-inflammatory, antiviral, and anti-tumor	—	The contents of Cr, Pb, Cd, Hg, As, and Ni in the dry extract of naked flower *C. nudiflora* Hook. & Arn. were determined. The linear range of six heavy metal elements is 0–40 ng/ml, and the correlation coefficient is 0.9982–0.9999. LOQ is 0.0049–0.0659 ng/ml. The precision, repeatability, and stability are good, and the relative standard deviation RSD is less than 2.0%. The recoveries of low, medium, and high levels were between 90.0% and 101.6% (Zheng et al., 2017)
*Crataegus pinnatifida* Bunge	Traditional Chinese medicine	Heartache, palpitation, hyperlipidemia	Anti-atherosclerotic, hypoglycemic, and liver-protective	Liaoning province of China	The contents of Mg, K, Ca, B, Na, Al, Si, Fe, Sr, Cr, Mn, Cu, Zn, Ga, Rb, Ba, Pb, Li, V, Co, As, Ru, In, Tm, Ta, Re, Os, Ir, Pt, Au, Hg, Bi, Be, Ge, Rh, Ag, Ho, Er, Ti, Th, Dy, U, Nb, Cs, Pr, Sm, Gd, Se, Zr, Mo, Sn, W, Ce, Pd, Cd, Sb, and Nd in leaves of *C. pinnatifida* Bunge were determined. The contents of K, Ca, Mg, Si, Fe, Al, Na, Sr, and B are higher, with the highest content of K and the lowest content of Tm (Zhang et al., 2012a)
*Cycas revolute* Thunb.	Traditional Chinese medicine	Amenorrhea, dystocia, cough, hematemesis, cancer	Anticancer	Guangxi, Jiangsu, Inner Mongolia, Anhui, Guangdong, and Yunnan provinces, municipalities or autonomous regions of China	The contents of Pb, Cd, As, Hg, and Cu in leaves of *C. revolute* Thunb. were determined. The five heavy metal elements have a good linear relationship in the corresponding concentration range (*r* ≥ 0.999). The precision of the method meets the requirements. The recovery rate of standard addition is 87.4%–121.9%, and the RSD is less than 5% (Chen et al., 2020a)
*Laminaria japonic*a Aresch.	Traditional Chinese medicine	Thyromegaly, cervical lymphadenopathy, bronchitis, tuberculosis, cough, senile cataract	Antibacterial, antiviral, hypoglycemic, and anti-tumor	—	The contents of 15 rare earth elements La, Ce, Pr, Nd, Sm, Eu, Gd, Tb, Dy, Ho, Er, Tm, Yb, Lu, and Y in *L. japonica* Aresch. were determined. The detection limit of 15 rare earth elements is 14.69–31.25 ng/l, the method precision (RSD) is in the range of 1.4%–6.7%, and the recovery is in the range of 93.8%–108.6%. The contents of Y, La, Ce, and Nd in *L. japonica* Aresch. are high (Lei, 2017)
*Morus alba* L.	Traditional Chinese medicine	Hyperglycemia, cancer, viral cold	Hypoglycemic, hypolipidemic, antiviral, and anti-tumor	Guangdong province of China	The contents of Cr, Mn, As, and Cd in leaves of *M. alba* L. were determined. *r* is between 0.998124 and 0.999998, the recovery is 87%–100%, and the relative standard deviation (RSD) is less than 2% (Shen et al., 2013)
*Platycladus orientalis* (L.) Franco	Traditional Chinese medicine	Hematemesis, epistaxis, hemoptysis, hematochezia	Antibacterial, anti-inflammatory, anti-tumor, antioxidant, and hypolipidemic	Shanxi, Hunan, Hainan, Hebei, Chongqing, Gansu, Henan, and Anhui provinces or municipalities of China	The contents of 18 heavy metals and trace elements Pb, Ti, Hg, Dy, Ba, Sb, Sn, Cd, Ag, Mo, Se, As, Cu, Ni, Mn, Cr, Al, Be, Ge, and In in 63 batches of leaves of *P. orientalis* (L.) Franco from different producing areas such as Hunan, Shanxi, and Hainan provinces of China were determined. The linear relationship of 18 elements was good, the correlation coefficient was *R* ^2^ ≥ 0.9746, and the detection limit of each element was 0.224–1.792 μg·L^−1^, the recovery was 81.0%–117.1%. 63 batches of *P. orientalis* (L.) Franco leaf samples, the contents of Hg, Cu, Cd, and Sb were low. As was not detected, some samples of Ba exceeded the limit, most samples of Pb exceeded the limit, and Al exceeded the limit (Zhang et al., 2018)
*Psidium guajava* L.	Traditional Chinese medicine	Dysentery, acute enteritis, chronic enteritis	Hypoglycemic, hypolipidemic, and antiviral	Guizhou, Guangxi, Guangdong, Yunnan, Jiangxi, Hainan, and Fujian provinces or autonomous regions of China	The contents of Be, Ti, V, Cr, Mn, Co, Ni, Cu, As, Sr, Mo, Cd, Sb, Pb, and Hg in leaves of *P. guajava* L. were determined. The linear relationship of each element is good, and the correlation coefficient is greater than 0.995. The RSD of each element is less than 4.57%, the RSD of sample repeatability is less than 12.09%, and the recovery range of each element is 76.96%–118.55% (Li et al., 2019b)
*Vitex agnus-castus* L.	Arabic medicine	Irregular menstruation	Anti-inflammatory, antibacterial, anticancer, and antioxidant	Saudi Arabia	1) The contents of Fe, Mn, Zn, Cu, and Se in *V. agnus-castus* L. were determined. Fe level is the highest, and Se level is the lowest among all plants. The TE series levels of all elements in all plants are as follows: Fe 193.4–1757.9 μg/g, Mn 23.6–143.7 μg/g, Zn 15.4–32.7 μg/g, Se 0.13–0.92 μg/g, and Cu 11.3–21.8 μg/g (Brima, 2018)
					2) The contents of Al, Pb, Cd, and As in *V. agnus-castus* L. were determined. The recoveries of all measured elements were 86.1%–90.6%. The content of Al element is the highest, and the concentration range of Al is 156–1609 mg/kg. The content of Cd is the lowest, and the concentration of Cd is in the range of 0.01–0.10 mg/kg. The washing process reduces toxic elements in all plants. The average % recoveries were Al (47.32%), As (59.1%), Cd (62.03%), and Pb (32.40%) (Brima, 2017)

a Note: the origin of medicinal materials recorded in the table is collected from the articles.

If the origin of medicinal materials is not recorded in the articles, it is indicated by “—.”

### Analysis and Application of ICP-MS in Medicinal Materials Derived From the Flowers of Plants

Medicinal materials derived from the flowers of plants generally refer to a complete flower, inflorescence, or a part of a flower. Among the 12 articles on medicinal materials derived from the flowers of plants, 38 elements such as Cu, As, Cd, Pb, Ni, Hg, and Cr were determined. Among them, elements Cu, As, Cd, and Pb were determined most, with 12 articles accounting for 100%. Of the 12 articles on the determination of lower medicinal materials from plants, 11 belong to traditional Chinese medicine, accounting for 92%. One article belongs to Tibetan medicine, accounting for 8%. The application of ICP-MS in medicinal materials derived from the flowers of plants is shown in Table 4.

**TABLE 4:** Application of ICP-MS in the determination of heavy metal elements in medicinal materials derived from the flowers of plants.

Latin name	Medical system	Therapeutic use	Main pharmacological effects	Origin of medicinal materials^a^	Test results
*Carthamus tinctorius* L.	Traditional Chinese medicine	Amenorrhea, dysmenorrhea, tumbling injury	Anti-inflammation, anti-aging, anti-tumor, and anti-arrhythmia	Xinjiang, Henan provinces or autonomous regions of China	1) The contents of various elements in *C. tinctorius* L. in Xinjiang and Henan provinces of China were determined. 19 elements in safflower include heavy metals As, Cd, Cu, Hg, Pb, and healthy elements Al, Ca, Co, Cr, Fe, Mg, Mn, Mo, Ni, P, Se, Sr, V, and Zn. The contents of heavy metals in the samples are low. Except for Pb in Xinjiang samples, the rest meet the national health standards (Jia et al., 2011)
					2) The contents of 22 trace elements Li, Be, V, Cr, Mn, Co, Ni, Cu, Zn, Ga, As, Sr, Mo, Pb, Ag, Cd, In, Cs, Ba, Hg, Tl, and Bi in *C. tinctorius* L. were determined. The correlation coefficients of regression equations of all elements were >0.99. The recovery was 85.9%–107.2%. Except for Ni, Zn, and Ag, the content of the other 19 elements in *C. tinctorius* L. is high (Yin et al., 2014)
*Crocus sativus* L.	Tibetan medicine	Amenorrhea, symptoms, depression, stuffiness, palpitation, and madness	Anti-tumor and immune regulation	—	The contents of 22 trace elements Li, Be, V, Cr, Mn, Co, Ni, Cu, Zn, Ga, As, Sr, Mo, Pb, Ag, Cd, In, Cs, Ba, Hg, Tl, and Bi in *C. sativus* L. were determined. Each element *r* > 0.99. The recovery was 85.9%–107.2%. The contents of Ni, Zn, and Ag in *C. sativus* L. were high (Yin et al., 2014)
*Chrysanthemum morifolium* (Ramat.) Hemsl.	Traditional Chinese medicine	Cold, intestinal carbuncle	Anti-inflammatory, antibacterial, anti-aging, and antioxidant	Jiangsu, Zhejiang, and Hubei provinces of China	1) The contents of Pb, Cd, As, Hg, and Cu in *C. morifolium* (Ramat.) Hemsl. from Jiangsu, Zhejiang, and Hubei provinces of China were determined. The RSD of precision, repeatability, and stability of the five elements is less than 3.0%, and the recovery rate (*n* = 6) is 87.9%–105.8% (Deng et al., 2020)
					2) The contents of 16 elements Pb, Cd, As, Hg, Cu, Sb, Sn, Cr, Ni, Ba, Mn, Tl, Ag, Be, Dy, and Al in *C. morifolium* (Ramat.) Hemsl. were determined. The linear relationship of the standard curves of 16 elements is good. The linear correlation coefficient of all elements is *r* > 0.9995, the detection limit is 0.002–0.455 g·kg^−1^, the recovery of each element is 84%–107%, and the RSD < 5% (Xi et al., 2012)
*Chrysanthemum indicum* L.	Traditional Chinese medicine	Eye pain, headache, dizziness	Antibacterial, antiviral, anti-tumor, hypotensive, and hypolipidemic	Hubei, Henan, and Anhui provinces of China	The contents of 18 elements As, B, Ba, Cd, Co, Cr, Cu, Ga, Hg, Li, Mo, Ni, Sb, Se, Pb, Rb, Sr, and Zn in *C. indicum* L. in Hubei, Henan, and Anhui provinces of China were determined. The contents of inorganic elements of *C. indicum* L. in different provinces were different (Chen et al., 2020d)
*Leucaena leucocephala* (Lam.) de Wit	Traditional Chinese medicine	Diabetes	Bacteriostasis	Yunnan province of China	The contents of 27 metal elements such as Li, Be, B, Mg, Al, Co, Ni, Ga, Rb, Sr, Te, Ba, Bi, U, V, Cr, Mn, Fe, Cu, Zn, As, Se, Mo, Ag, Cd, Tl, and Pb in the seed and seed coat of *L. leucocephala* (Lam.) de Wit produced in Jianshui County, Yunnan provinces of China were determined. Eight metal elements such as Co, Ni, Cr, Zn, Se, Mo, Ag, and Pb were not detected. The detection limits of the method were Fe 9.789, Cr 2.691, Zn 1.803, B 2.076, Mg 1.977, Al 3.024, Ni 1.824 ng/ml, and other elements 0.003–0.921; RSD of seed precision 0.141%–11.86%, RSD of seed coat precision 0.044%–31.14%, and recovery between 90.8% and 107.1% (Zhang et al., 2012b).
*Lonicera hypoglauca* Miq.	Traditional Chinese medicine	Throat obstruction, erysipelas	Antiviral microorganism, anti-inflammatory, antioxidant and anti-tumor	Shandong province of China	The contents of Li, Be, Ga, V, Cr, Mn, Co, Ni, Cu, As, Sr, Mo, Cd, Sn, Sb, Ba, Hg, Tl, Pb, and Bi in *L. hypoglauca* Miq. and *Lonicera confusa* DC. were determined. The linear relationship of each element is good. The detection limit of the method is 0.004–0.071 μg·L^−1^. Good precision and accuracy. The recovery is in the range of 80%–100% (Xu and Liu, 2015)
*Lonicera japonica* Thunb.	Traditional Chinese medicine	Heatstroke, throat obstruction, various infectious diseases	Anti-inflammatory, antibacterial, antiviral, hypolipidemic, and hypoglycemic	Shandong, Henan, Hebei, Chongqing, Hubei provinces or municipalities of China	1) The contents of 27 metal elements Li, Be, B, Mg, Al, Co, Ni, Ga, Rb, Sr, Te, Ba, Bi, U, V, Cr, Mn, Fe, Cu, Zn, As, Se, Mo, Ag, Cd, Tl, and Pb in *L. japonica* Thunb. produced in Hubei province of China were determined. The detection limits of the method were 9.789 ng/ml for Fe, 2.691 ng/ml for Cr, 1.803 ng/ml for Zn, 2.076 ng/ml for B, 1.977 ng/ml for Mg, 3.024 ng/ml for Al, 1.824 ng/ml for Ni, 0.003–0.921 ng/ml for other elements, 0.146%–7.627% for precision, and 90.0%–110.0% for recovery (Shu et al., 2012)
					2) The contents of Pb, Cd, Cr, As, Hg, Ni, Mn, Cu, and Zn in *L. japonica* Thunb. were determined. The content of Cd was the highest (40.2%), followed by Cu (37.6%) and Pb (8.5%). As and Hg do not exceed the standard value, and Cr, Ni, Mn, and Zn are not limited (Fan et al., 2020)
*Wikstroemia chamaedaphne* (Bunge) Meisn.	Traditional Chinese medicine	Acute infectious hepatitis, chronic infectious hepatitis, schizophrenia, epilepsy	Anti hepatitis B virus	Shanxi and Anhui provinces of China	The contents of harmful elements Cu, Cd, Pb, As, and Hg in *W. chamaedaphne* (Bunge) Meisn. were determined. Cu, Pb, Cd, and As in 0.5–100 μg/L, Hg in 0.1–10 μg/L showed a good linear relationship, *r* ≥ 0.99, and the recovery was 95.24%–101.16% (Li et al., 2020a)
*Zea mays* L.	Traditional Chinese medicine	Jaundice, cholecystitis, hypertension	Anti-tumor, hypoglycemic, antibacterial, antioxidant	Shandong, Anhui, Jiangsu, and Henan provinces of China	The contents of inorganic elements such as B, Na, Mg, Al, K, Ca, Ti, V, Cr, Mn, Fe, Co, Ni, Cu, Zn, As, Se, Sr, Mo, Cd, Sn, Ti, Sb, Ba, Hg, Tl, and Pb in *Zea mays* L. were determined. The content of the K element is the highest, 16,436.57–19,599.22 mg/kg. The content of Mn in trace elements is the highest, 27.22–36.19 mg/kg (Chen et al., 2020b)

a Note: the origin of medicinal materials recorded in the table is collected from the articles.

If the origin of medicinal materials is not recorded in the articles, it is indicated by “—.”

### Analysis and Application of ICP-MS in Medicinal Materials Derived From the Fruits and Seeds of Plants

Most of the medicinal materials derived from the fruits and seeds of plants are complete, mature fruits and seeds. Among the 23 articles of medicinal materials derived from the fruits and seeds of plants, 65 elements such as Cu, Pb, Cd, Fe, Zn, As, and Al were determined, of which 19 were the most, accounting for 82.61%. Among the 23 articles on the determination of medicinal materials derived from the fruits and seeds of plants, 22 belong to traditional Chinese medicine, accounting for 96%. One article belongs to Arabic medicine, accounting for 4%. The application of ICP-MS in medicinal materials derived from the fruits and seeds of plants is shown in Table 5.

**TABLE 5:** Application of ICP-MS in the determination of heavy metal elements in medicinal materials derived from the fruits and seeds of plants.

Latin name	Medical system	Therapeutic use	Main pharmacological effects	Origin of medicinal materials^a^	Test results
*Alpinia oxyphylla* Miq.	Traditional Chinese medicine	Senile dementia and other mental disorders	Antibacterial, antioxidant stress, anti-tumor	Hainan province of China	The contents of Mg, Al, Fe, Zn, Cd, and Pb in *A. oxyphylla* Miq. were determined. Mg content is high 6550 μg/g, and Pb content is very low, only 1.59 μg/g (Chen et al., 2011b)
*Arctium lappa* L.	Traditional Chinese medicine	Tumor, hyperglycemia, inflammation	Anti-inflammatory, anti-tumor, antiviral, and hypoglycemic	Shandong, Jiangsu, Hebei, Anhui, Jilin, Sichuan, Shaanxi, Hunan, Gansu, Guangdong, and Hubei provinces of China	The contents of K, P, Mg, Al, Na, Fe, Pb, Hg, As, Cd, Cu, Si, Mn, Ni, Zn, Sr, V, Ti, Mo, Co, Sn, B, Li, and Cr in fried *A. lappa* L. were determined. The contents of K, P, Mg, Al, Na, and Fe are high, and the contents of heavy metals and harmful elements are within the limit standard (Hu et al., 2018)
*Citrus aurantium* L.	Traditional Chinese medicine	Gastroptosis, anal prolapse, uterine prolapse	Reduce blood lipid, blood pressure, antibacterial, and antioxidant	Quzhou, Zhejiang province, China	The contents of Mg, K, Ca, Na, Al, V, Cr, Mn, Fe, Co, Ni, Cu, Zn, Ga, As, Se, Rb, Sr, Ag, Cd, Cs, Ba, Tl, Pb, U, and Hg in *C. aurantium* L. were determined. The linear relationship of each element is good (*R* ≥ 0.9990), the average recovery is 90%–111%, and the RSD < 5%. The RSD of the precision, repeatability, and stability test meets the quantitative analysis requirements, and the detection limit is 0.0007–7.2264 μg·L^−l^. The contents of Mg, K, Ca, Na, Al, Mn, Fe, Cu, Zn, Rb, Sr, and Ba in the six batches of samples measured were high, and the contents of V, Cr, Co, Ni, Ga, As, Cd, Cs, Tl, Pb, Hg, Se, Ag, and U were low or not detected (Zheng et al., 2020)
*Citrus deliciosa* Ten.	Traditional Chinese medicine	Vomiting, diarrhea, cough	Anti-liver injury, anti-tumor, and anti-pulmonary fibrosis	—	The contents of Pb, As, Cd, Cu, and Hg in 20 batches of *C. deliciosa* Ten. were determined. The relative RSD of each element was 0.6%, 0.5%, 1.8%, 1.0%, and 0.6%, respectively (Xu et al., 2017)
*Cnidium monnieri* (L.) Cusson	Traditional Chinese medicine	Impotence, trichomonal vaginitis	Anti-arrhythmia, anti-tumor, and anti-myocardial fibrosis	—	The contents of 32 trace elements B, Na, Al, Mg, P, K, Ca, Sc, V, Cr, Mn, Fe, Co, Ni, Cu, Tb, Zn, Ga, Ge, As, Se, Sr, Mo, Cd, In, Ba, La, Ce, Hg, Pb, Bi, and Th in *C. monnieri* (L.) Cusson were determined. Among the major elements, the content of the K element is as high as 23.1 mg·g^−l^, followed by Ca, P, Mg, A1, and Na. Among the necessary trace elements, the content of Fe is the highest, up to 1161.7501 xg·g^−1^, followed by Mn, Zn, Cr, Cu, and Ni (Zhang et al., 2010)
*Cornus officinalis* Siebold & Zucc.	Traditional Chinese medicine	Impotence, spermatorrhea, enuresis, frequent urination	Anti-tumor, hypoglycemic, antioxidant, and anti-aging	Zhejiang, Shaanxi, and Henan provinces of China	The contents of 22 elements Li, Co, Na, Ni, Mg, Cu, Al, Zn, P, As, K, Se, Ca, Sr, Ti, Cd, Cr, Ba, Mn, Hg, Fe, and Pb in *C. officinalis* Siebold & Zucc. from different places in Zhejiang, Shaanxi, and Henan of China were determined. K. Ca, Mg, P, Na, Fe, Al, Zn, Cr, Mn, Ba, Sr, and other elements are abundant (Jiao et al., 2019)
*Euryale ferox* Salisb.	Traditional Chinese medicine	Spermatorrhea, enuresis, frequent urination	Anti-cancer, anti-myocardial ischemia, anti-fatigue, hypoglycemic	Fujian, Hunan, Guangdong, Jiangxi, Jiangsu, Anhui, Sichuan, Guizhou, Guangxi, Hebei, Liaoning, Shandong, and Yunnan provinces of China	The contents of Pb, Cd, Cr, Cu, Hg, and As in *E. ferox* Salisb. from different producing areas were determined. The linear correlation coefficients of the six elements were greater than 0.9971, the average recovery was 81.05%–97.97%, and the RSD was less than 5.0% (Zhang et al., 2019a)
*Foeniculum vulgare* Mill.	Traditional Chinese medicine	Diarrhea, cancer	Anti-inflammatory, antibacterial, and anti-fibrosis	—	The contents of Li, Be, B, Na, Mg, Al, Si, K, Ca, Sc, Ti, V, Cr, Mn, Fe, Co, Ni, Cu, Zn, Ga, Ge, As, Se, Br, Rb, Sr, Y, Zr, Nb, Mo, Rh, Pd, Ag, Cd, In, Sn, Sb, I, Cs, Ba, La, Ce, Pr, Nd, Sm, Eu, Gd, Tb, Dy, Ho, Er, Tm, Yb, Lu, Hf, W, Hg, Tl, Pb, Bi, Th, U, and Re in *F. vulgare* Mill. were determined. The contents of Na, Mg, Ca, Fe, Zn, Mn, Ba, and other metal elements in *F. vulgare* Mill. were high (Sun et al., 2013)
*Hovenia acerba* Lindl.	Traditional Chinese medicine	Liver disease, fatigue	Anti-aging and anti-fatigue	Guizhou province of China	The contents of Be, Mg, Al, Ti, V, Cr, Mn, Fe, Co, Ni, Cu, Zn, As, Sr, Mo, Ag, Cd, Sn, Sb, Ba, Tl, and Pb in the stem and fruit of *H. acerba* Lindl. were determined. The linear relationship of 22 elements is good in its linear range, and *r* > 0.995. The detection limit is 0.0007–1.0713 μg/L. RSD of repeatability is less than 3%. RSD of precision is less than 7%. The recovery was 81.14%–112.91% (Chen et al., 2020e)
*Lycium barbarum* L.	Traditional Chinese medicine	Hyperglycemia, hypertension	Antioxidant, anti-aging, hypoglycemic, and hypolipidemic	Ningxia, Xinjiang, and Qinghai provinces or autonomous regions of China	1) The average contents of elements in *L. barbarum* L. from Ningxia, Xinjiang, and Qinghai provinces of China were Pb 0.30 mg·kg^−l^, Cd 0.066 mg·kg^−l^, As 0.05 mg·kg^−l^, Hg 0.003 mg·kg^−l^, and Cu 0.71 mg·kg^−l^, respectively (Zuo et al., 2021)
					2) The contents of Pb, Cd, As, Hg, and Cu in 32 samples of *L. barbarum* L. were determined. No Pb element was detected in 19 samples, and the element contents of other samples were Pb ≤ 4.2 mg/kg, Cd ≤ 0.05 mg/kg, As ≤ 0.05 mg/kg, Hg ≤ 0.05 mg/kg, and Cu ≤ 7.10 mg/kg (Jiang et al., 2018)
*Moringa oleifera* Lam.	Traditional Chinese medicine	Tumor, hyperglycemia, hyperlipidemia	Antioxidant, anti-tumor, and anti-inflammatory	Yunnan Province of China	Determination of 22 inorganic elements of Na, Mg, Al, K, Ca, Cr, Mn, Fe, Co, Ni, Cu, Zn, As, Se, Mo, Ag, Cd, Sb, Ba, Hg, Tl, and Pb in seeds of *M. oleifera* Lam. from different habitats in Honghe, Chuxiong, Dehong, Zhaotong, Kunming, Yuxi, and Lijiang, Yunnan, China. The linear relationship of each element is good (*r* > 0.999). Detection limit: 0.04–78.44 μg/L. The precision meets the requirements (RSD < 5%). The recovery of each element is less than 10.66%–113% (RSD < 10%) (Niu et al., 2021)
*Myristica fragrans* Houtt.	Traditional Chinese medicine	Gastrosis, vomiting	Antiepileptic, antidepressant, anti-inflammatory, and anticancer	—	The contents of Mg, K, Ca, Fe, Na, Cu, Zn, Se, Cr, Co, Mn, V, Ni, Al, Sb, Ba, Bi, Li, and Sr in *M. fragrans* Houtt. were determined. The content of Mn was the highest, followed by Cu and Zn. After processing, the contents of Ca, Fe, Na, and other elements increased, whereas mg and K decreased. The contents of Al, Sb, Be, and other potentially toxic elements in nutmeg are low and basically unchanged after processing (Zhao and Jia, 2012)
*Nelumbo nucifera* Gaertn.	Traditional Chinese medicine	Diarrhea, spermatorrhea, insomnia	Reducing blood lipid, antioxidation, anti-inflammatory, and antithrombotic	—	The contents of As, Cd, Pb, and Hg in *N. nucifera* Gaertn. were determined. The linear relationship between the determined elements is good (*r* > 0.9995), and the recovery is 97.5%–107.8% (Chen et al., 2013)
*Prunella vulgaris* L.	Traditional Chinese medicine	Eye disease, headache	Anti-inflammatory, antihypertensive, hypoglycemic	—	The contents of Mg, Al, Ca, Mn, Fe, Zn, Se, Sn, Mo, and other metal elements in *P. vulgaris* L. are high (Cao et al., 2013)
*Prunus mume* (Siebold) Siebold & Zucc.	Traditional Chinese medicine	Cough, diarrhea, abdominal pain	Antibacterial, antioxidant, anti-tumor, hypoglycemic	Fujian, Guangdong, Zhejiang, Anhui, Guangxi, Sichuan, and Yunnan provinces or autonomous regions of China	The contents of 16 inorganic elements of B, Na, Al, Cr, Fe, Cu, Zn, Se, Sb, K, Ca, Mg, As, Hg, and Pb in *P. mume* (Siebold) Siebold & Zucc. were determined. Among the 16 elements, K, Ca, Mg, Na, Fe, and B were abundant (Ou et al., 2020)
*Prunus sibirica* L.	Traditional Chinese medicine	Cough, constipation	Anti-inflammatory, analgesic, anti-tumor, hypoglycemic	—	The contents of 24 trace elements B, Na, Mg, Al, P, K, Ca, Ti, V, Cr, Mn, Fe, Co, Ni, Cu, Zn, As, Se, Rb, Sr, Mo, Cd, Ba, and Pb in *P. sibirica* L. were determined. The relative standard deviation RSD of the measured elements is less than 4.79%, and the recovery is between 90.00% and 109.30% (Liu et al., 2013)
*Pseudocydonia sinensis* (Dum.Cours.) C. K. Schneid.	Traditional Chinese medicine	Spasmodic pain	Anti-inflammatory, analgesic, anti-gastric ulcer, anti-tumor	—	26 trace elements Na, Si, K, P, Zn, Sr, Mg, Cr, Mn, Co, Fe, Ni, I, Cu, Li, Al, Ca, Se, Rb, Cd, Be, Hg, B, Pb, C, and As in *P. sinensis* (Dum.Cours.) C. K. Schneid. were determined. The contents of K, Na, Ca, Mg, and other elements are relatively rich, the recovery is 96.5%–105.6%, and the RSD value is 0.26%–2.03% (Zhu, 2009)
*Schisandra chinensis* (Turcz.) Baill.	Traditional Chinese medicine	Cough, insomnia	Anti-inflammatory, anti-tumor, hypoglycemic, and hypolipidemic	Jilin Province of China	The contents of Al, As, Ba, Cd, Cr, Co, Cu, Fe, K, Mn, Ni, Pb, Se, V, and Zn in wild and cultivated *S. chinensis* (Turcz.) Baill. in Changbai Mountain area were determined. The detection limit of the method is 0.002–0.092 ng/ml, the relative standard deviation RSD is 1.87%–4.96%, and the recovery of standard addition is 94.0%–104.5% (Li et al., 2011)
*Senna Tora* (L.) Roxb.	Traditional Chinese medicine	Nyctalopia, constipation, oral ulcer	Reduce blood lipid, blood pressure, antibacterial and antioxidant	Guangxi Zhuang autonomous region of China	The contents of Na, Mg, P, Ca, Ti, Zn, Mn, Fe, K, Sr, B, Al, Ba, Li, Be, Tl, Mo, Pb, Cd, Co, V, Cr, Cu, and Ni in *S. tora* (L.) Roxb. were determined. *S. tora* (L.) Roxb. is rich in K, Ca, P, Mg, Al, Fe, and Na, whereas the contents of Be, Cd, Tl, Pb, and other elements are very small, less than 0.02 μg/g. Among the 24 elements determined, the content order is K > Ca > P > Mg > Al > Fe > Na > Zn > Sr > B > Mn > Ba > Cu > Ti > Cr > Ni > Mo > Co > V > Li > Cd > Pb > Tl > Be (Liu et al., 2012)
*Vitex agnus-castus* L.	Arabic medicine	Irregular menstruation	Anti-inflammatory, antibacterial, anticancer, and antioxidant	Saudi Arabia	The contents of Fe, Mn, Zn, Cu, and Se in *V. agnus-castus* L. were determined. Fe level is the highest, and Se level is the lowest among all plants. The TE series levels of all elements in all plants are as follows: Fe 193.4–1757.9 μg/g, Mn 23.6–143.7 μg/g, Zn 15.4–32.7 μg/g, Se 0.13–0.92 μg/g, and Cu 11.3–21.8 μg/g (Brima, 2018)
*Xanthium strumarium* L.	Traditional Chinese medicine	Rhinallergosis	Anti- inflammatory, analgesic, antibacterial, antiviral, anti-tumor, hypoglycemic	Sichuan, Hainan, Henan, Jilin, Xinjiang, and Yunnan provinces or autonomous regions of China	The contents of Pb, Hg, As, Cu, and Cd in *X. strumarium* L. were determined. The linear relationship of the five elements to be tested is good (*r* ≥ 0.9975), the recovery is 97.47%–104.24%, RSD ≤ 2.13%), and the detection limit of the method is 0.014–9.807 μg·L^−1^ (Liang et al., 2019)
*Ziziphus jujuba* Mill.	Traditional Chinese medicine	Hypertension, hyperlipidemia, arrhythmia	Anticonvulsant, anti-anxiety, antidepressant, anti-inflammatory, and hypotensive	Hebei, Henan, Shandong, Shaanxi, and Liaoning provinces of China	The contents of eight trace elements Mg, Mn, Fe, Zn, Cu, Cd, Hg, and Pb in *Z. jujuba* Mill. from 13 different producing areas in Hebei, Henan, Shaanxi, and Liaoning provinces of China were determined. The contents of Mg, Mn, Fe, and Zn, which are beneficial to the human body, are generally rich in j *Z. jujuba* Mill. from different producing areas. The contents of harmful heavy metal elements Cu, Cd, Hg, and Pb are within the limit standard (Liu et al., 2018c)

a Note: the origin of medicinal materials recorded in the table is collected from the articles.

If the origin of medicinal materials is not recorded in the articles, it is indicated by “—.”

### Analysis and Application of ICP-MS in Medicinal Materials From the Whole Plants

Medicinal materials from the whole plants refer to the whole plant or its aboveground part of medicinal herbs. From the 23 articles on the determination of medicinal materials from the whole plants, 54 elements such as Cu, Cd, Pb, As, Ni, Zn, and Mn were determined, of which 19 were Cu, accounting for 82.61%. Among the 23 articles on the determination of medicinal materials from the whole plants, 18 belong to traditional Chinese medicine, accounting for 78%. Three articles belong to Arabic medicine accounting for 13%. One article belongs to Tibetan medicine, accounting for 4%. One article belongs to the medical system of Miao medicine in China, accounting for 4%. The application of ICP-MS in medicinal materials from the whole plants is shown in Table 6.

**TABLE 6:** Application of ICP-MS in the determination of heavy metal elements in medicinal materials from the whole plants.

Latin name	Medical system	Therapeutic use	Main pharmacological effects	Origin of medicinal materials^a^	Test results
*Acalypha australis* L.	Traditional Chinese medicine	Infantile diarrhea, hematochezia	Anti-inflammatory, antioxidant, and anticancer	Sichuan province of China	The contents of 11 heavy metals of Pb, As, Hg, Cd, Cr, Cu, Ti, Ni, Co, Sb, and Zn in *A. australis* L. were determined. The linear relationship of 11 heavy metal elements is good (*R* ≥ 0.9992), and the detection limit of the method is 0.250–5.600 μg/kg, the recovery is 88.1%–107.8%, and RSD ≤ 2.39%. Repeatability test: RSD ≤ 3.43%, stability test: RSD ≤ 2.19%, precision test: RSD ≤ 2.03% (Sun et al., 2019)
*Aconitum tanguticum* (Maxim.) Stapf	Traditional Chinese medicine	Sores, food poisoning, snakebite	Analgesic, anti-inflammatory, and anti-tumor	—	Ten trace elements of Mn, Fe, Co, As, Ni, Se, Cu, Zn, V, and Cr in *A. tanguticum* (Maxim.) Stapf were determined. The recovery was 94.08%–103.50% (Limao et al., 2011)
*Artemisia rupestris* L.	Traditional Chinese medicine	Urticaria, dyspepsia, cold, hepatitis, allergic diseases	Antibacterial, anti-inflammatory, anti-allergic, and antioxidant	—	The contents of 15 trace elements of Li, B, V, Cr, Mn, Fe, Co, Ni, Cu, Zn, Mo, Cd, Tl, Pb, and Bi in *A. rupestris* L. were determined. The recoveries of these 15 elements were 80.06%–111.00%, and the relative standard deviation was less than 5.00% (Zhang and Xie, 2014)
*Chloranthus multistachys* C. Pei	Traditional Chinese medicine	Fracture, cold, skin itching	Anti-inflammatory, analgesic, and antibacterial	Kaili, Guizhou province, China	The contents of 14 Trace elements such as Cu, Pb, Cr, Ni, be, Mo, Ag, Sn, Sb, Tl, V, Co, As, and Cd in *C. multistachys* C. Pei were determined. The linear relationship of 14 elements meets the analysis requirements, and the correlation coefficient *r* > 0.9945. Relative standard deviation of repeatability: RSD < 5.0%. Relative standard deviation of precision: RSD < 4%. Recovery range: 84.0%–120.00% (Sun et al., 2021)
*Cymbopogon citratus* (DC.) Stapf	Arabic medicine	Gastrospasm	Anti-inflammatory and antihypertensive	Saudi Arabia	The contents of Al, Pb, Cd, and As in *C. citratus* (DC.) Stapf were determined. The recovery of all measured elements was 86.1%–90.6%. The content of Al element is the highest, and the concentration range of Al is 156–1609 mg/kg. The content of Cd is the lowest, and the concentration of Cd is in the range of 0.01–0.10 mg/kg. The washing process reduces toxic elements in all plants. The average % recoveries were Al (47.32%), As (59.1%), Cd (62.03%), and Pb (32.40%) (Brima, 2017)
*Elephantopus scaber* L.	Traditional Chinese medicine	Cold, conjunctivitis, pharyngitis, acute tonsillitis, epidemic encephalitis B, pertussis	Anti-inflammatory, analgesic, antibacterial, anti-tumor, antioxidant	Hainan, Guangxi, Guangdong, and Anhui provinces or autonomous regions of China	With Ge, In, and Bi as internal standards, the residues of Pb, Cd, As, Hg, and Cu in *E. scaber* L. were determined. For each determined element, the correlation coefficient *r* of the standard curve is 0.9991–0.9999, the average recovery is 91.2%–104.7%, and the RSD value is 3.9%–4.8% (Sun, 2019)
*Epimedium sagittatum* (Siebold & Zucc.) Maxim.	Traditional Chinese medicine	Impotence, arthralgia	Anti-aging and anti-tumor	—	The contents of Mn, Fe, Co, Ni, Cu, Zn, Ga, Ge, as, Se, Rb, Sr, Y, Zr, Nb, Mo, Cd, Sn, Sb, Cs, Ba, La, Ce, Nd, Pr, Th, Sm, Ta, Hg, Pb, and Bi in *E. sagittatum* (Siebold & Zucc.) Maxim. were determined. Mass concentration range is 1.3 × 10^−2^–93.5 mg·kg^−l^, in which the content of Fe is 93.5 mg·kg^−l^ and the content of Mn is 76.6 mg·kg^−l^, which are the two heavy metal elements with the highest content (Wu et al., 2015a)
*Equisetum arvense* L.	Arabic medicine	Obstetric pain	Anti-inflammatory, analgesic, and bacteriostatic	Saudi Arabia	The contents of Fe, Mn, Zn, Cu, and Se in *E. arvense* L. were determined. Fe level is the highest and Se level is the lowest among all plants. The TE series levels of all elements in all plants are as follows: Fe 193.4–1757.9 μg/g, Mn 23.6–143.7 μg/g, Zn 15.4–32.7 μg/g, Se 0.13–0.92 μg/g, and Cu 11.3–21.8 μg/g (Brima, 2018)
*Equisetum hyemale* L.	Arabic medicine	Labor pain	Analgesic, hypoglycemic, antioxidant, anti-tumor, antibacterial, and antiviral	Saudi Arabia	The contents of Al, Pb, Cd, and As in *E. hyemale* L. were determined. The recovery of all measured elements was 86.1%–90.6%. The content of Al element is the highest, and the concentration range of Al is 156–1609 mg/kg. The content of Cd is the lowest, and the concentration of Cd is in the range of 0.01–0.10 mg/kg. The washing process reduces toxic elements in all plants. The average % recoveries were Al (47.32%), As (59.1%), Cd (62.03%), and Pb (32.40%) (Brima, 2017)
*Gynostemma pentaphyllum* (Thunb.) Makino	Traditional Chinese medicine	Cough, chronic tracheitis, infectious hepatitis	Anti-inflammatory, antiviral, anti-tumor, anti-aging, and hypoglycemic	Teng County, Guangxi Zhuang autonomous region of China	The order of 19 inorganic elements in *G. pentaphyllum* (Thunb.) Makino is Ca > Mg > P > Al > Fe > Ti > Sr > Ba > Mn > B > Zn > Cu > Ni > Pb > Cr > V > Li > CD > Co (Chen et al., 2011a)
*Houttuynia cordata* Thunb.	Traditional Chinese medicine	Inflammation, viral cold, tumor	Bacteriostasis, anti-inflammatory, anti-tumor, and anti-virus	Guizhou, Hebei, Fujian, Guangdong, Jiangsu, Hubei, Sichuan, Zhejiang, and Guangxi provinces or autonomous regions of China	1) The contents of 27 inorganic elements mg, K, Ca, Fe, Li, Be, B, Ti, V, Mn, Co, Ni, Zn, Ga, Sr, Sn, Sb, Ba, Tl, Bi, Al, Cr, Cu, As, Cd, Hg, and Pb in *H. cordata* Thunb. were determined. The linear relationship of 27 elements is good, and the correlation coefficient *R* ≥ 0.9986. RSD of precision and repeatability experiment ≤ 6.0%. The recovery was 95.12%–109.48%, RSD ≤ 6.0%. The contents of Mg, K, Ca, and Fe in *H. cordata* Thunb. were higher (Chen et al., 2019b)
					2) The contents of Mn, Zn, Cu, Co, Ti, Sn, Sb, Ba, Cr, Ni, As, Pb, Hg, and Cd in *H. cordata* Thunb. were determined. The linear relationship of each element is good in the range of 0.15–695 g/L. The correlation coefficient of the regression equation of each element is *r* > 0.9997, the detection limit is 0.002–5.120 g/L, the recovery is 97.6%, 103.8%, and the relative standard deviation is ≤3.15%. The contents of Cu, As, Pb, Hg, and Cd in *H. cordata* Thunb. were lower than the national standard limit (Li et al., 2018b)
*Hypericum japonicum* Thunb.	Traditional Chinese medicine	Acute and chronic hepatitis	Antibacterial, antimalarial, antiviral, and anti-tumor	Hebei, Jiangsu, Anhui, Guangxi, Fujian, Hunan, and Jiangxi provinces or autonomous regions of China	The contents of 21 kinds of inorganic elements in Radix Rehmanniae were determined. The linear ranges of mass concentrations of Fe, Mg, Ca, Al, K, Na, Zn, Co, Ni, Ba, Mn, P, Se, Ti, Sr, Cu, As, Cd, Cr, Pb, and Hg were, respectively, 50–250 μg/ml (*r* = 0.9972), 25–100 μg/ml (*r* = 0.9989), 25–100 μg/ml (*r* = 0.9977), 2.5–15 μg/ml (*r* = 0.9996), 25–150 μg/ml (*r* = 0.9991), 2.5–15 μg/ml (*r* = 0.9999), 2.5–10 μg/ml (*r* = 0.9999), 2.5–10 μg/ml (*r* = 0.9999), 2.5–10 μg/ml (*r* = 0.9999), 2.5–10 μg/ml (*r* = 0.9999), 2.5–10 μg/ml (*r* = 0.9998), 2.5–10 μg/ml (*r* = 0.9996), 0.5–2 μg/ml (*r* = 0.9995), 2.5–10 μg/ml (*r* = 0.9999), 0.5–2 μg/ml (*r* = 0.9983), 2.5–10 μg/ml (*r* = 0.9997), 2.5–10 μg/ml (*r* = 0.9999), 2.5–10 μg/ml (*r* = 0.9999), 2.5–10 μg/ml (*r* = 0.9999), 0.05–0.2 μg/ml (*r* = 0.9992), and 0.05–0.2 μg/ml (*r* = 0.9997). RSD of the precision, stability, and repeatability test is less than 5.0%. The recovery was 93.9%–106.9% (RSD was 0.22%–2.94%, *n* = 6) (Ma et al., 2017)
*Mosla chinensis* Maxim.	Traditional Chinese medicine	Cold, headache, abdominal pain	Antiviral agents, microorganisms, sedation, antioxidation	Jiangxi province of China	1) The contents of mineral elements in the stems and leaves of *M. chinensis* and the contents of 25 elements of K, Ca, P, Mg, Al, Mn, Ba, Fe, Zn, Na, Sr, B, Cu, Ti, Pb, Cr, Ga, V, Ni, Cd, Sn, Co, sb, Li, and Be in *M. chinensis* Maxim. were determined. The contents of major elements such as K, Ca, P, and Mg and trace elements such as Mn, Fe, Zn, and Sr are higher, and the content in leaves is generally higher than that in stems (Yi et al., 2012)
					2) The contents of K, Na, P, Al, Mn, Fe, Ca, Zn, Cu, Ba, Sr, B, Ti, Cr, Ga, V, Ni, Sn, Y, Co, Li, Sb, Be, Tl, Pb, Cd, La, Ce, Pr, Nd, Sm, Eu, Gd, Tb, Dy, Ho, Er, Tm, Yb, and Lu in the stems and leaves of *M. chinensis* Maxim. from four different habitats in Jiangxi, China, were determined. K. Na, Ca, Mg, Fe, Zn, Mn, and other elements are abundant (Xu et al., 2014)
*Mussaenda pubescens* Dryand.	Traditional Chinese medicine	Cold, heatstroke, tonsillitis, cough, diarrhea, dysentery, poisonous snakebite	Anti-inflammatory, antibacterial, anticholinergic, and antifertility	Guangxi, Hunan, and Jiangxi provinces or autonomous regions of China	The contents of V, Cr, Mn, Fe, Co, Ni, Cu, Zn, As, Sr, Cd, Sn, Sb, Ba, Hg, and Pb in *M. pubescens* Dryand from different producing areas in Guangxi, Hunan, and Jiangxi province were determined. The linear coefficient *r* > 0.9992, the RSD of the repeatability test < 2.5%, the RSD of the precision test is 0.91%–3.7%, and the recovery is 85.6%–109.4% (Chu et al., 2020)
*Nepeta coerulescens* Maxim.	Tibetan medicine	Oculopathy	Antibacterial	Qinghai province of China	The contents of Cr, Mn, Fe, Ni, Cu, Zn, As, Cd, Ag, and Pb in *N. coerulescens* Maxim. were determined. The contents of Fe, Zn, and Cu in *N. coerulescens* Maxim. are higher: Fe 763.50 mg/kg, Zn 204.10 mg/kg, and Cu 97.71 mg/kg, respectively, whereas the contents of As, Pb, and Cd are lower, between 0.09 mg/kg and 2.53 mg/kg (Ye et al., 2019)
*Odontosoria chinensis* (L.) J.Sm.	Traditional Chinese medicine	Cold, fever, enteritis, food poisoning, burns, pesticide poisoning	Antibacterial, antioxidant, anti-inflammatory, anti-tumor, hypoglycemic	Guizhou, Hunan, Sichuan, Chongqing, Fujian, Yunnan, Guangxi, and Jiangxi provinces, municipalities, or autonomous regions of China	The contents of Cu, As, Cd, Hg, and Pb in 14 batches of *O. chinensis* (L.) J.Sm. from different producing areas such as Yunnan, Guizhou, and Sichuan were determined. Cu 2.63–12.89 mg/kg (RSD53.1%), As 0.29–1.56 mg/kg (RSD59.2%), Cd 0.01–2.55 mg/kg (RSD131.1%), Hg 0.01–0.24 mg/kg (RSD131.1%), Pb 1.09–22.56 mg/kg (RSD129.4%) (Ming et al., 2020)
*Piper wallichii* (Miq.) Hand.-Mazz.	Miao medicine of China	Dysmenorrhea, impotence, cough	Analgesia	—	The contents of Pb, Cd, As, Hg, and Cu in *P. wallichii* (Miq.) Hand.-Mazz. were determined. The correlation coefficient of the standard curve of five elements is *r* > 0.9994, the recovery is 94.6%–107.4%, and RSD < 5% (Chen and Zhang, 2020)
*Pogostemon cablin* (Blanco) Benth.	Traditional Chinese medicine	Dampness and turbidity, diarrhea, turbid nose, headache	Anti-inflammatory, analgesic, antibacterial, and antiviral microorganisms	Hainan and Guangdong provinces of China	The contents of trace elements in *P. cablin* (Blanco) Benth. from different producing areas such as Hainan province, Zhanjiang, and Guangdong province were determined. *P. cablin* (Blanco) Benth. contains B, Na, Mg, AI, K, Ca, Ti, V, Cr, Mn, Fe, Co, Ni, Zn, Se, Sr, Mo, Sn, Cs, Ba, Ti, Ga, and Ge (Zhang et al., 2011)
*Scleromitrion diffusum* (Willd.) R. J. Wang	Traditional Chinese medicine	Various types of inflammation	Anti-inflammatory, anti-tumor, antioxidant, and anti-chemical mutagenesis	Anhui, Yunnan, and Hainan provinces of China	The contents of 14 trace elements Mn, Cr, Sr, Zn, Rb, B, Ni, Cu, V, Sn, As, Mo, Se, and Co in *S. diffusum* (Willd.) R. J. Wang and its easily mixed product *O. corymbosa* L. were determined. The linear relationship of 14 elements is good in the range of 0–500 g/L, the correlation coefficient *r* ≥ 0.9998, the detection limit is 0.0019–0.1644 g/L, and the recovery is 97.5%–120.0%. *S. diffusum* (Willd.) R. J. Wang contains more Sn elements, and the waterline contains more of the other 13 elements. The contents of Mn, Cr, Sr, Zn, and other elements in the two kinds of medicinal materials are relatively high (Wang and Jin, 2018)
*Oldenlandia corymbosa* L.	Traditional Chinese medicine	Malaria, cancer, appendicitis	Anti tumor, anti-inflammatory, antimalarial, antioxidant and antibacterial	Anhui, Yunnan and Hainan Provinces of China	
*Vincetoxicum mukdenense* Kitag.	Zhuang medicine of China	Snakebite, infantile chancre, dysentery, toothache	Analgesic, bacteriostatic, and anti-inflammatory	Guangxi Zhuang autonomous region of China	The contents of Pb, Cd, As, Hg, and Cu in *V. mukdenense* Kitag. were determined. The content range of each element is Pb 0.2301–0.6836 mg/kg, Cd 0.0717–0.1957 mg/kg, As 0.0250–0.0750 mg/kg, Hg 4.2794–7.7946 μg/kg, and Cu 0.9943–1.5444 mg/kg (Chen et al., 2018a)

a Note: the origin of medicinal materials recorded in the table is collected from the articles.

If the origin of medicinal materials is not recorded in the articles, it is indicated by “—.”

### Analysis and Application of ICP-MS in Other Medicinal Materials Derived From Plants and Soil

Other medicinal materials derived from plants included barks, resins, fruiting bodies, and medicinal materials parasitic on various parts of plants. From the 17 articles on other medicinal materials derived from plants and soil, 28 elements such as Cu, Cd, Pb, As, Hg, Zn, and Mn were determined, of which 15 were the most, accounting for 88.24%. Among the 17 articles on the determination of other medicinal materials derived from plants and soil, 16 belong to traditional Chinese medicine, accounting for 94%. One article belongs to Mongolian medicine in China, accounting for 6%. The application of ICP-MS in other medicinal materials derived from plants and soil is shown in Table 7.

**TABLE 7:** Application of ICP-MS in the determination of heavy metal elements in other medicinal materials derived from plants and soil.

Latin name	Medical system	Therapeutic use	Main pharmacological effects	Origin of medicinal materials^a^	Test results
*Angelica sinensis* (Oliv.) Diels	Traditional Chinese medicine	Irregular menstruation, amenorrhea, dysmenorrhea, constipation	Analgesic, anti-inflammatory, antibacterial, antioxidant, anti-senile dementia	Gansu and Yunnan Provinces of China	The detection limits of elements in *Angelica sinensis* (Oliv.) Diels soil in different areas of Gansu and Yunnan province were Pb 0.0213 μg/L, Cd 0.0196 μg/L, As 0.0168 μg/L, Hg 0.0215 μg/L, Cu 0.0241 μg/L, Cr 0.0192 μg/L, Sb 0.0189 μg/L, Ni 0.0176 μg/L, Zn 10 μg/L, Fe 18 μg/L, Mn 11 μg/L, Mg 3 μg/L, Ca 11 μg/L, Na 12 μg/L, and K 15 μg/L, respectively. The correlation coefficients are between 0.9989 and 1.0000 (Gu et al., 2014)
*Chrysanthemum morifolium* (Ramat.) Hemsl.	Traditional Chinese medicine	Tumor, inflammation, cardiovascular disease	Anti-inflammatory, antiviral, antibacterial, mutagenic, and anti-tumor	Bozhou, Anhui province of China	The contents of Mg, Al, Fe, Ba, Mn, Cr, Se, and Mo in *C. morifolium* (Ramat.) Hemsl. soil were determined. The average recoveries of the eight elements were 94.77%–124.11%, and the RSD values were 2.34%–7.96% (*n* = 6) (Yu et al., 2014)
*Cordyceps cicadae* Miquel.	Traditional Chinese medicine	Tumor, hyperglycemia	Anticonvulsant, antioxidant, anti-tumor	—	The contents of K, Na, Ca, Pb, Cd, Hg, As, and Cu in *C. cicadae* Miquel. were determined. The correlation coefficient of the standard curve of each element is 0.9990–0.9999, the recovery is 95.25%–103.73%, and the RSD value is less than 4% (Gu, 2020)
*Cordyceps militaris* Link.	Traditional Chinese medicine	Cough, acute and chronic bronchitis, asthma	Anti-inflammatory, bacteriostatic, and anti-tumor	—	The contents of Ca, Fe, Zn, and Se in commercial *C. militaris* Link. Samples were detected. The Zn content of *C. militaris* Link. is generally high, and other elements are at the normal level (Xiao, 2019)
*Cordyceps sinensis* Berk.	Traditional Chinese medicine	Impotence, lumbago, cough	Anti-oxidation, anti-tumor, and anti-aging	Qinghai and Sichuan provinces of China	1) The contents of various elements in *C. sinensis* Berk. soil in Qinghai and Sichuan provinces of China were determined. As is 7.60–20.60 mg/kg, Pb 18.16–28.44 mg/kg, Cr 20.15–66.31 mg/kg, Cu 22.35–35.23 mg/kg, and Cd 0.06–1.48 mg/kg (Chen et al., 2018b)
					2) The residues of Pb, Cd, As, Hg, and Cu in fresh *C. sinensis* Berk. were determined. The linear relationship of the five heavy metals in their respective mass concentration range was good, *r* > 0.996, and the average recovery was 99.55%–121.73% (RSD < 4.3%) (Qian et al., 2019a)
*Ganoderma lucidum* (Curtis) P. Karst.	Traditional Chinese medicine	Insomnia, cough	Anti-tumor, anti-aging, prevention and treatment of cardiovascular diseases, and protection of liver injury	Anhui, Zhejiang, Shandong, Fujian, Guangdong, and Jilin provinces of China	The contents of heavy metals in *G. lucidum* (Curtis) P. Karst. Spore powder from different producing areas such as Anhui, Zhejiang, and Shandong were determined. Pb is 0.19 mg·kg^−1^, As is 0.16 mg·kg^−1^, Hg is 0.02 mg·kg^−1^, Cu is 14.73 mg·kg^−1^, and Cd is 0.34 mg·kg^−1^. After wall breaking, Pb was 0.21 mg·kg^−1^, As was 0.12 mg·kg^−1^, Hg was 0.02 mg·kg^−1^, Cu was 15.81 mg·kg^−1^ and Cd was 0.37 mg·kg^−1^ (Hu et al., 2016)
*Garcinia hanburyi* Hook.f.	Mongolian medicine of China	Tumor, anabrosis, hemorrhage	Anti-inflammatory, antibacterial, antioxidant, and anti-tumor	—	The contents of Pb, Cd, As, Hg, and Cu in *G. hanburyi* Hook.f. were determined. The linear relationship between the five elements is good (*r* ≥ 0.9995), and the mass concentrations of Pb, Cd, and As are 0–50 μg·L^−1^. The linear relationship is good, and the detection limits are Pb 0.020 μg·L^−1^ Cd 0.012 μg·L^−1^, As 0.035 μg·L^−1^. The mass concentration of Cu is 0–500 μg·L^−1^. The linear relationship is good, and the detection limit is 0.05 μg·L^−1^. The mass concentration of Hg is 0–5 μg·L^−1^. The linear relationship is good, and the detection limit is 0.007 μg·L^−1^ (Yan et al., 2020)
*Conioselinum anthriscoides* “Chuanxiong”	Traditional Chinese medicine	Heartache, thoracalgia, irregular menstruation, amenorrhea, dysmenorrhea, headache	Anti-tumor, anticoagulant, anti-cerebral ischemia, and anti-depression	Sichuan province of China	The contents of Cd, As, Cu, and Pb in *C. anthriscoides* “Chuanxiong” soil were determined. The linearity of each element in the corresponding range is good (*r* > 0.999), the precision is good (RSD < 4%, *n* = 6), and the recovery is 92.7%–107.9% (Chen et al., 2014b)
*Magnolia officinalis* Rehder & E. H. Wilson	Traditional Chinese medicine	Tumor, inflammation, dental caries	Anti-epilepsy, anti-depression, anti-dementia, anti-cerebral ischemia, anti-tumor, hypoglycemic	Sichuan, Hubei, and Shanxi provinces of China	The contents of Mn, Fe, Ni, Cu, Zn, Rb, Sr, Cd, Ba, and Pb in *M. officinalis* Rehder & E. H. Wilson from different producing areas in Sichuan, Hubei, and Shanxi provinces of China were determined. The standard curve of each element has a good linear relationship (*r* = 0.9998–1.0000). The recovery of standard addition is 90.57%–114.56%. There were significant differences in the content of metal elements in *M. officinalis* Rehder & E. H. Wilson samples from different producing areas, and the content of heavy metals in some medicinal materials exceeded the standard (Fang et al., 2018)
*Phellodendron amurense* Rupr.	Traditional Chinese medicine	Spermatorrhea, eczema	Anti-inflammatory, antibacterial, anticancer, hypoglycemic, and hypotensive	—	The contents of Co, Ni, Cu, Zn, As, Cd, Hg, and Pb in *P. amurense* Rupr. were determined. The relative standard deviation of each element was 3.2%–17.8%, and the recovery was 70%–120% (Kou et al., 2007)
*Polyporus umbellatus* Pers.	Traditional Chinese medicine	Edema, diarrhea	Anti-inflammatory, anti-tumor, antioxidant, antibacterial, and anti-mutation	Anhui, Hunan, Yunnan, Guangxi, and Hunan provinces or autonomous regions of China	The contents of Be, Na, Mg, Al, K, Ca, V, Cr, Mn, Fe, Co, Ni, Cu, Zn, As, Se, Mo, Ag, Cd, Sb, Ba, Hg, Tl, Pb, and U in *P. umbellatus* Pers. from different places in Liaoning, Shanxi, Shaanxi, Anhui, and Inner Mongolia provinces of China were determined. Cu ≤ 20.0 μg·g^−1^, As ≤ 2.0 μg·g^−1^, Cd ≤ 0.3 μg·g^−1^, Hg ≤ 0.2 μg·g^−1^, Pb ≤ 5.0 μg·g^−1^ (Lao et al., 2016)
*Poria cocos* (Schw.) Wolf.	Traditional Chinese medicine	Loose stools, diarrhea, uneasiness, Insomnia	Anti-inflammatory, anti-tumor, and hypolipidemic	Liaoning, Shanxi, Shaanxi, Anhui, and Inner Mongolia provinces or autonomous regions of China	The contents of Be, Na, Mg, Al, K, Ca, V, Cr, Mn, Fe, Co, Ni, Cu, Zn, As, Se, Mo, Ag, Cd, Sb, Ba, Hg, Tl, Pb, and U in *P. cocos* (Schw.) Wolf. from different places in Liaoning, Shanxi, Shaanxi, Anhui, and Inner Mongolia provinces of China were determined. Cu ≤ 20.0 μg·g^−1^, As ≤ 2.0 μg·g^−1^, Cd ≤ 0.3 μg·g^−1^, Hg ≤ 0.2 μg·g^−1^, Pb ≤ 5.0 μg·g^−1^ (Lao et al., 2016)
*Rheum tanguticum* (Maxim. ex Regel) Balf.	Traditional Chinese medicine	Constipation, pharyngeal swelling, amenorrhea, water fire scald, upper gastrointestinal bleeding	Anti-inflammatory, anti-tumor, and lipid-lowering	—	The contents of Pb, Cd, Hg, Cu, and As in *R. tanguticum* (Maxim. ex Regel) Balf. soil in the Songpan area were determined. The standard curve of five elements had a good linear relationship, and the correlation coefficients were between 0.9996 and 0.9999 (Wang et al., 2014a)
*Salvia miltiorrhiza* Bunge	Traditional Chinese medicine	Irregular menstruation, insomnia, angina pectoris	Anti-inflammation, anti-tumor, anti-oxidation, anti-hypertension, anti-hyperlipidemia, and anti-liver injury	Shanxi, Henan, Hubei, Hunan, Shanxi, Jiangxi, Shandong, Anhui, Zhejiang, and Liaoning provinces of China	The contents of Mg, Al, K, Ca, Ba, Mn, Zn, Cu, Cd, and Pb in wild *S. miltiorrhiza* Bunge soils from 27 different producing areas in Shaanxi, Hunan, and Henan were determined. Al > K > Ca > Mg > Ba > Mn > Zn > Cu > Pb > Cd (Yu et al., 2017b)
*Viscum articulatum* Burm.f.	Traditional Chinese medicine	Hypertension, arrhythmia, tumor, hepatitis	Anti-tumor, antihypertensive, antiarrhythmic, anti-aging, and antiviral	—	The contents of 18 trace elements of Na, Mg, Al, K, Ca, Cr, Mn, Fe, Ni, Cu, Zn, Ge, As, Se, Mo, Cd, Hg, and Pb in *V. articulatum* Burm.f. on tea tree were determined. The detection limit is 0.03363–26.59 μg/L, the relative standard deviation (RSD, *n* = 6) is 1.54%–7.57%, and the recovery is 90.17%–109.7%. The contents of K, Ca, Mg, and Al in *V. articulatum* Burm.f. are high (Wu et al., 2011)

a Note: the origin of medicinal materials recorded in the table is collected from the articles.

If the origin of medicinal materials is not recorded in the articles, it is indicated by “—.”

## Analysis and Application of ICP-MS in Medicinal Materials Derived From Animals

Medicinal materials derived from animals refer to a kind of traditional Chinese medicine used for medicine, such as the whole or part of animals, the physiological or pathological products of animals, and the processed products of animals. It has a very long medicinal history. Medicinal materials derived from animals are rich in metal trace elements. Many researchers evaluate the quality of medicinal materials derived from animals by controlling metal trace elements (Wang et al., 2015b). Among the various metal elements contained in medicinal materials derived from animals, some elements are necessary for the human body and the material basis for human growth, body metabolism, and physiological function regulation. Some other elements are harmful elements, such as Pb, Cd, As, Hg, Cu, and other elements. If these elements exceed the standard, they will directly affect the health of patients and damage the function of some parts of the body. In addition, some elements are potentially radiotoxic elements, and long-term exposure will also cause damage to the human body (Chi et al., 2016).

**FIGURE 3:**
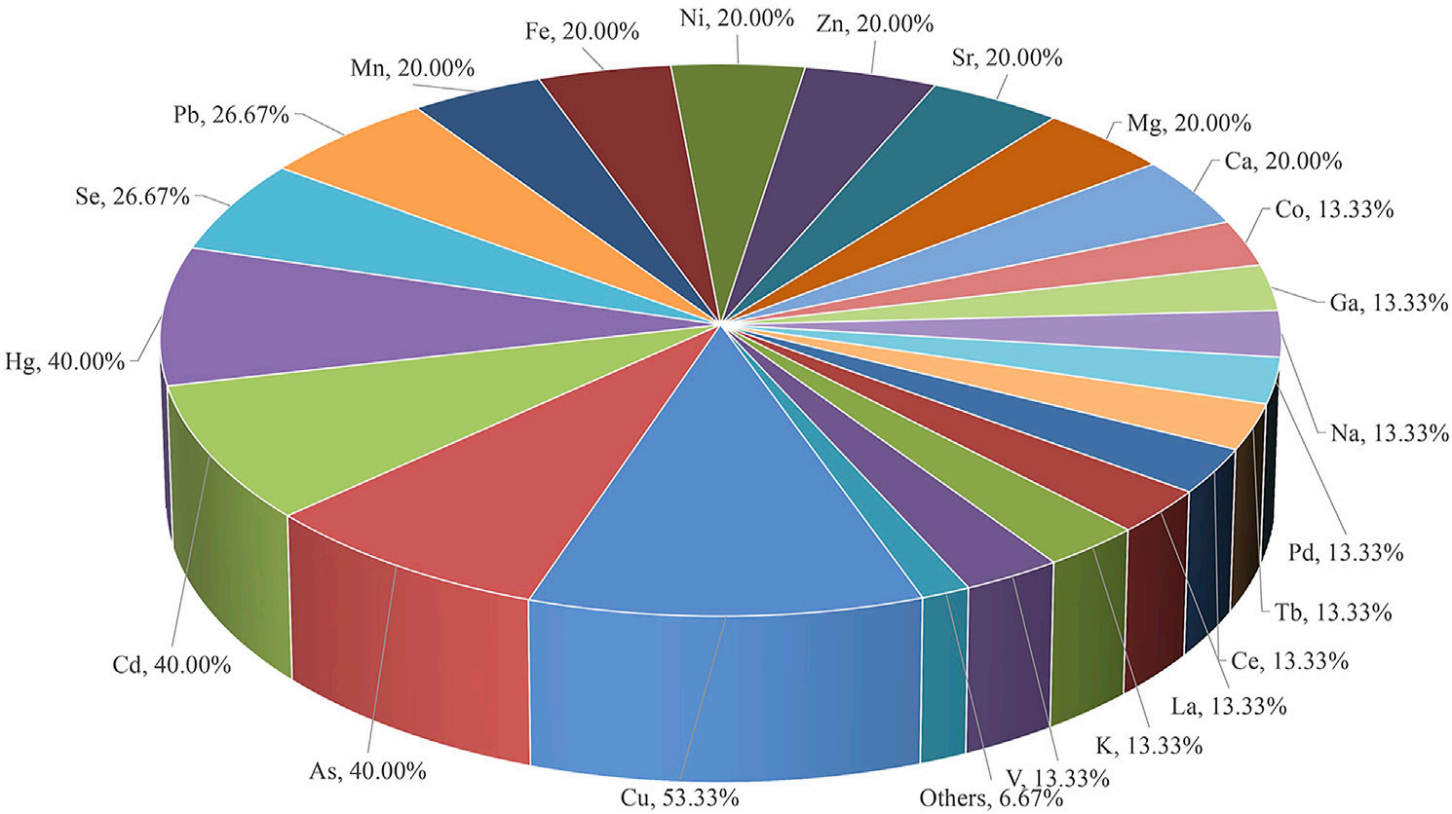
Pie chart of elements measured by ICP-MS and their proportions in medicinal materials derived from animals.

At present, there are few reports on the safety of heavy metal residues in medicinal materials derived from animals. The determination methods of heavy metals and harmful elements mainly include atomic absorption spectrometry, atomic fluorescence spectrometry, and atomic emission spectrometry. Most of them can only be used for the determination of single elements. The ICP-MS method, which can simultaneously analyze and determine multi-element, has the advantages of high sensitivity, low detection limit, and wide linear range. Zuo et al. (2017) used ICP-MS to determine the residues of heavy metals and harmful elements in 18 common animal medicinal materials such as gecko, cicada molt, centipede, and leech. Among them, Pb, Cd, As, and Ag exceed the standard. The quality of medicinal materials derived from animals should be focused on, and the limit standard of heavy metals and harmful elements in medicinal materials derived from animals from the perspective of risk assessment should be improved. The rest are shown in Table 8. It provides a basis for further improving the quality of medicinal materials derived from animals and ensuring their drug safety and provides a basic reference for the study of the speciation and valence of heavy metals in medicinal materials derived from animals. From the 15 articles on medicinal materials derived from animals determined, 49 elements such as Cu, As, Cd, Hg, Se, Pb, and Mn were determined, of which eight were Cu, accounting for 53.33%. The elements determined by ICP-MS and their proportion in animal-derived medicinal materials are shown in Figure 3. From the 15 articles on the determination of medicinal materials derived from animals, all animal-derived medicinal materials belong to traditional Chinese medicine.

**TABLE 8:** Application of ICP-MS in the determination of heavy metal elements in medicinal materials derived from animals.

Latin name	Medical system	Therapeutic use	Main pharmacological effects	Origin of medicinal materials^a^	Test results
*Bombyx batryticatus* Bals.	Traditional Chinese medicine	Tetanus, apoplexia, headache, pharyngalgia	Anticoagulant, antithrombotic, bacteriostatic, anticonvulsant, antibacterial	Heilongjiang, Anhui, Jiangsu, Henan, Liaoning, Beijing, Jilin, Shanxi, Hebei, and Sichuan provinces or municipalities of China	The contents of Pb, Hg, As, Cd, and Cu in 10 batches of commercial medicinal materials of *B. batryticatus* Bals. were determined, which met the limit requirements of the Chinese Pharmacopoeia for harmful elements (Guo et al., 2012)
*Bungarus parvus* Blyth.	Traditional Chinese medicine	Tetanus, apoplexia	Anti-inflammatory, analgesic, and anti-tumor	—	The contents of five harmful elements of Pb, Hg, As, Cd, and Cu in eight batches of commercial medicinal materials of *B. parvus* Blyth. were determined. The content of Cd mostly exceeds the standard, and the content of Pb and Cu in some samples exceeds the standard (Wu et al., 2015a)
*Carapax trionycis* Wiegmann	Traditional Chinese medicine	Dizziness, amenorrhea	Anti-liver fibrosis and anti-cancer	Changsha, Hunan province of China	The contents of 10 inorganic elements of Na, Mg, K, Ca, Mn, Fe, Ni, Cu, Zn, and Se in 10 batches of *C. trionycis* Wiegmann were determined. The characteristic elements of *C. trionycis* Wiegmann are Fe, Mn, Zn, Ca, and Cu (Ma et al., 2011)
*Cervus elaphus* Linnaeus	Traditional Chinese medicine	Dizziness, lumbago	Anti-mammary hyperplasia, gastric mucosal protection, anti-osteoporosis, anti-senile dementia	—	The contents of eight trace elements of Ca, Mg, Fe, Zn, Mn, Cu, Sr, and Se in *C. elaphus* Linnaeus gum and *C. reevesii* Gray gum were determined. The recoveries of eight elements (*n* = 6) were 88.89%–105.31%, the detection limit was low, and the RSD was less than 6.0% (Wang et al., 2018b)
*Chinemys reevesii* Gray	Traditional Chinese medicine	Bleeding, arrhythmia	Promote cell proliferation and inhibit apoptosis	—	
*Eupolyphaga sinensis* Walker	Traditional Chinese medicine	Liver disease and tumor	Antithrombotic, anticoagulant, anti-tumor, and anti-ischemia	—	The contents of harmful elements in *E. sinensis* Walker were Hg 0.11 mg/kg, Cu 272.20 mg/kg, Cd 0.04 mg/kg, Pb 2.08 mg/kg, As 1.19 mg/kg, respectively (Cao et al., 2019)
*Gekko gecko* Linnaeus	Traditional Chinese medicine	Hemoptysis, impotence, spermatorrhea, and asthma	Anti-tumor	—	The contents of B, Na, Al, Mg, P, K, Ca, Sc, V, Cr, Mn, Fe, Co, Ni, Cu, Tb, Zn, Ga, Ge, As, Se, Sr, Mo, Cd, In, Ba, La, Ce, Hg, Pb, Bi, and Th in *G. gecko* Linnaeus were determined. The contents of Ca, P, K, Na, and Mg are the highest in major elements, and the contents of Fe, Zn, Se, Cr, and Cu are the highest in essential trace elements (Yao et al., 2010)
*Haliotis discus* Reeve.	Traditional Chinese medicine	Vertigo	Antibacterial, antioxidant, and antihypertensive	Guangxi, Hainan, Guangdong, Zhejiang, and Fujian Provinces or autonomous regions of China	The contents of 15 rare earth elements of Y, Tb, La, Dy, Ce, Ho, Pr, Er, Nd, Tm, Sm, Yb, Eu, and Lu in *H. discus* Reeve., *H. cumingii* Lea., *O. gigas* Tnunb. were determined. The linearity of each element in the corresponding range is good (*r* > 0.999), the precision is good (RSD < 4%, *n* = 6), and the recovery of each element is 97.0%–105.0% (Chen et al., 2014a)
*Hyriopsis cumingii* Lea.	Traditional Chinese medicine	Anabrosis	Antibacterial and antioxidant	Guangxi, Guangdong, Jiangxi, and Zhejiang Provinces or autonomous regions of China	
*Ostrea gigas* Tnunb.	Traditional Chinese medicine	Insomnia, headache, dizziness, stomachache	Antioxidant, anti-tumor, hypoglycemic	Guangxi, Hebei, Guangdong, Fujian, Liaoning, and Zhejiang provinces or autonomous regions of China	
*Spongilla fragilis* Leidy.	Traditional Chinese medicine	Impotence, spermatorrhea	Bacteriostasis	—	The contents of Hg, Cu, Cd, Pd, and As in *S. fragilis* Leidy. were determined. For each determined element, the correlation coefficient of the standard curve is *r* = 0.998–0.9999, the recovery is 80.3%–105.2%, and the RSD value is 0.5%–12.1% (Li, 2018a)
*Aspongopus chinensis* Dallas.	Traditional Chinese medicine	Stomachache, hepatodynia, impotence	Antibacterial, anti-tumor, and antioxidant	—	The contents of Be, V, Co, Ni, Ga, Se, Rb, Sr, Ag, Cs, U, Cu, Co, Ni, Ga, Se, Rb, Sr, Ag, Cs, and U in *A. chinensis* Dallas., *E. sinensis* Walker, *P. asiatica* Perrier., *P. olivaceus* DeGeer. were determined. The residues of four kinds of medicinal materials derived from animals were higher (Li et al., 2020b)
*Eupolyphaga sinensis* Walker	Traditional Chinese medicine	Traumatic injury, fracture, amenorrhea	Antithrombotic, anticoagulant, anti-tumor, and anti-ischemia	—	
*Pheretima asiatica* Perrier.	Traditional Chinese medicine	Asthma, cough, edema, hypertension	Anti-inflammation, antithrombotic, heart protection, anti-tumor, and improvement of the respiratory system	—	
*Polistes olivaceus* DeGeer.	Traditional Chinese medicine	Toothache, rheumatism	Antibacterial, anti-inflammatory, anti-cancer, anti-ulcer and anti-virus	—	

a Note: the origin of medicinal materials recorded in the table is collected from the articles.

If the origin of medicinal materials is not recorded in the articles, it is indicated by “—.”

## Analysis and Application of ICP-MS in Medicinal Materials Derived From Minerals

Medicinal materials derived from minerals are rich in resources, unique curative effects, and numerous varieties. However, due to a large number of mineral sources, different mineral sources, and great differences in quality, the research on the quality standard of mineral traditional Chinese medicine is of great significance in clinical application. Mineral traditional Chinese medicine can be divided into raw medicinal materials derived from minerals (such as cinnabar, calamine, and natural copper), processed products of mineral raw materials (such as mirabilite and light powder) or fossils of animal bones (such as petrel, keel, and pumice). At present, the quality evaluation of mineral traditional Chinese medicine includes experience identification, purity inspection, and content determination. Empirical identification of the characteristics of mineral medicinal materials can reflect the quality of medicinal materials to a certain extent. For example, ochre is brown-red in color, with an obvious cross-section level with “nailhead” and no miscellaneous stones are preferred (Hu, 2016). However, due to the high similarity of the external morphological characteristics of medicinal materials derived from minerals and more confused products, the practitioners of traditional Chinese medicine often lack the professional knowledge of mineralogy, so character identification needs long-term experience accumulation and inheritance. The application of modern instrumental analysis methods in the identification and quality evaluation of medicinal materials derived from minerals is gradually expanding. Among them, ICP-MS is becoming the key technology for mineral drug evaluation (Li et al., 2018a).

**FIGURE 4:**
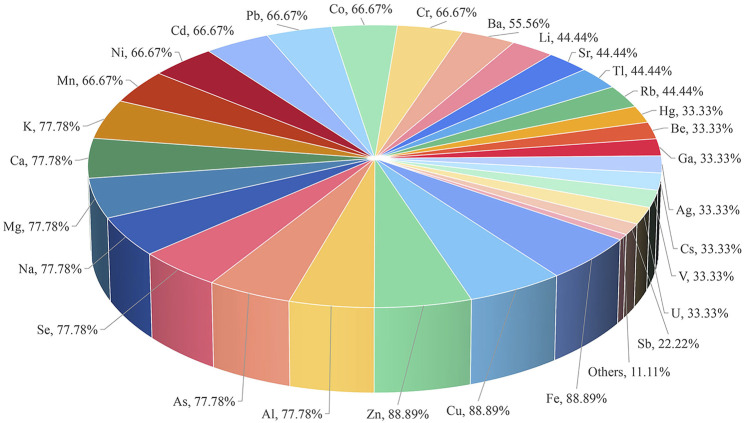
Pie chart of elements measured by ICP-MS and their proportions in medicinal materials derived from minerals.

In recent years, the relationship between the types and contents of inorganic elements in traditional Chinese medicine and their efficacy has attracted extensive attention. The content determination of these elements, including main elements and trace elements, can provide not only important data for the quality evaluation of traditional Chinese medicine but also a reference for the clarification of its action mechanism. As an important member of the family of traditional Chinese medicine, medicinal materials derived from minerals deserve special attention for their safety and effectiveness. Hu (2016) used ICP-MS combined with microwave digestion. Comparing the contents of trace elements of Pb, Cu, Mn, Ni, Cr, Zn, As, Ti, and Cd in eight mineral traditional Chinese medicine pieces of the calcined keel, calcined oyster, gypsum, talc, Mircanite, ochre, amber, and valerian seed with their formula granules, the results show that there are some differences in the contents of trace elements between mineral traditional Chinese medicine and its formula granules. Zhou et al. (2015) used the ICP-MS method to determine the content of trace elements in six common mineral Chinese herbal materials, namely, raw keel, calcined keel, raw oyster, calcined oyster, raw gypsum, and talc, established the detection method of corresponding elements, and made comparative analysis on the content of trace elements in the raw calcined keel and raw calcined oyster. It provides a method for the determination of trace elements in different mineral Chinese medicinal materials from different producing areas. The rest is shown in Table 9. From the nine articles of medicinal materials derived from minerals determined, 70 elements such as Fe, Cu, Zn, Al, As, Se, and Na were determined, of which eight were Fe, Cu, and Zn, accounting for 88.89%. The elements determined by ICP-MS and their proportion in mineral raw materials are shown in Figure 4. In the nine articles on the determination of medicinal materials derived from minerals, the measured medicinal materials derived from minerals belong to traditional Chinese medicine.

**TABLE 9:** Application of ICP-MS in the determination of heavy metal elements in medicinal materials derived from minerals.

Name	Medical system	Chemical formula	Therapeutic use	Main pharmacological effects	Origin of medicinal materials^a^	Test results
Alumen	Traditional Chinese medicine	KAl (SO_4_)_2_·12H_2_O	Hematochezia, hemorrhoids	Antibacterial and anticancer	—	The contents of K, Al, Fe, Zn, Cu, Pb, Cd, As, and Hg in Alumen and its fake ammonium Alumen were determined. Alumen mainly contains Al and K elements, and ammonium Alumen mainly contains Al and Fe elements. The content of K element is far lower than that of K element in medicinal Alumen (Geng et al., 2020)
Cinnabar	Traditional Chinese medicine	HgS	Insomnia	Hypnosis and anti-anxiety	Guangxi, Guangdong, Henan, Shandong, Guizhou, and Yunnan provinces or autonomous regions of China	The contents of total Hg and soluble Hg in Cinnabar from different producing areas such as Sichuan, Hunan, and Jiangsu were determined. The linear correlation coefficient *r* is 1.000. The recoveries were 95%–105%. Precision RSD ≤ 1%. Repeatability RSD ≤ 0.4%. Stability: RSD of mercuric sulfide ≤ 0.1%, RSD of soluble mercury salt (calculated by Hg) ≤ 1%. Method detection limit (LOQ): mercury sulfide was 6 mg·g^−1^ and soluble mercury salt (calculated by Hg) was 0.06 μg·g^−1^ (Lan, 2014)
Gypsum	Traditional Chinese medicine	CaSO_4_·2H_2_O	Headache, toothache	Antiviral	—	The contents of Ca, Mg, Zn, Na, Al, and Se in raw Gypsum and calcined Gypsum were determined. After processing, the contents of Ca, Mg, Zn, and Na increased, whereas the contents of Al and Se decreased (Bao et al., 2012)
Kaolinite	Traditional Chinese medicine	Al2(SiO_3_)_3_·4H_2_O	Spermatorrhea, bleeding	Antidiarrheal	Gansu, Anhui, Henan, Shanxi, Jiangsu, Shandong, Shanxi, and Hubei provinces of China	The contents of 27 heavy metals and trace elements of Li, Be, Na, Mg, Al, K, Ca, Cr, Mn, Fe, Co, Ni, Cu, Zn, Ga, As, Se, Rb, Sr, Ag, Cd, Cs, Ba, Pb, Tl, U, and V in Kaolinite from different producing areas in Gansu, Anhui, Henan, Shaanxi, Jiangsu, Shandong, Shanxi, and Hubei provinces of China were determined. Al is the element with the highest content. Li, Na, Mg, K, Ca, V, Mn, Fe, Co, Ni, Zn, Ga, Se, Rb, Sr, Ba, and U are the main components of trace elements and can be used as characteristic elements (Zhu et al., 2019)
Mirabilite	Traditional Chinese medicine	Na_2_SO_4_·10H_2_O	Constipation, bellyache, hemorrhoids	Anti-inflammatory	Shanxi, Sichuan, Hunan, Shanxi, Shandong, Qinghai, and Anhui provinces of China	24 elements of Li, Be, Na, Mg, Al, K, Ca, Cr, Mn, Fe, Co, Ni, Cu, Zn, Ga, As, Se, Rb, Sr, Cd, Cs, Ba, Tl, and Pb in Mirabilite were determined. The contents of Na, Mg, K, Ca, Fe, and other elements are high. In addition, heavy metals and harmful elements Pb, Cd, Cu, and As were also detected (Li et al., 2013)
Natrii Sulfas	Traditional Chinese medicine	CuSO_4_·5H_2_O	Constipation, bellyache, hemorrhoids	Sterilization	—	Determination of the element composition of Natrii Sulfas decoction, Na, Mg, Al, K, Ca, V, Cr, Mn, Fe, Fe, Co, Ni, Cu, Zn, As, Se, Ag, Cd, Sb, Ba, Tl, Pb, and U. The main elements are Cu, Zn, Fe, Cd, Mn, Al, Na, and Ca (Wang et al., 2017a)
preparations of Glauber’s salt and liquorice	Traditional Chinese medicine	Na_2_SO_4_	Constipation	Diarrhea, anti-inflammation, and bacteriostasis	Shanxi, Sichuan, Hunan, Gansu, Shanxi, Hebei, Qinghai, and Anhui provinces of China	Determination of 20 elements of Li, Na, Mg, Al, K, Ca, Cr, Mn, Fe, Co, Ni, Cu, Zn, As, Se, Rb, Cd, Sr, Ba, and Pb in preparations of Glauber’s salt and liquorice. The contents of Na, Mg, K, Ca, Fe, Zn, and Sr are high, and heavy metals and harmful elements Pb, Cd, Cu, and As are also detected (Li et al., 2014)
Realgar	Traditional Chinese medicine	As_2_S_2_	Snakebite	Antiviral and anti-tumor	—	The contents of Na, Mg, K, Ca, Fe, Cu, Zn, Cr, Mn, Co, Ni, Se, and As in mice liver were determined. As in Realgar can cause the changes in Mg, Ca, Fe, Cu, Zn, Cr, Mn, Co, Ni, and Se levels in the liver, but it has no obvious effect on Na and K levels (Wang et al., 2018a)
Sulfur	Traditional Chinese medicine	S	Constipation, scabies, impotence	Sterilization	Shaanxi, Hebei, Shanxi, Shandong, Henan, Yunnan, Jiangsu, Fujian, Sichuan, Gansu, and Anhui provinces of China	The contents of Ca, Fe, Zn, Mg, Na, Al, K, Li, P, Ti, Cr, Mn, Ni, Cu, As, Ba, Pb, Be, B, Sc, V, Co, Ga, Ge, Br, Se, Kr, Rb, Sr, Y, Zr, Nb, Mo, Ru, Rh, Pd, Ag, Cd, In, Sn, Sb, Te, I, Xe, Cs, La, Ce, Pr, Nd, Sm, Eu, Gd, Tb, Dy, Ho, Er, Tm, Yb, Lu, Hf, Ta, W, Os, Pt, Au, Hg, Tl, Bi, Th, and U 70 heavy metals in sulfur were determined. Ca, Na, Al, K, Ti, As, and other elements are characteristic trace elements (Zhao et al., 2020b)

a Note: the origin of medicinal materials recorded in the table is collected from the articles.

If the origin of medicinal materials is not recorded in the articles, it is indicated by “—.”

## Application of ICP-MS in Chinese Patent Medicine

Chinese patent medicine is based on the theory of Chinese medicine, which is made from Chinese herbal medicines according to the prescribed prescription, production technology, and quality standard. It is the cream of effective prescriptions created and summarized by China’s medical practitioners in past centuries. However, the method of detection and control of inorganic elements in China patent medicine is in the Chinese drug standard and even the Pharmacopoeia. The degree of attention is not high. Currently, many researchers have begun to focus on the problems of trace elements, heavy metals, and harmful elements in Chinese patent medicine (Fu, 2017).

Inorganic elements have a strong ability to form complexes and easily form coordination bonds with ligands containing N, O, and S in organisms to coordinate the material balance in the body (Zhao et al., 2019). The content and type of inorganic elements in traditional Chinese medicine can affect its nature, taste, and efficacy. For example, Zn, Ca, and Fe are the characteristic inorganic elements of hemostatic traditional Chinese medicine. K and Mg can affect the properties of drugs for promoting blood circulation and removing blood stasis. The difference between Fe and Mn content is the basis of the “cold and warm” property (Wu et al., 2014). It shows that the type and content of inorganic elements have a certain synergistic effect on the exertion of drug efficacy. In Chinese patent medicine, clarifying the types and characteristics of inorganic elements can provide a reference for the efficacy study of the preparations from the perspective of inorganic elements. As we all know, the quality and safety of raw materials directly affect the efficacy and drug safety of finished products. With the advancement of China’s industrialization, the pollution of soil and water by heavy metals and harmful elements is becoming more and more serious, resulting in the possible pollution of native traditional Chinese medicine in various links such as growth, collection and processing, warehousing and transportation, and even the production process of preparations (Yan et al., 2017). In recent years, due to the frequent exposure to heavy metal pollution in traditional Chinese medicine, South Korea, the United States, and other countries have raised the safety testing standards of traditional Chinese medicine and restricted the import of traditional Chinese medicine. At present, in China’s drug standards, only a few processed products of traditional Chinese Medicine (Zhong yao yin pian), such as *Glycyrrhiza uralensis* Fisch. ex DC., *Astragalus mongholicus* Bunge, and *Lonicera japonica* Thunb., have formulated inspection items for the content of heavy metals and harmful elements, whereas Chinese patent medicines have basically not formulated relevant inspection items. Therefore, we should pay attention to the problem of heavy metal pollution in Chinese patent medicines and formulate detection methods for safety indicators such as heavy metals and harmful elements. However, during preparations, it is not completely clear whether there is interaction and whether it is transformed *in vivo* due to the complex composition.

The research and development of traditional Chinese medicine injection have become one of the hot spots in the modernization of traditional Chinese medicine. Compared with ordinary preparations, it is a high-risk variety from the perspective of clinical application. Therefore, the research on the safety of traditional Chinese medicine injection has attracted much attention, especially the residue of heavy metals and harmful elements. The residues of heavy metals and harmful elements mainly come from environmental pollution, processing, storage, and migration of packaging containers such as ampoules. At present, the determination of elements in injections derived from traditional Chinese Medicine usually adopts the single element method, which has complicated procedures and a long analysis time. The ICP-MS method can simultaneously determine Pb, As, Cd, Hg, Cu, and other elements in injections derived from traditional Chinese Medicine and has become an important means of element analysis in recent years, as shown in Table 10. In terms of clinical medicine, injections derived from traditional Chinese medicine are high-risk varieties; Pb, Cd, As, Hg, and Cu, represented by heavy metals, are among the most important exogenous pollutants in injections derived from traditional Chinese medicine. Toxicity is mainly characterized by chronic poisoning, As, Cd element with clear carcinogenic, teratogenic, and mutagenic effects, and injections derived from traditional Chinese medicines without digestive tract directly into the bloodstream. Therefore, it is significant to carry out limited inspection of heavy metals and harmful elements in injections derived from traditional Chinese Medicine. Although the country has not made a clear limit on trace elements of Fe, Mn, Zn, Al, and others, their excessive amount may also cause adverse effects on the human body. For injections derived from traditional Chinese medicine, the complexity of the ingredients also determines the diversity of their efficacy, making it difficult to distinguish between effective ingredients and toxic ingredients. As an important component of the material basis of drug properties, trace elements are closely related to the drug properties, efficacy and adverse drug reactions. Therefore, accurate determination of trace elements is of great significance to the study of pharmacodynamics, the safety of drug intake, and the formulation of the limit standard of harmful elements.

**TABLE 10:** Application of ICP-MS in the determination of heavy metal elements in Chinese patent medicine.

Name	Medical system	The Main medicinal materials	Therapeutic use	Main pharmacological effects	Test results
*Astragalus mongholicus* injections	Traditional Chinese medicine	*Astragalus mongholicus* Bunge	Viral myocarditis with blood stasis, cardiac insufficiency, hepatitis	Reducing blood sugar, blood lipid, protecting liver, and anti-tumor	The contents of Pb, As, Cu, Cd, and Hg in *Astragalus mongholicus* injections were determined. The correlation coefficient of standard curve *r* > 0.997, the recovery was 92.6%–100.5%, RSD < 2.9% (Shi et al., 2013)
Banxia syrup	Traditional Chinese medicine	*Aster tataricus* L.f., *Citrus deliciosa* Ten., *Ephedra equisetina* Bunge, *Glycyrrhiza uralensis* Fisch. ex DC., *Pinellia ternata* (Thunb.) Makino, *Platycodon grandiflorus* (Jacq.) A.DC., *Polygala tenuifolia* Willd., *Rhaphiolepis bibas* (Lour.) Galasso & Banfi	Cough, phlegm, bronchitis	Bacteriostasis	The contents of Pb, Cd, As, Hg, Cu, and Cr in banxia syrup were determined. The linear relationship of each element to be tested is good, *r* ≥ 0.9970, the detection limit is 0.3–15.2 ng·g^−1^, the recovery is 90.4%–98.7%, RSD ≤ 5.8% (Zheng et al., 2018)
Biejiajian pill	Traditional Chinese medicine	*Iris domestica* (L.) Goldblatt & Mabb., *Carapax trionycis* Wiegmann, *Scutellaria baicalensis* Georgi	Chronic hepatitis, liver cirrhosis, angina pectoris, hyperlipidemia	Anti-renal fibrosis and anti-tumor	The contents of Pb, As, Hg, Cr, and Cu in Biejiajian pills were determined. The ratio of mass concentration to ion peak showed a good linear relationship (*r* = 0.9997, 0.9999), and the RSD of precision, stability, and repeatability were less than 2%. The average recovery was 96.7%–100.9%, RSD was < 4% (*n* = 6), and the detection limits were 2.2, 8.0, 0.7, 1.5, and 5.0 μg/kg (Qiu, 2015)
Chapter song bawei aquilaria powder	Tibetan medicine	*Aquilaria sinensis* (Lour.) Spreng., Frankincense, Guangzao, Kapok, *Myristica fragrans* Houtt., *Terminalia chebula* Retz., Travertine, Woody fragrance	Acute and chronic cardiovascular and cerebrovascular diseases, hypertension	Anti hypoxia	The contents of Cr, Co, Cu, Mn, Zn, Mo, Fe, and Ni in chapter song bawei aquilaria powder were determined. The contents of Fe, Mn, Cu, and Ni are high, whereas the contents of other elements are low (Jin et al., 2009)
Danshen Chuanxiong injection	Traditional Chinese medicine	Ligustrazine hydrochloride, *Salvia miltiorrhiza* Bunge	Angina pectoris, myocardial infarction, ischemic stroke, thromboangiitis obliterans	Anti-blood stasis	The contents of Pb, Cd, As, Hg, Cu, Cr, Mn, and Ni in six batches of Danshen Chuanxiong injection were determined. The linear relationship between the eight elements and the peak area is good in their linear range, R ≥ 0.9990, the detection limit is 0.0099–0.2199 ng·mL^−1^, the average recovery of each element is 86.90–104.8%, and the RSD is less than 5% (Cheng et al., 2018)
Dejunmang juequemao	Tibetan medicine	Bear bile, Calculus bovis, Coralline, *Crocus sativus* L., iron gray, Lapis lazuli, Musk, Zogta	Nausea, vomiting, diarrhea, abdominal pain, peptic ulcer, food poisoning	Clearing away heat and toxic material	The contents of 20 elements of Na, Mg, Al, K, Ca, V, Cr, Mn, Fe, Ni, Cu, Zn, As, Se, Mo, Ag, Cd, Au, Hg, and Pb in Tibetan medicine Dejunmangjuequemao were determined. The detection limit of each element is 0.0033–2.418 μg·g^−1^, and the recovery is 87.27–106.5% (Rezeng et al., 2016a)
Ermiao pills	Traditional Chinese medicine	*Atractylodes lancea* (Thunb.) DC., *Phellodendron amurense* Rupr.	Foot and knee swelling and pain, flaccidity syndrome, wet sore, eczema, erysipelas, leucorrhea	Anticonvulsant, antibacterial, antihypertensive, hypoglycemic	The residues of Pb, Cd, As, Hg, and Cu in Ermiao pills were determined. The RSD values were 1.1%, 0.3%, 0.8%, 0.5%, and 0.8%, all less than 2% (Zhang et al., 2013)
Fufang Danshen tablets	Traditional Chinese medicine	Borneol, *Panax notoginseng* (Burkill) F. H. Chen, *Salvia miltiorrhiza* Bunge	Coronary heart disease, angina pectoris, Alzheimer’s disease, diabetes mellitus, and its complications	Anti-cerebral ischemia, anti-Alzheimer’s disease, anti-osteoporosis	The contents of Pb, As, Cu, Cd, and Hg in Fufang Danshen tablets were determined. The minimum detection limits of five elements were 6.5 ng·g^−1^, 2.3 ng·g^−1^, 5.9 ng·g^−1^, 4.7 ng·g^−1^ and 0.91 ng·g^−1^, respectively. The linear relationship was good in each concentration range (*r* > 0.9980). The recoveries were 102.8%, 105.1%, 104.5%, 106.8%, 92.4%, and RSD were 2.1% and 2.5%, respectively 8%, 1.9%, 2.2%, 3.4% (*n* = 9) (Wu, 2016)
Fufang Dilong capsule	Traditional Chinese medicine	*Achyranthes bidentata* Blume, *Astragalus mongholicus* Bunge, *Conioselinum anthriscoides* “Chuanxiong,” *Pheretima asiatica* Perrier.	Syndrome of qi deficiency and blood stasis in the recovery period of meridians in ischemic stroke	Anti-cerebral ischemia	The contents of Pb, Cd, As, Hg, and Cu in the Fufang Dilong capsule were determined. The linear relationship between the determined elements is good (*r* > 0.9995), and the recovery is between 98.8% and 105.4% (Zou, 2016)
Ganmao Qingre granules	Traditional Chinese medicine	*Platycodon grandiflorus* (Jacq.) A.DC., *Pueraria edulis* Pamp.	Cold, fever, headache	Antiviral, antibacterial, and anti-inflammatory	The contents of 14 common metal elements of Mg, Cr, Fe, Mn, Ca, Zn, As, Se, Ni, Cu, Sb, Cd, Ba, and Pb in Ganmao Qingre granules were determined. The correlation coefficient *r* > 0.9991, the recovery was 80.59%–102.43%, and the relative standard deviation RSD was 0.35%–2.74% (Liu, 2017)
Guzhecuoshang capsules	Traditional Chinese medicine	*Angelica sinensis* (Oliv.) Diels, *Boswellia sacra* Flück., *Carthamus tinctorius* L., Cucumber-seed, *Eupolyphaga sinensis* Walker, Myrrh, Natural copper, Pig bone, *Rheum tanguticum* (Maxim. ex Regel) Balf.	Traumatic injury, detumescence and blood stasis, twisting waist, and bifurcating Qi	Promoting bone healing	The contents of Pb, Cd, Cu, As, and Hg in the fracture contusion capsule were determined. The mass concentrations of the five elements have a good linear relationship with the response intensity in the linear range. The average recovery is 97.4%–102.7%, and the RSD is less than 3.5% (*n* = 6) (Kong et al., 2020)
Guipi pills	Traditional Chinese medicine	*Angelica sinensis* (Oliv.) Diels, *Astragalus mongholicus* Bunge, *Atractylodes macrocephala* Koidz., *Codonopsis pilosula* (Franch.) Nannf., *Dimocarpus longan* Lour. *Glycyrrhiza uralensis* Fisch. ex DC., *Poria cocos* (Schw.) Wolf., *Polygala tenuifolia* Willd., *Ziziphus jujuba* Mill.	Deficiency of both heart and spleen, Palpitation and palpitation caused by spleen not regulating blood	Anti-shock, regulating central nerve, enhancing immune and hematopoietic function	The contents of Cu, Pb, Hg, As, and Cd in Guipi pills were determined. The correlation coefficients of the measured elements and calibration curves are 0.9999, and the linear relationship is good. The average recovery of the method is 90.3%–103.6%, and the RSD is 1.6%–3.9% (Zhu et al., 2017)
Guizhi Fuling capsules	Traditional Chinese medicine	*Cinnamomum verum* J.Presl, *Paeonia lactiflora* Pall., *Paeonia* × *suffruticosa* Andrews, *Poria cocos* (Schw.) Wolf., *Prunus persica* (L.) Batsch	Ovarian cyst, endometriosis, hysteromyoma, dysmenorrhea	Anti-inflammatory, analgesic, anti-tumor, regulating smooth muscle, regulating endocrine, and improving immunity	1) The contents of Pb, Cu, As, Cd, Hg, Mg, Mn, Ni, and Tl in Guizhi Fuling Capsule and five medicinal materials of *Cinnamomum verum* J.Presl, *Poria cocos* (Schw.) Wolf., *Paeonia lactiflora* Pall., *Prunus persica* (L.) Batsch, *Paeonia* × *suffruticosa* Andrews were determined. The linear relationship is good, and the correlation coefficient *R* ^2^ ≥ 0.9993. The detection limit of the method is 0.016–4.593 μg·L^−1^, and the experimental repeatability is good. The recovery of the sample was 75.84%–118.9% (Wang et al., 2014b)
					2) The contents of 29 inorganic elements of Li, Be, B, Na, Mg, Al, Ca, Ti, V, Cr, Mn, Fe, Co, Ni, Cu, Zn, Ga, As, Se, Sr, Mo, Cd, Sn, Sb, Ba, Hg, Tl, Pb, and Bi in 12 batches of Guizhi Fuling capsules were determined. The contents of Ca, Na, and Mg are high, all exceeding 300 μg/g. The contents of Fe, Mn, and SR are also relatively high, whereas the contents of harmful elements of Be, As, Cd, Sb, Hg, Tl, and Bi are relatively low, all below 0.030 μg/g (Kang et al., 2018)
Hanshuishi Ershiyiwei powder	Mongolian medicine of China	Cow bezoar, *Forsythia suspensa* (Thunb.) Vahl, Hanshuishi, *Hippophae rhamnoides* L., *Momordica cochinchinensis* (Lour.) Spreng., *Punica granatum* L., Trogopterus dung, *Viola philippica* Cav.*Wurfbainia villosa* (Lour.) Skornick. & A. D. Poulsen	Chest and back pain, Qi stagnation and blood stasis, blood heat-trapping stomach	Anti-gastric ulcer	The contents of Pb, Cd, As, Hg, and Cu in Hanshuishi Ershiyiwei Powder were determined. The mass concentrations of the five elements were in the range of 0–100 ng/ml and had a good linear relationship with the response value of the reference peak (*r* > 0.997). The average recovery was 87.90%–97.63%, and the RSD was 1.52%–2.35%. The detection limits of five metal elements are 0.004 4–0.0551 ng/ml (Mao and Chen, 2020)
Honghua injection	Traditional Chinese medicine	*Carthamus tinctorius* L.	Coronary heart disease, occlusive vascular disease	Anti-stress	The contents of Cr, Cu, Zn, Mo, Pb, Ba, B, and Al in the Honghua injection were determined. The correlation coefficient of the standard curve of eight elements is 0.9933–0.9999. The average recovery was 90.7%–106.5% (RSD% was 1.2%–4.1%) (Peng et al., 2020)
Licorice oral solution	Traditional Chinese medicine	*Glycyrrhiza uralensis* Fisch. ex DC.	Cough and expectoration caused by upper respiratory tract infection, bronchitis, and cold	Anti-inflammatory	The contents of Cr, Mn, Co, Ni, Cu, Zn, As, Cd, Sn, Sb, Ba, Hg, Pb, Al, and Fe in Licorice oral solution were determined. The linear relationship of 15 elements is good, the correlation coefficient is greater than 0.999, the detection limit of each element is 0.08–38.00 ng·mL^−1^, and the recovery is 88.57%–107.10% (*n* = 6) (Li et al., 2019c)
Manubzhithang	Tibetan medicine	*Inula helenium* L., *Rubus biflorus* Buch.-Ham. ex Sm., *Tinospora sinensis* (Lour.) Merr., *Zingiber officinale* Roscoe	In the early stage of influenza, chills, headache, arthralgia, rheumatoid arthritis, and fever	Antibacterial, analgesic, anti-inflammatory, and anti-allergic	The contents of Cr, Mn, Ni, Co, Cu, Zn, and As in Manubzhithang were determined. The detection limit of each element is 0.003–0.095 μg·g^−1^, and the recovery is 90%–105% (Rezeng et al., 2010)
Montmorillonite Powder	Traditional Chinese medicine	Montmorillonite	Chronic diarrhea	Cover the mucous membrane of the digestive tract and bind with mucus protein	The contents of Pb, Cd, Hg, Co, V, Ni, and As in montmorillonite powder were determined. The seven heavy metal elements have a good linear relationship at 0–30 ng/ml, 0–1 ng/ml, 0–6 ng/ml, 0–10 ng/ml, 0–20 ng/ml, 0–40 ng/ml, and 0–4 ng/ml, respectively. The RSD of precision were 4.91%, 5.39%, 10.00%, 4.78%, 5.93%, 4.67%, and 6.91%, respectively. The RSD of stability were 5.64%, 5.12%, 10.94%, 3.68%, 4.97%, 4.31%, and 7.06% respectively (Huang et al., 2019)
Niuhuang Qingwei pills	Traditional Chinese medicine	Calculus bovis, *Citrus* × *aurantiifolia* (Christm.) Swingle, *Gardenia jasminoides* J.Ellis, *Glycyrrhiza uralensis* Fisch. ex DC., *Mentha canadensis* L., *Ophiopogon japonicus* (Thunb.) Ker Gawl., *Phellodendron chinense* C. K. Schneid., *Rheum palmatum* L., *Scutellaria baicalensis* Georgi, *Scrophularia ningpoensis* Hemsl.	Heartburn, dizziness, sore tongue, swollen gums, sore throat, constipation	Antibacterial, antiviral, and cardiovascular protection	The contents of Pb, Cd, As, Hg, and Cu in Niuhuang Qingwei pills were determined. Cd and Cu did not exceed the standard, and the exceeding rates of Pb, As, and Hg were 12%, 12%, and 14%, respectively (Nie et al., 2019)
Qishiwei Zhenzhu pills	Tibetan medicine	Agate, *Aquilaria malaccensis* Lam., Bezoar, *Bos taurus domesticus Gmelin*, Coral, *Crocus sativus* L., *Dalbergia odorifera* T. C. Chen, Gold, Musk, Myrobalan, Opal, Pearl, *Saiga tatarica* Linnaeus, *Santalum album* L., Silver, Zuotai	“Baimai” disease, stroke, paralysis, hemiplegia, cerebral bemorrhage	Anticonvulsant and hypotensive	Determination of 18 trace elements of Li, Be, Sc, V, Cr, Mn, Co, Ni, Cu, As, Sr, Ag, Cd, Cs, Ba, Pb, Au, and Hg in Qishiwei Zhenzhu pills. The rank order of the elements in Qishiwei Zhenzhu pills was copper > mercury > lead from high to low, with the mass fraction higher than 6,000 μg/kg. The mass fractions of argentum, arsenic, manganese, aurum, strontium, barium, chromium, and nickel were in the range of 33–1,034 μg/kg. The mass fractions of vanadium, cobalt, lithium, beryllium, cadmium, scandium, and cesium were lower than 10 μg/kg (Fu et al., 2022)
Qiangli Pipa syrup	Traditional Chinese medicine	Menthol, Morus alba, *Papaver somniferum* L., *Platycodon grandiflorus* (Jacq.) A.DC., *Rhaphiolepis bibas* (Lour.) Galasso & Banfi, *Stemona sessilifolia* (Miq.) Miq.	Bronchitis, cough	Anti-inflammatory, hypoglycemic, antiviral, and anti-tumor	The contents of 20 inorganic elements of Li, Be, V, Mn, Co, Ni, Cu, Zn, Ga, As, Se, Rb, Sr, Cd, Sn, Sb, Cs, Ba, Tl, and Pb in Qiangli Pipa syrup were determined. The average recovery was 86.2%–104.9%, the corresponding RSD was 0.55%–9.21%, and the detection limit was 0.01–5.94 ng/ml. The average contents of Sr, Mn, Ba, Rb, and Zn in 19 batches of Qiangli Pipa syrup were the highest (Lu et al., 2020)
Qinghuo Chimai tablets and capsules	Traditional Chinese medicine	*Andrographis paniculata* (Burm.f.) Nees, *Gardenia jasminoides* J.Ellis, *Ophiopogon japonicus* (Thunb.) Ker Gawl.	Sore throat, fever, toothache	Anti-inflammation and analgesia	The contents of Li, V, Cr, Fe, Ni, Co, Cu, Zn, As, Se, Mo, Ru, Rh, Pd, Ag, Cd, Sn, Sb, Ba, Ir, Pt, Au, Hg, Tl, and Pb in Qinghuo Zhimai tablets and capsules were determined. The linear relationship of each element in their respective concentration range is good (*r* > 0.999). The lower detection limit is 0.0003–5.2 μg·d^−1^. The recovery was 88.1%–121.9%, and RSD was 0.7%–6.7% (Wang et al., 2019b)
Qingxue Bawei tablets	Mongolian medicine of China	Bezoar, *Dianthus superbus* L., *Gardenia jasminoides* J.Ellis, *Glycyrrhiza uralensis* Fisch. ex DC., Hansuishi, *Inula helenium* L., *Lithospermum erythrorhizon* Siebold & Zucc., Plaster	Headache, heatstroke	Protecting cardiovascular system, anti-arrhythmia, anti-inflammatory, and protecting myocardium	The contents of Be, B, Na, Mg, Al, P, K, Ca, Ti, V, Cr, Mn, Fe, Co, Ni, Cu, Zn, Ga, As, Se, Rb, Sr, Mo, Ag, Cd, Sn, Sb, Cs, Ba, Hg, Tl, and Pb in Qingxue Bawei tablets were determined. The linear relationship of each element is good, *r* ≥ 0.9995, and the detection limit of each element is 0.001–6.390 μg/L; RSD of the precision, stability, and repeatability tests meet the requirements of quantitative analysis. The recovery was 94.36%–105.47%, and RSD was 1.53%–4.56% (Wang et al., 2017b)
Renshenzaizao pills	Traditional Chinese medicine	*Agkistrodon acutus* (Guenther), *Asarum sieboldii* Miq., *Cyperus rotundus* L., *Panax ginseng* C.A.Mey., *Pheretima asiatica* Perrier., *Pogostemon cablin* (Blanco) Benth., *Scrophularia ningpoensis* Hemsl.	Apoplexy	Anti-arrhythmia and anti-shock	The contents of Pb, Cd, As, Hg, and Cu in Renshenzaizao pills were determined. The linear relationship of the five elements is good, the correlation coefficient is greater than 0.9990, the detection limit is 0.0002–0.023 mg/kg, and the average recovery is 92.7%–101.9% (Zhang et al., 2019b)
Renqing Changjue	Tibetan medicine	*Aquilaria sinensis* (Lour.) Spreng., Bezoar, Cinnabar, *Crocus sativus* L., *Santalum album* L., Pearl, Zogta	Harmonizing and nourishing, clearing away heat and detoxifying	Analgesia and anti-fatigue	The contents of Na, Mg, Al, K, Ca, V, Cr, Mn, Fe, Co, Ni, Cu, Zn, As, Se, Mo, Ag, Cd, Au, Hg, Tl, Pb, Th, and U in Renqing Changjue were determined. The detection limit of each element is 0.0032–2.416 ng·g^−1^, and the recovery is between 82.73% and 106.6% (Rezeng et al., 2016b)
Salvia miltiorrhiza freeze-dried powder needle for injection	Traditional Chinese medicine	*Salvia miltiorrhiza* Bunge	Chest tingling, palpitation, coronary heart disease, angina pectoris	Antiviral, anti-tumor, cellular immunity, and interferon induction	The contents of Pb, Cd, As, Hg, and Cu in 32 batches of freeze-dried powder needles of Salvia miltiorrhiza for injection and Xueshuantong for injection were determined. The correlation coefficient *r* of the measured elements and calibration curve is greater than 0.999. The contents of Pb, Cd, As, Hg, and Cu in 32 batches of freeze-dried powder injection of traditional Chinese medicine for injection are within the limit (Jiang et al., 2017)
Xueshuantong freeze-dried powder needle for injection	Traditional Chinese medicine	*Panax notoginseng* (Burkill) F. H. Chen	Stroke hemiplegia, chest arthralgia and heartache, central retinal vein occlusion	Expand cerebral blood vessels and increase cerebral blood flow	
Sanqi Shangyao tablets	Traditional Chinese medicine	*Aconitum brachypodum* Diels, Borneol, *Carthamus tinctorius* L., *Davallia mariesii* H.J.Veitch, *Paeonia veitchii*, *Panax notoginseng* (Burkill) F. H. Chen, *Sambucus williamsii* Hance	Relaxing tendons and activating blood circulation, dispersing blood stasis, and relieving pain	Anti-inflammatory and anticoagulant	The contents of Pb, Cd, As, Hg, and Cu in Sanqi Shangyao tablets were determined. The detection limits were 0.02 mg/kg, 0.01 mg/kg, 0.005 mg/kg, 0.03 mg/kg, and 0.007 mg/kg, respectively (Ma et al., 2019)
Shexiang Baoxin pills	Traditional Chinese medicine	Bezoar, Borneol, *Bufo bufo gargarizans*, *Cinnamomum verum* J.Presl, *Liquidambar orientalis* Mill., Musk, *Panax ginseng* C.A.Mey.	Angina pectoris and myocardial infarction caused by myocardial ischemia	Inhibiting myocardial fibrosis and reducing cardiac inflammation	The contents of 26 inorganic elements of Li, Na, Mg, Al, Ca, Ti, V, Cr, Mn, Fe, Co, Ni, Cu, Zn, Ga, As, Se, Sr, Mo, Cd, Sb, Ba, Hg, Tl, Pb, and Bi in 12 batches of Shexiang Baoxin pills were determined. The contents of Ca, Mg, Na, and Fe are higher, all exceeding 1.000 mg/g. The amounts of Mn, Cu, Zn, Al, and Ga are also relatively high, whereas the amounts of Sb, Hg, Tl, and Bi are relatively low, all below 0.050 μg/g (Ding et al., 2018)
Shenqifuzheng injection	Traditional Chinese medicine	*Astragalus mongholicus* Bunge, *Codonopsis pilosula* (Franch.) Nannf.	Lung cancer, gastric cancer	Anti-tumor	The contents of 14 trace elements Cu, Zn, Mn, Se, Mo, Fe, Cr, Ni, V, Sn, Pb, Cd, Hg, and As in q Shenqifuzheng injection were determined. The relative contents of Cu, Fe, Zn, Cr, and Se in Shenqifuzheng injection are high. The contents of As in toxic elements Pb, Cd, Hg, and as are close to the requirements of American fad drugs and functional foods, and the contents of Pb, Cd, and Hg are significantly lower than the maximum limit (Shi et al., 2008)
Shengmai injections	Traditional Chinese medicine	*Ophiopogon japonicus* (Thunb.) Ker Gawl., *Panax ginseng* C. A. Mey., *Schisandra chinensis* (Turcz.) Baill.	Myocardial infarction, cardiogenic shock, septic shock	Improving body immunity, protecting myocardium, and anti-tumor	The contents of heavy metal elements Cu, As, Cd, Hg, and Pb in Shengmai injection were determined. The linear relationship of each element in their respective detection mass concentration range is good (*r* is not less than 0.9998), the recovery is greater than 90%, RSD is not greater than 2.8% (*n* = 3), and the detection limits are Cu 0.10 ng/ml, As 0.06 ng/ml, Cd 0.03 ng/ml, Hg 0.008 ng/ml and Pb 0.02 ng/ml, respectively (Huang, 2014)
Shiwuwei Saierdou pill	Tibetan medicine	*Berberis thunbergii* DC., *Chrysosplenium nudicaule* Bunge, *Hypecoum erectum* L., *Lagotis clarkei* Hook.f., *Swertia bimaculata* (Siebold & Zucc.) Hook.f. & Thomson ex C. B. Clarke, *Terminalia chebula* Retz., *Dolomiaea souliei* (Franch.) C. Shih	Hepatobiliary fever, cholecystitis, cholelithiasis, choledocholithiasis	Anti-inflammation, analgesia, and bacteriostasis	The contents of 20 elements Na, Mg, Al, K, Ca, V, Cr, Mn, Fe, Co, Ni, Cu, Zn, As, Se, Mo, Ag, Au, Hg, and Pb in Shiwuwei Saierdou pills were determined. The detection limit of each element is 0.0032–2.416 ng·g^−1^, and the recovery is 86.92%–104.9% (Rezeng et al., 2016c)
Shuanghuanglian injection	Traditional Chinese medicine	*Forsythia suspensa* (Thunb.) Vahl, *Lonicera japonica* Thunb., *Scutellaria baicalensis* Georgi	Fever, cough, sore throat, upper respiratory tract infection, mild pneumonia, and tonsillitis caused by exogenous wind heat	Antibacterial, anti-inflammatory, antiviral, and antiarrhythmic	The contents of 15 trace elements Cr, Mn, Fe, Co, Ni, Cu, Zn, As, Cd, Ba, and Hg in Shuanghuanglian injection were determined. The recovery was 81.6%–96.3%, and the detection limit was 0.001–1.53 ng/g (Luan et al., 2012)
Sijunzi decoction	Traditional Chinese medicine	*Atractylodes macrocephala* Koidz., *Glycyrrhiza uralensis* Fisch. ex DC., *Panax ginseng* C. A. Mey., *Poria cocos* (Schw.) Wolf.	Anti-tumor, anti-ribonucleic acid synthesis, gastrointestinal regulation	Anti-tumor, anti-fatigue, and anti-aging	The contents of Pb, Hg, Cr, Cd, As, Ni, Cu, Co, Se, and Mo in Sijunzi decoction were determined. The linear relationship of the detected 10 elements is good, the correlation coefficient *r* ≥ 0.9993, and the detection limit of each element is 0.003–0.294 μg·L^−1^, the recovery is within 95.8%–108.3%, RSD ≤ 6.6%, and values of repeatability and precision are RSD ≤ 5% (Guan et al., 2018)
Thrombus scavenger injection	Traditional Chinese medicine	*Panax notoginseng* (Burkill) F. H. Chen	Hemorrhagic diseases, cerebrovascular diseases and their sequelae, intraocular hemorrhage, and other fundus diseases	Expand cerebral blood vessels and increase cerebral blood flow	The contents of Be, Al, Cr, Mn, Fe, Co, Ni, Cu, Zn, As, Se, Ag, Cd, Sn, Sb, Ba, Nd, Dy, Hg, Tl, and Pb in Thrombus scavenger injection were determined. The correlation coefficient *r* of the standard curve of 21 elements above 0.999, the recovery rate is 92 71%–107.07%, and RSD is within 5% (Cheng et al., 2019)
Twenty-five flavor pearl pills	Tibetan medicine	Aromaticum, Buffalo horn, *Crocus sativus* L., *Dalbergia odorifera* T. C. Chen, *Myristica fragrans* Houtt., *Lanxangia tsao-ko* (Crevost & Lemarié) M. F. Newman & Skornick*.*, Pearl, *Phyllanthus emblica* L., *Santalum album* L., Travertine	Acute and chronic cardiovascular and cerebrovascular diseases, hypertension, hyperlipidemia	Anticonvulsant and antithrombotic	The contents of eight trace elements Fe, Cu, Mn, Cr, Ni, Zn, Co, and Mo in Tibetan medicine, twenty-five flavor pearl pills, were determined. The recovery was 95.4%–102.3% (Jin et al., 2017)
Vitexin injection	Traditional Chinese medicine	*Crataegus pinnatifida* Bunge, *Vitex negundo* L.	Inflammation, ischemic brain injury	Antioxidation, anti-myocardial hypertrophy, and inhibition of platelet aggregation	The contents of Pb, Cd, As, Hg, and Cu in Vitexin injection were determined. The detection limits of each element were 0.4, 0.03, 0.09, 0.04, and 0.05 ng/ml, respectively. The linear relationship is good (*r* > 0.998). The recoveries were 97.8%, 97.2%, 104.0%, 91.2%, and 92.8%, respectively (Xu et al., 2018)
Weisu granule	Traditional Chinese medicine	*Areca catechu* L., *Cyperus rotundus* L., *Citrus deliciosa* Ten., *Citrus medica* L., *Citrus* × *aurantiifolia* (Christm.) Swingle, Gizzard pepsin, *Perilla frutescens* (L.) Britton	Chronic gastritis, Peptic ulcer	Improving the state of gastric mucosa and reducing the expression level of inflammatory factors	The six harmful elements Pb, Cd, As, Hg, Cu, and Ni in Weisu granule were determined. Within the detection concentration range, the linear relationship is good (*r* value is between 0.9993 and 0.9999), the recovery of each element is between 92.36% and 102.55%, and the RSD is between 0.85% and 2.1% (Su et al., 2020)
Xiaochaihu preparation	Traditional Chinese medicine	*Bupleurum chinense* DC., *Glycyrrhiza uralensis* Fisch. ex DC., *Panax ginseng* C.A.Mey., *Pinellia ternata* (Thunb.) Makino, *Scutellaria baicalensis* Georgi	Cold, malaria, chronic hepatitis, liver cirrhosis, acute and chronic cholecystitis, otitis media, acute pancreatitis, pleurisy, gallstones	Hypolipidemia	The contents of Cu, As, Cd, Hg, and Pb in Xiaochaihu preparation were determined. Under the international heavy metal limit standard, Hg in only one batch of original Medicinal materials exceeded the standard by eight times, but after decoction and preparation process, the residue of heavy metals was significantly reduced, and the residue rate of heavy metals was reduced to 2.5% 02% (granule Hg)–42.85% (granule Cd). THQ and Cr were lower than the non-carcinogenic and carcinogenic risk standard values of heavy metals under the three-drug application methods (Li et al., 2019d)
Yigan powder	Traditional Chinese medicine	*Artemisia capillaris* Thunb., *Astragalus mongholicus* Bunge, *Curcuma aromatica* Salisb., *Phyllanthus urinaria* L., *Salvia miltiorrhiza* Bunge, *Strobilanthes cusia* (Nees) Kuntze	Chronic hepatitis B, hepatitis C, cirrhosis, fatty liver, alcoholic liver disease, hepatic cyst	Anti-hepatitis B virus	The contents of V, Cr, Fe, Co, Ni, Cu, As, Se, Mo, Cd, Hg, and Pb in Yigan powder were determined. The linear relationship of 12 elements is good, and the correlation coefficient *r* ≥ 0.05 9992, the detection limit of each element is 0.038–0.537 μg·L^−1^, the recovery is 100.0%–104.7%, RSD ≤ 4.4%, and RSD values of repeatability and precision are ≤5% (Sun et al., 2017)
Yinzhihuang injection	Traditional Chinese medicine	*Artemisia capillaris* Thunb., *Gardenia jasminoides* J.Ellis, *Lonicera japonica* Thunb., *Scutellaria baicalensis* Georgi	Acute, persistent, and chronic hepatitis	Protecting the liver and resisting liver fibrosis	Determination of Pb, As, Cd, Hg, and Cu in Yinzhihuang injection. The detection limits of the five elements were 5–1250 ng/L, respectively. The linearity is good, and the linear correlation coefficients are *r* ≥ 0.999. Precision RSD < 3 5%. The recovery was 95.7%–107.5% (Wang et al., 2012b)
Yupingfeng granules	Traditional Chinese medicine	*Astragalus mongholicus* Bunge, *Atractylodes macrocephala* Koidz., *Saposhnikovia divaricata* (Turcz. ex Ledeb.) Schischk.	Cold, respiratory tract infection, allergic rhinitis	Anti-infection	18 inorganic elements K, Ca, Ti, V, Cr, Mn, Fe, Co, Ni, Cu, Zn, Se, Sr, Mo, Cd, Sn, Sb, and Ba in Yupingfeng granules were determined. The precision, stability, and reproducibility are less than 5%, 18 inorganic elements are in the range of 0–100 g/L, and the correlation coefficients were above 0.9990 (Zhang et al., 2019c)
Zhenqi Fuzheng granules	Traditional Chinese medicine	*Astragalus mongholicus* Bunge, *Ligustrum lucidum* W. T. Aiton	Deficiency caused by various diseases	Anti-fatigue and anti-oxidation	The contents of As, Cd, Pb, Hg, and Cu in 111 batches of Zhenqi Fuzheng granules were determined. The correlation coefficient of the calibration curve *r* > 0.999, and the contents of As, Cd, Pb, Hg, and Cu are within the controllable range (Zhou et al., 2016)
Zhenhuang capsule	Traditional Chinese medicine	Artificial bezoar, Borneol, *Panax notoginseng* (Burkill) F.H.Chen, Pig bile powder, Pearl, Peppermint oil, *Scutellaria baicalensis* Georgi	Sore throat, sore and boils caused by excessive heat in lung and stomach	Anticonvulsant	The contents of Pb, Hg, Cd, Cu, and As in Zhenhuang capsule were determined. The detection limit of each element is 1.1–14.0 ng·mL^−1^. The quantitative limit was 3.7–46.7 ng·mL^−1^. The linear ranges are Pb 0–20 ng·mL^−1^, As 0–20 ng·mL^−1^, Hg 0–2 ng·mL^−1^, Cd 0–10 ng·mL^−1^, and Cu 0–500 ng·mL^−1^, respectively. *r* > 0.9900, the linear relationship of each element is good. The average recovery was 92.1%–106.5%, RSD ≤ 5.72% (Liu et al., 2020)
Zhitong Huazheng capsule	Traditional Chinese medicine	*Angelica sinensis* (Oliv.) Diels, *Astragalus mongholicus* Bunge, *Atractylodes macrocephala* Koidz., *Codonopsis pilosula* (Franch.) Nannf., *Curcuma phaeocaulis* Valeton, *Sparganium stoloniferum* (Buch.-Ham. ex Graebn.) Buch.-Ham. ex Juz., *Spatholobus suberectus* Dunn, *Salvia miltiorrhiza* Bunge	Irregular menstruation, dysmenorrhea, chronic pelvic inflammation	Antithrombosis	48 inorganic elements Li, Be, V, Cr, Mn, Co, Ni, Cu, Zn, Ga, As, Se, Rb, Sr, Cd, Sn, Sb, Cs, Ba, Hg, Pb, Bi, B, Sc, Ti, Ge, Y, Zr, Nb, Mo, Ag, La, Ce, Pr, Nd, Sm, W, Eu, Gd, Dy, TB, Ho, Er, Tm, Yb, Ta, and Tl in Zhitong Huazheng capsule were determined, and the regression equation coefficient of each element was greater than 0.9994. The detection limit of each element is 0.0001–0.1611 g/L. The quantitative limit is 0.0003–0.5370 g/L, the RSD of the precision, stability, and repeatability test is less than 5.0%, the recovery rate is 95.0%–101.7%, and the RSD value is 0.34%–2.83% (Wu et al., 2019b)
Zhusha Anshen pills	Traditional Chinese medicine	*Angelica sinensis* (Oliv.) Diels, Cinnabar, *Coptis chinensis* Franch., *Glycyrrhiza uralensis* Fisch. ex DC., *Reynoutria multiflora* (Thunb.) Moldenke	Insomnia, palpitation, and forgetfulness caused by neurasthenia, trance caused by mental depression, palpitation and palpitation caused by premature heartbeat	Anticonvulsant	To determine the content of soluble mercury in Zhusha Anshen pills. There is a good linear relationship in the range of 0–20.0 g/L. The detection limit is 0.04 g/L, and the limit of quantitation is 0.13 g/L. The average recoveries of the three mass concentrations were 94.7%, 110.4%, and 92.3%. RSD was 1.3%, 1.0%, and 3.6% (Li et al., 2016b)

**FIGURE 5:**
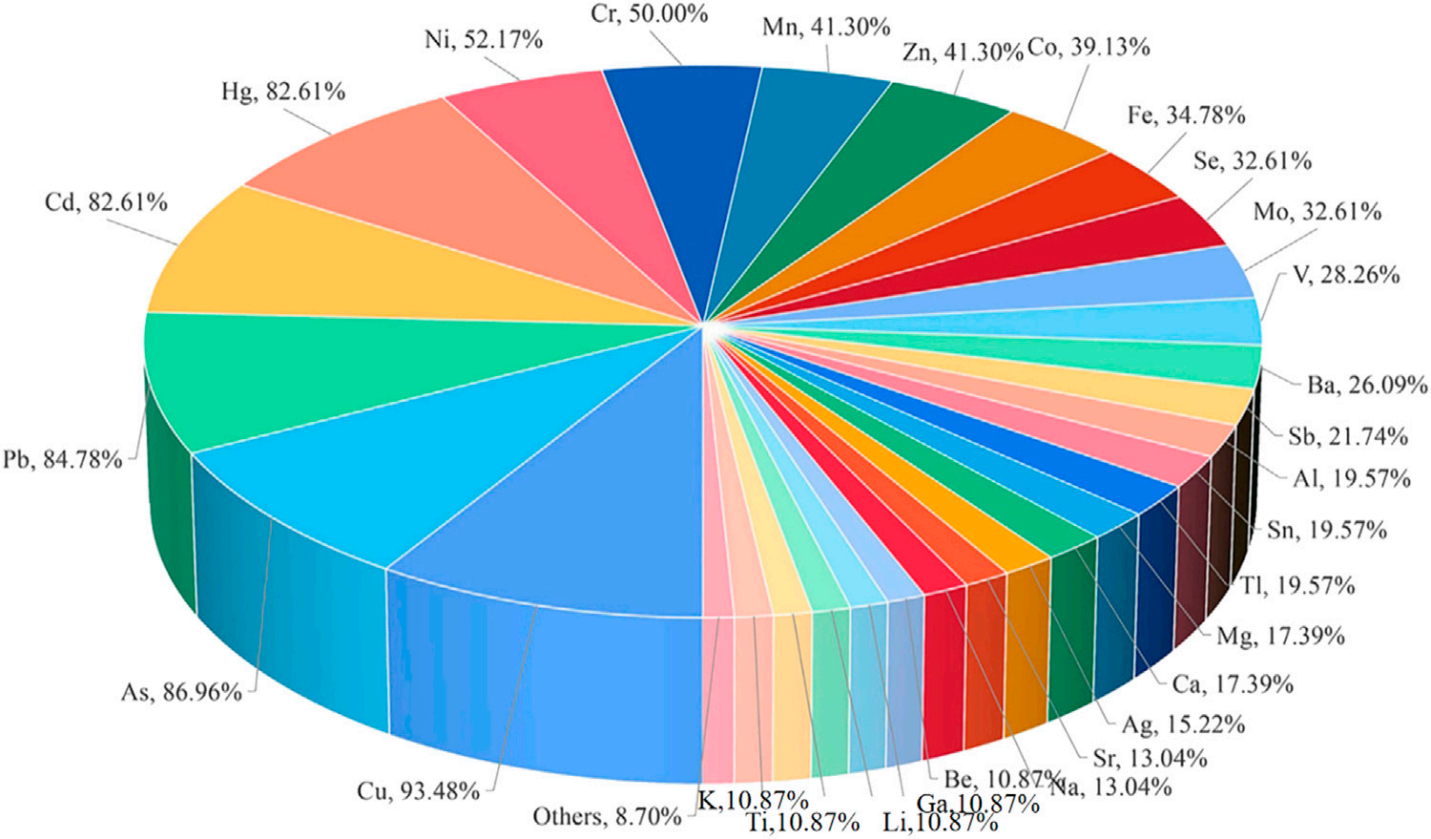
Pie chart of elements measured by ICP-MS and their proportions in Chinese patent medicine.

From the 46 articles on Chinese patent medicine, 62 elements such as Cu, As, Pb, Cd, Hg, Ni, and Cr were determined, of which 43 were Cu, accounting for 93.48%. Thirty-eight of the tested Chinese patent medicine belong to traditional Chinese medicine, accounting for 83%. The elements determined by ICP-MS and their proportion in Chinese patent medicine are shown in Figure 5. Six articles belong to Tibetan medicine, accounting for 13%. Two articles belong to the medical system of Mongolian medicine in China, accounting for 4%.

## Development Status of ICP-MS Combined Technology

**FIGURE 6:**
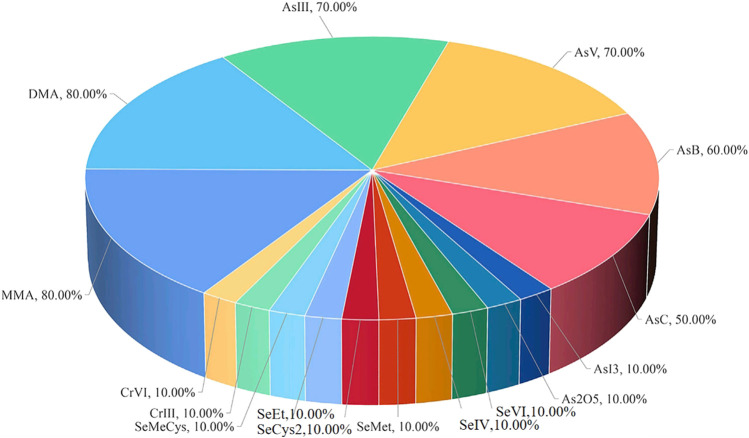
Determination of element content and proportion pie chattachment.art in medical materials and Chinese patient medicines by ICP-MS.

ICP-MS is undoubtedly a very practical means for the quantitative analysis of heavy metals. However, for traditional pharmaceutical preparations, a series of changes will occur when they are absorbed into the human body, such as the valence changes of heavy metals. At this time, it is necessary to accurately detect some valence changes, so the single ICP-MS method becomes weak and needs to be used in conjunction with other instruments to complete this work. At present, ICP-MS has been used in various analytical fields. For example, Heitland et al. (2017) used HPLC-ICP-MS to diagnose and monitor patients with severe hexavalent chromium and inorganic arsenic poisoning to provide valuable data for doctors and toxicologists. However, for the research of traditional drugs, continuous use technology is not widely used. The application of HPLC-ICP-MS in traditional drugs and their preparations are shown in Tables 11 and 12. Other combined analytical techniques are also widely used. However, they are rarely used in traditional medicine. In the searched articles, HPLC-ICP-MS is mostly used to determine different forms of As, including AsC, AsB, AsIII, DMA, MMA, and AsV, and the determination of other elements is less in the articles search. Therefore, more combined techniques are used to analyze traditional medicine. It will be more beneficial to accelerate the development of traditional medicine. From the 10 articles on the application of the combined technique, 16 elements such as MMA, DMA, AsⅢ, AsⅤ, AsB, AsC, and AsI_3_ were determined. Among them, MMA and DMA were determined most, with eight articles accounting for 80.00%. The content and proportion of elements in medicinal materials and Chinese patent medicines determined by ICP-MS are shown in Figure 6. Among the 10 articles on the application of combined technology, the tested medicinal materials and preparations belong to traditional Chinese medicine.

**TABLE 11:** Application of HPLC-ICP-MS in medicinal materials.

Latin name	Medical system	Therapeutic use	Main pharmacological effects	Origin of medicinal materials^a^	Test results
*Astragalus mongholicus* Bunge	Traditional Chinese medicine	Hypertension	Anti-inflammation, immune regulation, anti-tumor, anti-stress, and liver protection	—	The arsenic speciation contents of AsI_3_, As_2_O_5_, MMA, and DMA were determined before and after processing of *Astragalus mongholicus* Bunge, *Rheum tanguticum* (Maxim. ex Regel) Balf., *Scutellaria baicalensis* Georgi, *Reynoutria multiflora* (Thunb.) Moldenke, and *Rehmannia glutinosa* (Gaertn.) DC. The RSD of repeatability, stability, and recovery were 1.21%–4.31%, 1.78%–4.97%, 1.19%–5.57%, and 83.15%–118.32%, respectively (Zhang, 2014)
*Reynoutria multiflora* (Thunb.) Moldenke	Traditional Chinese medicine	Dizziness, malaria, constipation	Anti-inflammatory, anti-tumor, and lipid-lowering	—	
*Rehmannia glutinosa* (Gaertn.) DC.	Traditional Chinese medicine	Hematemesis, bleeding, sore throat	Antibacterial, anti-tumor, anti-gastric ulcer, anti-aging	—	
*Rheum tanguticum* (Maxim. ex Regel) Balf.	Traditional Chinese medicine	Hematemesis, bleeding, edema, drenching	Antibacterial, anti-tumor, anti-gastric ulcer, anti-aging	—	
*Scutellaria baicalensis* Georgi	Traditional Chinese medicine	Diarrhea, jaundice, cough	Antibacterial, anti-inflammatory, anti-fatigue, and anti-tumor	—	
*Alisma plantago-aquatica* L.	Traditional Chinese medicine	Adverse urination, diarrhea, dizziness, astringent pain, hyperlipidemia	Anti-tumor, hypoglycemic, hypolipidemic, and hepatoprotective	—	Four different valence arsenic, As (Ⅲ), DMA, MMA, and As (Ⅴ), were determined in *Alisma plantago-aquatica* L., *Lonicera japonica* Thunb., and *Spatholobus suberectus* Dunn.The linear relationship is good at 5-200ug.L^-1^. The average recovery (*n* = 9) was 105.1%–108.4%, and the detection limit was 0.01 mg·kg^−1^. Small amounts of As (Ⅲ) and As (Ⅴ) were detected in *Alisma orientalis* and small amounts of As (Ⅲ), DMA and As (Ⅴ) were detected in *Lonicera japonica* Thunb., whereas As (Ⅲ), DMA, MMA, and As (Ⅴ) were not detected in *Spatholobus suberectus* Dunn (Wang et al., 2015c)
*Lonicera japonica* Thunb.	Traditional Chinese medicine	Heatstroke, multiple infectious diseases	Anti-inflammatory, antibacterial, antiviral, hypolipidemic, and hypoglycemic	—	
*Spatholobus suberectus* Dunn	Traditional Chinese medicine	Irregular menstruation, dysmenorrhea, amenorrhea, numbness, and paralysis	Anti-inflammatory, analgesic, anti-tumor, antiviral, and antidepressant	—	
*Angelica sinensis* (Oliv.) Diels	Traditional Chinese medicine	Irregular menstruation, amenorrhea, dysmenorrhea, constipation	Analgesic, anti-inflammatory, antibacterial, antioxidant, anti-senile dementia	—	The contents of AsC, AsB, AsIII, DMA, MMA, and AsV in *Citrus deliciosa* Ten., *Gardenia jasminoides* J.Ellis, *Magnolia officinalis*, *Ostrea gigas* Tnunb., *Polygonum tinctorium* Ait., *Angelica sinensis* (Oliv.) Diels, *Lonicera japonica* Thunb., *Lycium barbarum* L., *Gentiana scabra* Bunge. were determined. The detection limits of the measured elements were As (Ⅲ) 4.0 μg/kg, As (V) 5.0 μg/kg, MMA 3.0 μg/kg, DMA 2.0 μg/kg, and AsB2.0 μg/kg. The recovery was 88.7%–101%, and the relative standard deviation was 1.02%–3.34% (*n* = 3). The arsenic forms in 10 traditional Chinese medicines were mainly As (V) and organic arsenic (Li, 2018b)
*Citrus deliciosa* Ten.	Traditional Chinese medicine	Epigastric fullness, omitting and diarrhea, cough and phlegm	Anti-liver injury, anti-tumor, and anti-pulmonary fibrosis	—	
*Gardenia jasminoides* J.Ellis	Traditional Chinese medicine	Icteric hepatitis, sprain, hypertension, diabetes mellitus	Antibacterial, anti-tumor, hypoglycemic	—	
*Gentiana scabra* Bunge.	Traditional Chinese medicine	Hypertension, dizziness, liver disease	Anti-inflammation, analgesia, liver protection, and antiviral	—	
*Lonicera japonica* Thunb.	Traditional Chinese medicine	Fever, heatstroke, multiple infectious diseases	Anti-inflammatory, antibacterial, antiviral, hypolipidemic, and hypoglycemic	—	
*Lycium barbarum* L.	Traditional Chinese medicine	Liver disease, hyperglycemia	Hypoglycemic	—	
*Magnolia officinalis* Rehder & E.H.Wilson	Traditional Chinese medicine	Vomiting and diarrhea, constipation, asthma, and cough	Anti-epilepsy, anti-depression, anti-dementia, anti-cerebral ischemia, anti-tumor, hypoglycemic	—	
*Ostrea gigas* Tnunb.	Traditional Chinese medicine	Insomnia, dizziness, spontaneous sweating, and night sweating	Antioxidant, anti-tumor, hypoglycemic	—	
*Polygonum tinctorium* Ait.	Traditional Chinese medicine	Sore throat, sore mouth, infantile convulsion, erysipelas, snakebite	Antibacterial, antiviral, anti-tumor, and antiallergic	—	
*Cordyceps sinensis* Berk.	Traditional Chinese medicine	Impotence, sore waist and knee, cough	Anti-oxidation, anti-tumor, and anti-aging	Guangdong province of China	1) Six arsenic speciation preparations, AsC, AsB, AsIII, DMA, MMA, and AsV were determined in fresh *Cordyceps sinensis* Berk. The linear relationship of the measured elements in their linear range is good (*r* > 0.9998), and the average recovery of the sample is 96.3%–105.5% 4% (RSD < 4.3%), the detection limits of six arsenic preparations were less than 0.15 ng/ml (Qian et al., 2019b)
					2) The main forms of arsenic in *Cordyceps sinensis* Berk. were MMA, DMA, AsC, AsB, AsIII, and AsV (Zuo et al., 2018)
*Ganoderma lucidum* (Curtis) P. Karst.	Traditional Chinese medicine	Insomnia, cough, asthma, excessive phlegm, asthenia, and fatigue syndrome	Anti-tumor, anti-aging, prevention and treatment of cardiovascular diseases, and protection of liver injury	—	Six selenium forms of Se (Ⅵ), Se (Ⅳ), SeMet, SeCys2, SeEt, and SeMeCys in *Ganoderma lucidum* (Curtis) P. Karst. were determined. The mass concentration of six selenium forms is 0.5–100.0 μg·L^−1^ has a linear relationship with its corresponding peak area, and the detection limit (3S/N) is 0.03–0.15 μg·L^−1^. The recovery was 91.7%–98.7%, and the relative standard deviation (*n* = 6) was 0.031%–3.2% (Zeng and Yao, 2020)

a Note: the origin of medicinal materials recorded in the table is collected from the articles.

If the origin of medicinal materials is not recorded in the articles, it is indicated by “—.”

**TABLE 12:** Application of HPLC-ICP-MS in Chinese patent medicine.

Name	Medical system	The main medicinal materials	Therapeutic use	Main pharmacological effects	Test results
Huoxue Zhitong Capsules	Traditional Chinese medicine	*Angelica sinensis* (Oliv.) Diels, *Boswellia sacra* Flück., Borneol, Natural copper, *Panax notoginseng* (Burkill) F.H.Chen	Traumatic injury, hemostasis, swelling, pain	Anti-inflammation and analgesia	Soluble arsenic and valence arsenic AsC, AsB, AsⅢ, DMA, MMA, and AsV in seven batches of Huoxue Zhitong Capsules were determined. Only AsⅢ was detected in six valence arsenic (Chen and Zhang, 2019)
Jiegu Qili tablets	Traditional Chinese medicine	*Angelica sinensis* (Oliv.) Diels, *Boswellia sacra* Flück., *Calamus draco* Willd. Ground beetle, Myrrh, *Rheum tanguticum* (Maxim. ex Regel) Balf.	Cataclasis	Improve hemorheology	The content of total arsenic in Jiegu Qili tablets was determined. The linear range of total arsenic mass concentration was 2.0–20.0 ng/ml (*r* = 0.9992). The detection limit was 0.0016 ng/ml. The recovery was 98.23%–99.95%, and the RSD was less than 3.5% 25% (*n* = 3). AsB, AsC, MMA, DMA, As Ⅲ, and As Ⅴ have good linear relationship (*r* > 0.999), the detection limit is 0.0029–0.0103 ng/ml, the recovery is 89.15%–95.5% 89%, and RSD is less than 7.14% (n = 3). Jiegu Qili tablet contains MMA, DMA, As Ⅲ, and As Ⅴ, in which the content of As Ⅴ is the highest, followed by As Ⅲ, and a small amount of MMA and DMA (Zhu et al., 2020)
Niuhuang Jiedu tablets	Traditional Chinese medicine	Bezoar, Borneol, *Glycyrrhiza uralensis* Fisch. ex DC., *Platycodon grandiflorus* (Jacq.) A.DC., Plaster, Realgar, *Rheum tanguticum* (Maxim. ex Regel) Balf., *Scutellaria baicalensis* Georgi	Sore throat, sore gums, sore tongue, swelling, and pain	Antioxidant, antiviral, anti-tumor, anticonvulsant, antiepileptic	The detection limits of AsB, DMA, As Ⅲ, MMA, and As Ⅴ were 0.05, 0.05, 0.08, 0.10, and 0.10 μg/L, respectively. Linear correlation coefficient *R* ^2^ > 0.999. The recovery was 86.3%–109.2%, and the RSD was less than 5% (Chen et al., 2017)
Xiaoer Kechuanling granules	Traditional Chinese medicine	*Ephedra equisetina* Bunge, *Glycyrrhiza uralensis* Fisch. ex DC., *Lonicera japonica* Thunb., Plaster, *Strobilanthes cusia* (Nees) Kuntze, *Prunus sibirica* L., *Trichosanthes kirilowii* Maxim.	Cough caused by upper respiratory tract infection	Antiviral, anti-inflammatory, antioxidant, and anticoagulant	The contents of Cr (Ⅲ) and Cr (Ⅵ) in Xiaoer Kechuanling granules were determined. Cr (Ⅲ) and Cr (Ⅵ) were in the range of 5–100 μg·g^−1^, *r* > 0.999, the recovery of Cr (VI) was 82.1%–90.4%. The recovery of Cr (Ⅲ) was 94.1%–95.2%. Cr (VI) was detected in eight batches of samples, the detection value was 0. 027–0. 082 μg·g^−1^, the average value is 0.051 μg·g^−1^, the detected value of Cr (Ⅲ) is 5.775–18.743 μg·g^−1^, and the average value is 10.366 μg·g^−1^ (Chen et al., 2020f)

## Discussion

This study describes the application of ICP-MS analysis of minerals and heavy metals in traditional drugs, including medicinal materials derived from plants, animals, minerals, and their preparations and the research and development of combined technology. Among them, medicinal materials derived from plants are divided into roots, stems, leaves, flowers, fruits and seeds, whole plants, and other medicinal materials derived from plants of medicinal parts. The content of effective components of traditional medicinal materials derived from plants is affected by soil, climate, and other environmental factors, so the quality of medicinal materials from different producing areas is different, which is also an important reason for the formation of genuine medicinal materials. Zuo et al. (2021) used ICP-MS to determine the residues of heavy metals and harmful elements in *Lycium barbarum* L. The average contents of lead, cadmium, arsenic, mercury, and copper in *L. barbarum* L. from three origins were 0.30, 0.066, 0.05, 0.003, 6.71 mg·kg^−1^, respectively. Cluster analysis and PCA showed that 33 batches of samples were divided into three groups, and the samples from the same origins were clustered into the same group. The results of self-organizing map clustering were consistent with those of PCA. Regularities between the distribution of *L. barbarum* L. and origins could be found. The results of the safety evaluation showed that the single factor index of lead, cadmium, arsenic, mercury, and copper in all the samples was less than 0.7, and the comprehensive pollution index ranged from 0.11 to 0.51, which indicated that the pollution situation was safe. The results of the risk assessment indicated that the health risk of heavy metals and harmful elements in samples from different origins was acceptable. As the medium of direct contact with medicinal materials, the soil is directly related to the composition and content of inorganic elements in traditional Chinese medicine and is one of the important ecological factors affecting the quality of medicinal materials. Medicinal materials play an important role in the soil environment. On the one hand, the social progress and the impact of human activities has caused soil pollution, the decline of soil fertility, the degradation of land quality, the reduction of the quality of medicinal materials, and the exceeding of harmful substances. On the other hand, the planting areas of medicinal materials have been expanded and changed arbitrarily, and some have exceeded the traditional real estate areas, so their ecological suitability is worth studying. ICP-MS combined with the statistical analysis method enriches the authentic theory of medicinal materials, greatly improves the theoretical understanding of medicinal materials derived from plant cultivation, and also reveals the important influence of the control and regulation of inorganic elements on the quality of original medicinal materials.

The efficacy of traditional drugs is the result of the synergy of multiple functional components contained in them. At present, the quality control of traditional drugs in China mainly includes appearance and microscopic identification for eliminating the false and preserving true, inspection of impurities and heavy metals for judging quality, and determination of the content of effective components directly related to function and effect, unable to characterize the integrity and complexity of preparations pharmacology and efficacy, so that the product quality cannot be effectively controlled, which seriously affects the clinical application of traditional drugs and restricts the development of new drug research and development and industry of traditional drugs. Traditional Chinese medicine theory emphasizes the overall effect of traditional drugs and attaches importance to the synergistic effect of various chemical components. Therefore, only taking one to two effective components in traditional drugs as quantitative and qualitative indicators can never reflect the internal quality of traditional Chinese medicine as a whole. It is necessary to establish a method to comprehensively evaluate the quality of traditional Chinese medicine as a whole. There is a correlation between the content of effective components and the content of inorganic elements in traditional drugs. Therefore, the detection of inorganic elements in traditional drugs is feasible to control their quality. Therefore, the elements and their contents in medicinal materials can be determined by ICP-MS, the inorganic element spectrum can be established, and the finishing quality of medicinal materials and Chinese inorganic elements can be evaluated in combination with data system analysis to achieve quality control. Secondly, for the quality control of the preparations, we should not only determine the inorganic elements of preparations but also analyze the inorganic elements of raw materials and medicinal materials to avoid exceeding the standard of harmful heavy metal elements from the source and then control the risk that may be introduced into the preparations. At present, the safety of traditional drugs has become a “bottleneck,” restricting the production and development of traditional drugs and gaining international recognition. Excessive heavy metal elements have an important impact on the activity and safety of medicinal materials, which has attracted extensive attention (Ji et al., 2014). ICP-MS is widely used in traditional medicine with its unique advantages and has achieved remarkable results, which has promoted the development of traditional medicine. Starting from the detection of soil and water quality, it ensures the safety of medicinal materials. The safety of clinical medication is ensured starting from the detection of medicinal materials and preparations. In addition, the pharmacodynamic material basis of some preparations may be related to trace heavy metals, so it can be comprehensively analyzed from the preparations, blood components, and tissue distribution components so that the application of ICP-MS can indirectly reveal the pharmacodynamic material basis. The limit standard of heavy metal elements measured by ICP-MS can fill the data gap of many traditional Chinese medicines and Tibetan medicine standards in the future and lay a foundation for the validation experiment of heavy metal monomers.ICP-MS can also be used to determine inorganic elements in biological samples (whole blood, serum, urine, lung, liver, and other tissues) to provide effective information for the exploration of drug action mechanisms and clinics. Liang et al. (2014) tested the changes of iron content in rat liver by ICP-MS, verified that d-limonene had a certain protective effect on iron coincidence caused by alcohol, and provided a reference for the study of alcoholic liver injury. Morton et al. (2017) used ICP-MS for multi-element analysis of human lung samples to determine the concentration of various elements in the collected lung samples and then used them for comparison with future clinical, environmental, nutritional, toxicological, and forensic investigations. Zhao et al. (2018) determined the spectrum of metal elements in patients’ serum by ICP-MS and found the very important metal elements in a specific infection in blood flow infection, which provided a basis for the diagnosis, prevention, and treatment of BSI from the perspective of metallography. The content, migration, and transformation of trace elements in organisms play an important role in physiological activities. The analysis of trace elements in cells is of great significance, which can understand the functional mechanism of trace elements in cells and organisms. Renqing Changjue is a traditional Tibetan medicine, which has been widely used to treat various gastroenteritis diseases. However, due to the toxic components in Renqing Changjue, its biosafety and toxicity still need to be explored, including various heavy metals. Therefore, Wang et al. (2020b) gavaged rats with different doses of Renqing Changjue, and the recovery observation period lasted for 15 days. The liver and kidney tissues were examined by histopathology, and the serum and urine samples were collected for ^1^H nuclear magnetic resonance (^1^H NMR) spectral analysis and biochemical analysis. ICP-MS was used to determine the content of Hg in urine and serum samples to evaluate the toxicity and elaborate on the toxicological mechanism of Renqing Changjue in order to provide a basis for safety evaluation of Renqing Changjue in clinical use. Song et al. (2021) used ICP-MS to determine the contents of 18 elements such as Mn, Cu, Sr, Pb, Au, and Hg in hepatic venous blood, abdominal aortic blood, brain, liver, kidney, hair, urine, and feces of rats 24 h after MCAO. The contents of Li in the brain increased, and the contents of Cr and Cd decreased. The content of Mn in the liver increased, and the content of Ni decreased. The contents of Ag and Cs in the kidney increased.

In recent years, the scientific community has paid increasing attention to metals belonging to the platinum group (PGM), including Ru, Rh, Pd, Os, Ir, and Pt. The effects of platinum group element complexes and some platinum divalent compounds on cancer and genotoxicity have been confirmed in microbial experiments (Melucci et al., 2022). Melucci et al. (2021) determined thallium in herbal medicines. Precision and trueness, expressed as relative standard deviation and relative error, respectively, were generally lower than 7% in all cases. Inorganic elements affect not only the growth and development of medicinal plants but also the constituent factors of effective components in medicinal materials (Wang et al., 2014). Traditional drugs contain rich kinds of inorganic elements, complex matrices, and large content differences. ICP-MS has the advantages of a wide linear range, high sensitivity, and high analysis efficiency. Therefore, this technology can well solve the analysis problems of inorganic elements in traditional Chinese medicine. Luo et al. (2015a) determined and analyzed the differences of inorganic elements before and after processing *R. multiflora* (Thunb.) Moldenke by ICP-MS. After processing, the contents of Al, Fe, K, Mg, Mn, and Zn increased, whereas the contents of As, Pb, and Cd decreased significantly. It is suggested that the physiological functions of Al, Fe, K, Mg, Mn, and Zn elements are consistent with the effects of *R. multiflora* (Thunb.) Moldenke, which are beneficial to the liver and kidney, benefiting essence and blood enhancing immunity and anti-aging. The content of these elements increased significantly after processing, which may be related to the enhancement of the tonic effect of *R. multiflora* (Thunb.) Moldenke. The heavy metals As, Pb, and Cd are harmful to the human body and decrease significantly after processing, which may be one of the reasons for the detoxification of *R. multiflora* (Thunb.) Moldenke processed. With the continuous popularization and application of the inorganic element spectrum and statistical analysis and evaluation model, the research on the overall quality control of traditional Chinese medicine will be perfect. At the same time, with the development of high-performance liquid chromatography, ion chromatography, and ICP-MS, the analysis methods for different valence states of inorganic elements will become more and more mature. In addition, ICP-OES is also a means of determining metal elements. ICP-OES detects the intensity of the atomic characteristic emission spectrum. In the atomic emission spectrum, each element has multiple characteristic spectral lines. The spectrometer can automatically correct the function to reduce the background signal and screen out two to three analysis wavelengths with low detection limit, high sensitivity, and small interference for qualitative and quantitative analysis. Although the detection limit of ICP-OES is lower than that of ICP-MS, it has met the detection needs of various drugs, and its technology is mature, stable, economical, and convenient. It can be used as a detection method complementary to ICP-MS. Shen et al. (2019) determined 29 kinds of inorganic elements in samples of *Paris daliensis* H. Li et V. G. Souku and *P. dulongensis* H. Li et S. Kuritap produced in different regions to measure the content of 10 key inorganic elements: Cr, Mn, Fe, Cu, Hg, Zn, As, Sr, Cd, and Pb. Under the experimental conditions, elements were not related to each other, and many kinds of elements could be measured at the same time; toxic and heavy metals in samples of *P. daliensis* H. Li et V. G. Souk, and *P. dulongensis* H. Li et S. Kuritap did not exceed the limit. Hg was not detected in all samples. Zhong et al. (2019) determined the content of 20 inorganic elements in 18 samples of roots, stems, and leaves of *A. lappa* L. produced in different areas. Twenty kinds of inorganic elements in the samples of *A. lappa* L. roots contained rich elements essential for human beings such as K, Ca, Na, P, and trace elements Cu, Fe, Zn. Heavy metal Pb, As, Cu, and Cd in *A. lappa* L. samples did not exceed the limit. Hg was not detected in all 12 samples. Heavy metals in *A. lappa* L. roots harvested in 12 months did not exceed the limit. Fang et al. (2007) used HPLC-ICP-MS to analyze the forms of As in nine kinds of traditional Chinese medicine preparations, such as Niuhuang Jiedu tablets, Bushen tablets, Shiertaibao pills, Baoyingdan, Mingyan pills, Zhengxin pills, and Biminqing. An anion exchange column was used to determine the content of 0.5%. The aqueous solutions of 2 mmol/L EDTA and 2 mmol/L NaH_2_PO_4_ were used as mobile phases. Four forms of As Ⅲ, As V, MMA, and DMA were successfully separated and determined. The results showed that the form of As in the sample was mainly toxic inorganic arsenic and the amount of organic arsenic was low. Li et al., 2012 analyzed six different valence arsenic by HPLC-ICP-MS and studied the forms of soluble arsenic in Realgar and five different preparations of Realgar-containing preparations. The results showed that the acid-soluble arsenic of Realgar and its preparations was far less than its total arsenic content, and the existence of other components in the preparations may inhibit the dissolution of toxic forms of soluble arsenic. The determination of multivalent elements not only enriches the application scope of ICP-MS but also makes the research on the complex system of traditional drugs more in depth to lay a more solid foundation for the relationship between components and efficacy and clinical application research. At present, although the overall research on inorganic components in traditional drugs has attracted attention, the research on the process of elements entering the human body and the interaction between elements in the body is still relatively lacking. Nowadays, the analysis of inorganic elements in traditional Chinese medicine is mostly supplemented by other factors and indicators, and it is difficult to clarify the role of elements themselves. The mode of Metallomics refers to the research ideas of metabolomics, which is conducive to a more in-depth study of the role of inorganic elements and finding the specific targets and sites of some elements in the body. ICP-MS and its combined technology have many reports on the study of the relationship between trace elements and efficacy, the relationship between element morphology and toxicology, the determination of element content, and inorganic fingerprint of traditional drugs. However, in addition to the determination of element content, the research in other aspects is not deep enough.
